# Synthesis of Phenaliporphyrin, a PAH-Porphyrin Hybrid, from an Acenaphthene-Fused Cyclopropane Dialdehyde

**DOI:** 10.1021/acs.joc.5c02277

**Published:** 2025-12-01

**Authors:** Sunday Oladapo Jacob, Emily D. Harris, Melissa A. Mathius, Deyaa I. AbuSalim, Gregory M. Ferrence, Timothy D. Lash

**Affiliations:** † Department of Chemistry, 6049 Illinois State University, Normal, Illinois 61790-4160, United States; ‡ Department of Chemistry, Rowan University, Glassboro, New Jersey 08028, United States; § STEM Department, Rowan College of South Jersey, Vineland, New Jersey 08360, United States

## Abstract

Phenaliporphyrin, a new aromatic benziporphyrin derivative, has been synthesized from an annulated cyclopropane dialdehyde intermediate. Corey-Chaykovsky cyclopropanation of dimethyl acenaphthylene-1,2-dicarboxylate gave a cyclopropane diester, and this was converted to the corresponding bis-Weinreb amide. Reduction with lithium aluminum hydride afforded an unstable dialdehyde and subsequent acid-catalyzed condensation with a tripyrrane dicarboxylic acid, followed by oxidation with aqueous ferric chloride, gave the targeted porphyrinoid system. This involves ring expansion of the acenaphthylene precursor to generate the phenalene unit. Phenaliporphyrin exhibits a porphyrin-like UV–vis spectrum with a Soret band at 443 nm and a relatively intense Q-band at 601 nm. The proton NMR spectrum demonstrated that the system has a strong diamagnetic ring current, and the external *meso*-protons showed up downfield at 9.64 and 9.34 ppm while the internal C–H appeared upfield at −6.48 ppm. Although DFT calculations show that the phenalene ring is twisted to relieve steric interactions, single crystal X-ray diffraction analysis indicated that the macrocycle is nearly planar. The discrepancy was attributed to crystal packing forces and a distorted conformation is likely to be favored in solution. The aromatic properties for phenaliporphyrin were confirmed by nucleus independent chemical shift (NICS) calculations and anisotropy of induced current density plots.

## Graphical abstract

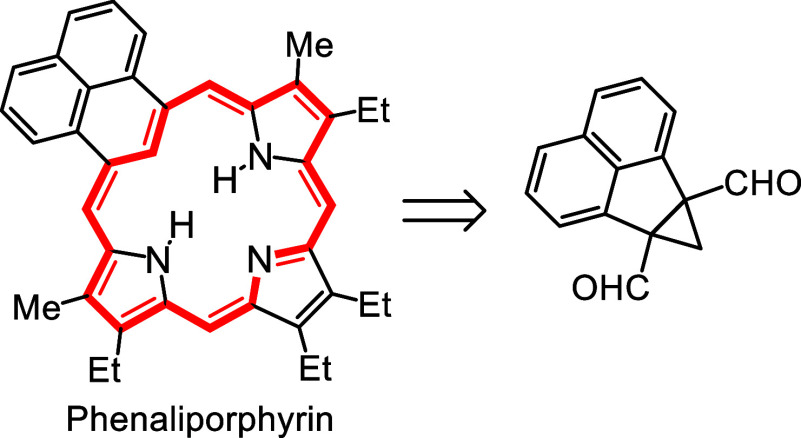

## Introduction

Porphyrin analogues with carbocyclic rings in place of a pyrrolic subunit, generally known as carbaporphyrinoid systems,^1^ are widely studied due to their unusual reactivity^2^ and ability to generate stable organometallic derivatives.^3,4^ These include true carbaporphyrins such as **1** and **2**, tropiporphyrins **3**, azuliporphyrins **4** and benziporphyrins **5** (Figure 1).^1^ In addition, N-confused porphyrins **6** are a closely related family of porphyrinoids with an inverted pyrrole subunit.^5^ The aromatic properties of these systems varies considerably and while carbaporphyrins **1** and **2** have highly aromatic characteristics that are comparable to the porphyrins, this is diminished in tropiporphyrins and azuliporphyrins, and benziporphyrins are essentially devoid of global aromatic character.^6^ The presence of a *m*-phenylene unit in benziporphyrins interrupts the macrocyclic conjugation pathways available in **1**–**3** and this can only be overcome by undermining the 6π arene character of the benzene subunit. As the resonance stabilization energy for a benzene unit within naphthalene is less than that for benzene itself, insertion of a naphthalene into a porphyrin-type skeleton might be expected to have a better chance of taking on global aromatic properties but this turned out not to be the case.^7^ Similarly, insertion of a pyrene unit into this type of structure to give pyreniporphyrin **7** ^8^ also failed to support porphyrinoid aromaticity. Protonation of benziporphyrins, naphthiporphyrin or pyreniporphyrin gave rise to weak diatropic ring currents,^6^ and electron-donating groups also enhanced the overall aromatic characteristics, but these properties remained insignificant compared to true porphyrins, carbaporphyrins **1** and **2**,^1^ or N-confused porphyrins **6**.^5^

**Figure 1:**
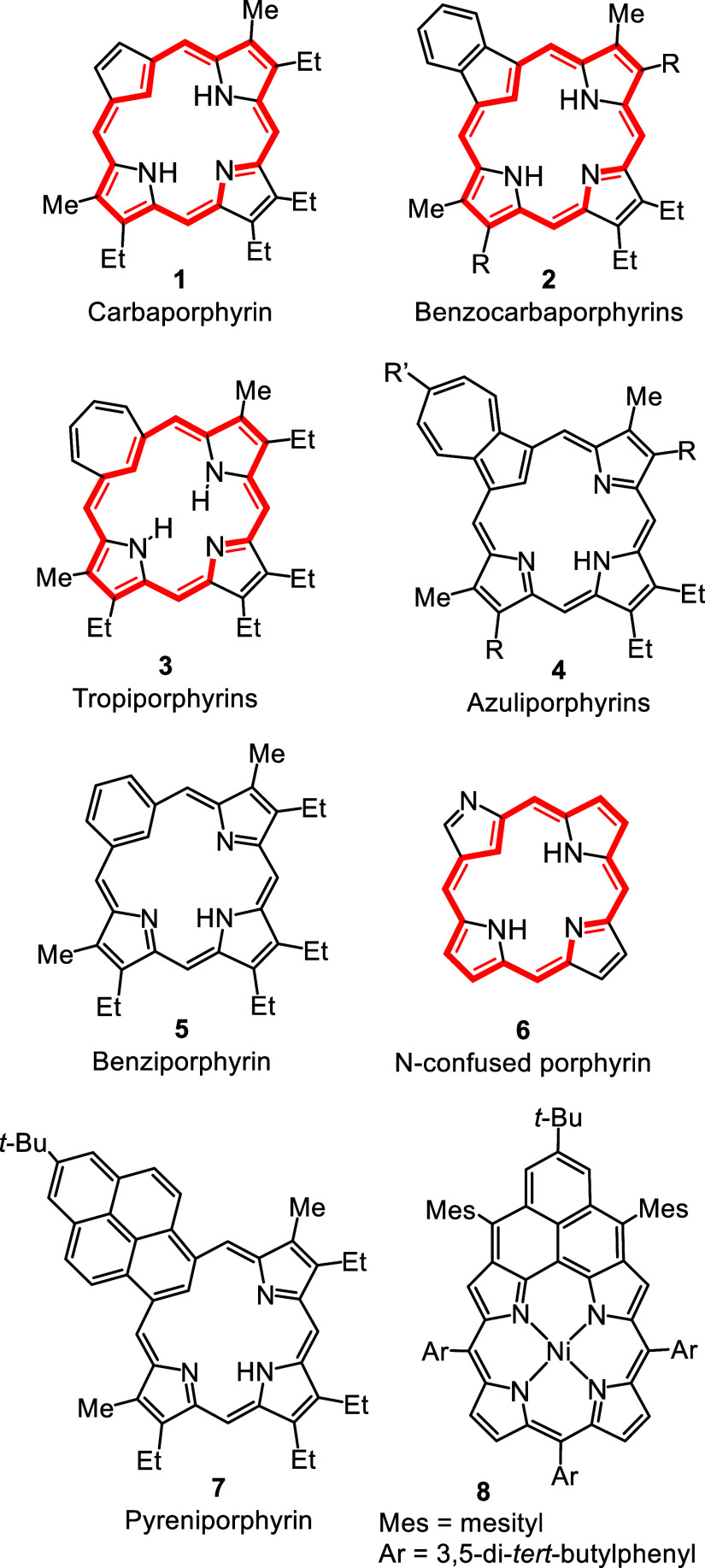
Selected structures of porphyrin analogues.

Although insertion of naphthalene or pyrene into the porphyrin framework blocks global aromaticity, we speculated that phenalene could provide an alternative polycyclic aromatic hydrocarbon (PAH) subunit that has the potential to overcome these difficulties (Figure 2). Phenalene is an unusual hydrocarbon structure that readily forms anions, cations and radical species.^9,10^ Unlike naphthalene and pyrene, one of the carbon atoms must be sp^3^ hybridized. Porphyrins such as structure **8** with fused phenalene units have been reported previously,^11^ but carbaporphyrin-type structures **9** incorporating phenalene units were not known. Heterographene structures related to the porphyrins are currently the focus of numerous investigations^12–17^ and structure **9** represents an intriguing architecture that could lead to further developments in this area. In this paper, the first example of a phenaliporphyrin **9** (Scheme 1) is reported. This was accomplished by a novel synthetic strategy from an acenaphthene-fused cyclopropane dialdehyde.

**Figure 2:**
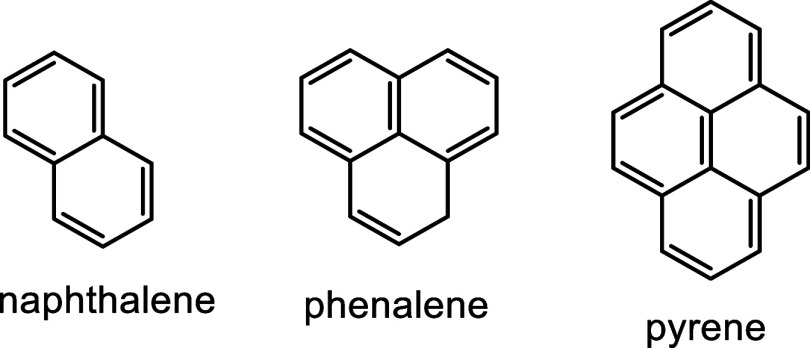
Selected polycyclic aromatic hydrocarbons.

**Scheme 1:**
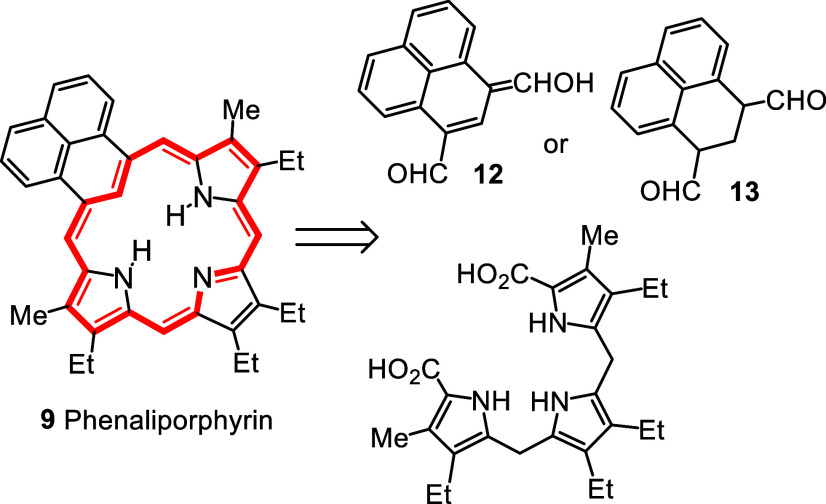
Retrosynthetic Analysis of Phenaliporphyrin **9**

## Results and Discussion

MacDonald-type “3 + 1” condensations have been successfully applied to the synthesis of diverse porphyrinoid systems including structures **1**–**5** and **7** (Figure 1).^18^ This involves reacting a tripyrrolic intermediate (tripyrrane) with suitable dialdehydes and we anticipated that phenaliporphyrin could be prepared from phenalene dialdehyde **12** or the related dihydrophenalene **13** (Scheme 1). However, considerable difficulties were encountered in our attempts to prepare these precursors. In an attempt to install a methoxyalkene unit (Scheme 2), perinaphthenone (phenalenone, **14**) was reacted with (methoxymethyl)­triphenylphosphonium chloride and potassium *tert*-butoxide.^19^ It was speculated that the anticipated product, methoxyphenafulvene **15**, would undergo Vilsmeier–Haack formylation and following hydrolysis could yield **12**. However, all attempts to carry out Wittig condensations with **14** were unsuccessful and primarily resulted in decomposition. A review of the literature indicated that structurally related phenafulvenes are unstable^20^ and it was concluded that this approach was not viable.

**Scheme 2:**
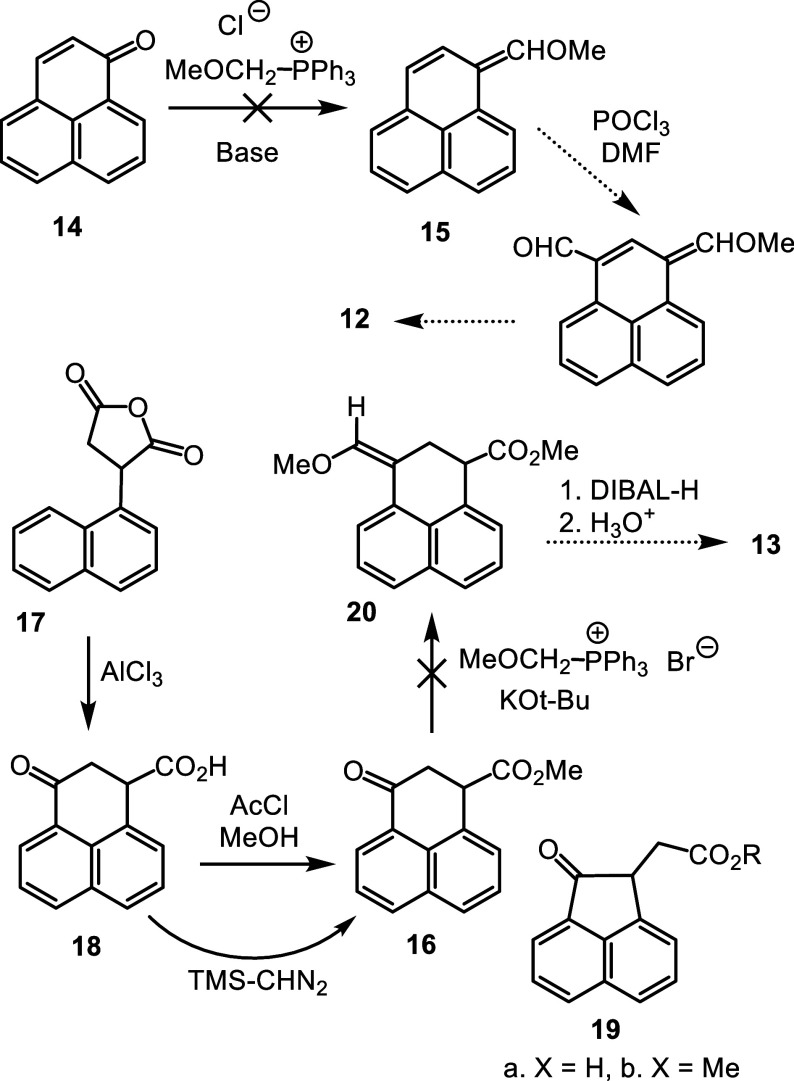
Attempted Syntheses of Phenalene Dialdehydes

An alternative route was designed using keto ester **16** (1-naphthyl)­succinic anhydride (**17**) was prepared in 6 steps from 1-tetralone following literature procedures (Scheme 2).^21–23^ Cyclization of **17** with 4 equiv. AlCl_3_ afforded keto acid **18** as the major product together with a minor isomeric 5-membered ring byproduct **19a**.^23^ This was taken on to give the related keto ester **16**. Mild conditions were required and initially this was accomplished with 2 equiv acetyl chloride in methanol.^24^ Superior yields were subsequently obtained using trimethylsilyldiazomethane^25^ and under these conditions **16** was isolated in ca. 80% yield. Unfortunately, this intermediate failed to give the required methoxyalkene **20** with (methoxymethyl)­triphenylphosphonium chloride and KO*t*-Bu or *n*-BuLi.^26^ The transformation was also attempted with methoxymethyl­(diphenyl)­phosphine oxide^27^ with KO*t*-Bu or lithium diisopropylamide (LDA), but this gave no sign of the required product. In each case, a significant amount of starting material was recovered, although some degradation was observed. Similarly negative results were obtained using the Horner-Wadsworth-Emmons reaction^27,28^ when **16** was treated with diethyl (methoxymethyl)­phosphonate and KO*t*-Bu. It is likely that the presence of an enolizable proton in **16** interferes with this chemistry and it became necessary to seek an alternative route to the phenaliporphyrin system.

It was hypothesized that acenaphthene-fused cyclopropane dialdehyde **21** could act as a synthon for proposed intermediate **12** (Scheme 3). Deprotonation of the cyclopropane unit could trigger ring expansion to afford the phenalene dialdehyde. Dialdehyde **21** might be prepared from a related diester, and it was anticipated that the three-membered ring could be introduced using the Corey-Chaykovsky methodology.^29^ Radical bromination of acenaphthene with *N*-bromosuccinimide (NBS) and benzoyl peroxide give dibromoacenaphthylene **22** ^30^ and this undergoes the Rosenmund-von Braun reaction with copper­(I) cyanide in *N*-methyl-2-pyrrolidinone at 160 °C to afford dinitrile **23** (Scheme 4).^30,31^ Acid-catalyzed hydrolysis with sulfuric acid in a refluxing 1:1 acetic acid–water mixture gave the related dicarboxylic acid **24** ^30^ and this underwent a Fischer esterification to afford dimethyl ester **25** in 76% yield.

**Scheme 3:**
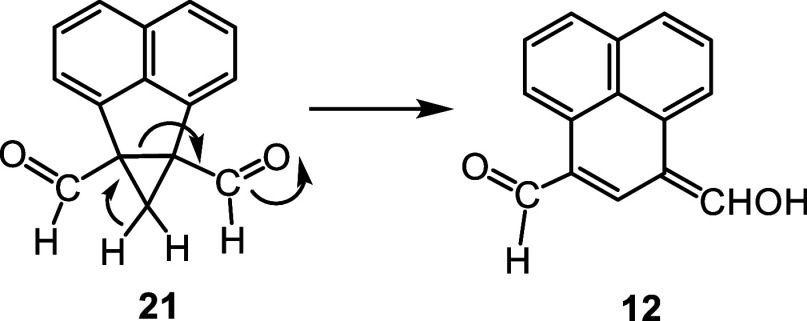
Proposed Acenaphthene-Fused Cyclopropane Dialdehyde as an Alternative Intermediate

**Scheme 4:**
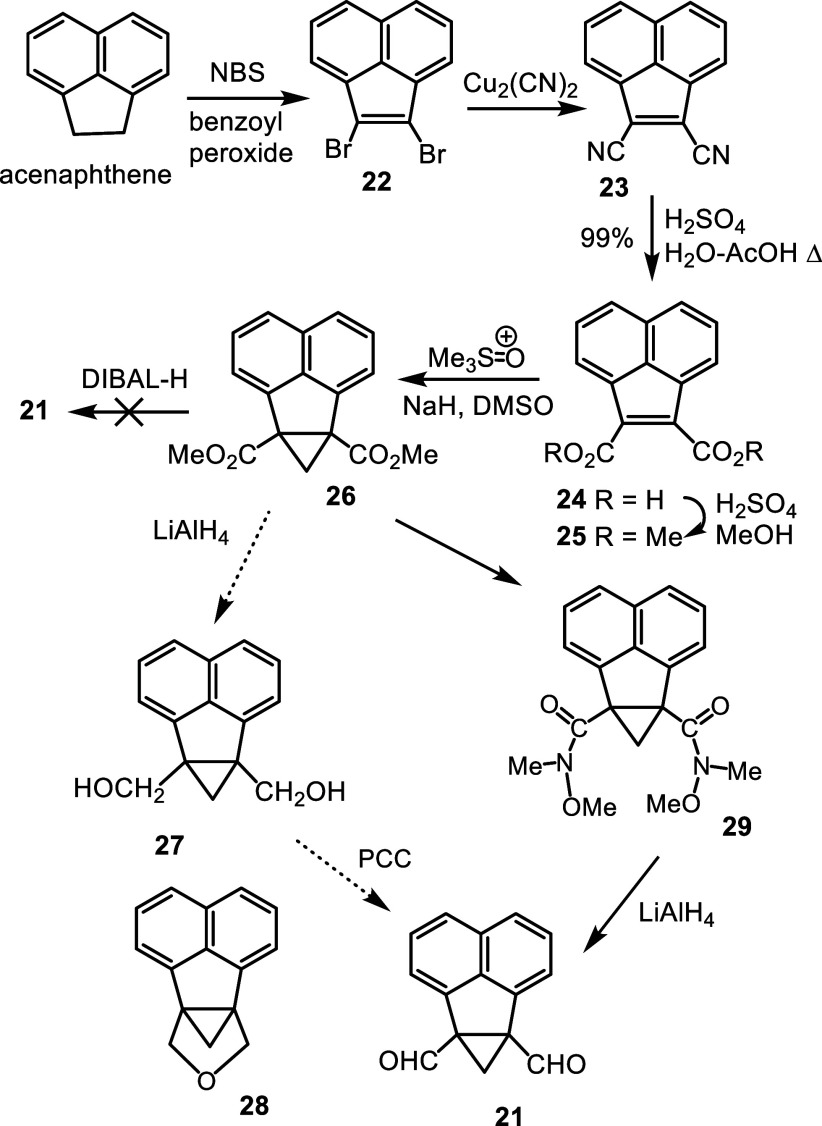
Synthesis of Cyclopropane Dialdehyde **21** From Acenaphthene

A solution of **25** in DMSO was treated with 1.5 equiv trimethyl sulfoxonium iodide and 1.5 equiv sodium hydride and this gave cyclopropane derivative **26** in quantitative yield. However, attempts to reduce the diester with diisobutylaluminum hydride (DIBAL-H) were unsuccessful and unreacted starting material was recovered. Instead, **26** was reduced with lithium aluminum hydride with the expectation that dialcohol **27** would be generated. The resulting product could not be purified, although the proton NMR spectrum was consistent with **27**. However, high resolution ESI-MS gave a [M + H]^+^ peak at *m*/*z* 209.0959, which corresponds to C_15_H_13_O ^+^ (calculated *m*/*z* 209.0961) suggesting, in conjunction with the NMR data, that this species actually corresponds to the cyclic ether **28**. The identity of this product is not clear but attempts to oxidize the material with pydidinium chlorochromate (PCC) did not afford the required dialdehyde. As an alternative, diester **26** was converted into the bis-Weinreb amide **29** by reacting it with *N*,*O*-dimethylhydroxylamine hydrochloride and isopropylmagnesium chloride. The reliability of reducing Weinreb amides with lithium aluminum hydride to selectively produce aldehydes is well established,^32^ and this turned out to be the case for **29**. Reduction of **29** with LiAlH_4_ gave a virtually quantitative yield of dialdehyde **21**, but this proved to be highly unstable, and it was necessary to immediately take it on to the next step.

The crude dialdehyde was condensed with tripyrrane dicarboxylic acid **30** ^33,34^ in the presence of trifluoroacetic acid (TFA) and the reaction mixture was subsequently oxidized with an aqueous ferric chloride solution (Scheme 5). Following purification by column chromatography and recrystallization from chloroform–methanol, the long sought after phenaliporphyrin **9** was isolated in 10% yield. The new carbaporphyrinoid structure afforded intense green colored solutions and also gave dark green colored crystals. Ring expansion must occur at some point during the reaction, as illustrated for structure **31**, but this may occur prior to macrocycle formation.

**Scheme 5:**
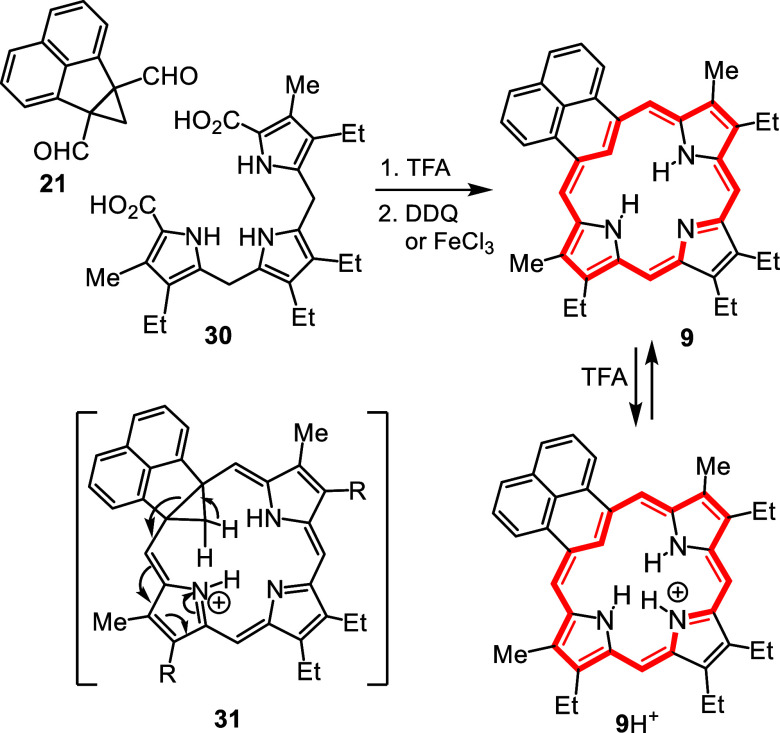
Synthesis of Phenaliporphyrin

The UV–vis spectrum for phenaliporphyrin was porphyrin-like with a Soret band at 443 nm but gave a relatively intense Q-band at 601 nm (Figure 3). Addition of 1 equiv of TFA led to the formation of a monoprotonated species **9**H^+^ that exhibited a bathochromically shifted Soret band at 454 nm (Figure 3). However, while further addition of TFA led to additional changes, the resulting protonated species were unstable and rapidly decomposed. The proton NMR spectrum of **9** in CDCl_3_ confirmed the presence of a strong diatropic ring current and the *meso*-protons gave two 2H singlets at 9.64 and 9.34 ppm (Figure 4). Although the resonance for the internal NH protons was not observed, the inner C–H gave a singlet upfield at −6.48 ppm. The difference in chemical shifts (Δδ), which is often used as an indicator of aromatic character, was just over 16 ppm. The methyl resonances gave a 6H singlet at 3.43 ppm, which is slightly upfield from the equivalent protons in porphyrins or carbaporphyrins with methyl substituents. Although the shifts are not as large as those seen in carbaporphyrins such as **1** and **2**, it can still be concluded that phenaliporphyrin supports strongly aromatic characteristics. Addition of a trace amount of TFA to the NMR solution induced the *meso*-proton resonances to shift downfield to 9.91 and 9.56 ppm but the inner C–H also shifted downfield to −5.68 ppm. The NH protons for **9**H^+^ were observed at −0.70 ppm and −3.45 ppm. The Δδ value for this species is 15.59 ppm, indicating a slight reduction in the diamagnetic ring current. The {^1^H}^13^C NMR spectrum of **9** gave resonances at 95.2 and 99.6, values that are typical for porphyrins and carbaporphyrins. The internal CH was located at 114.6 ppm. Both the proton and {^1^H}^13^C NMR spectra confirmed that *a* plane of symmetry was present, and the identity of the structure was confirmed by high resolution TOF-ESI-MS.

**Figure 3:**
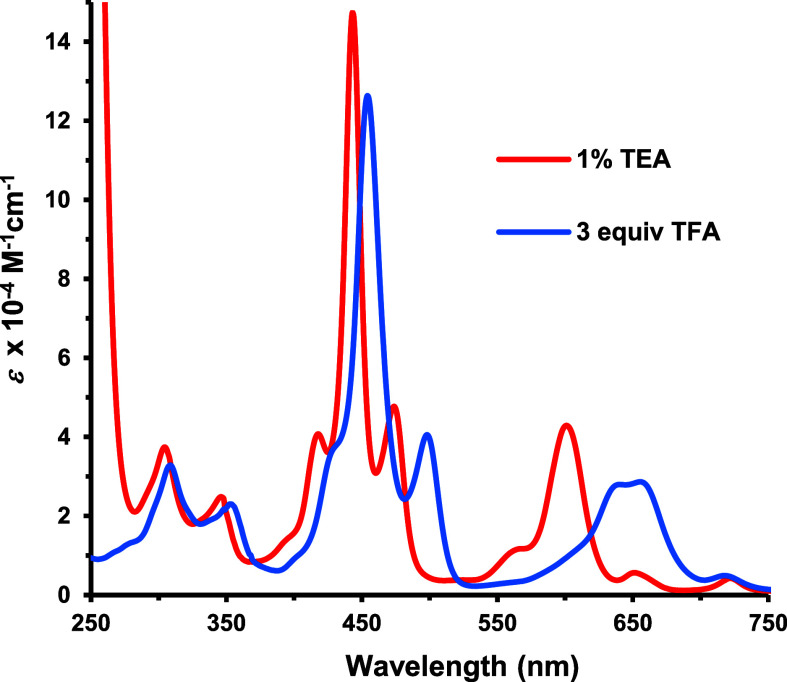
UV–vis spectra of phenaliporphyrin **9** in 1% triethylamine-CH_2_Cl_2_ (red line) and with 3 equiv TFA in CH_2_Cl_2_ (**9**H^+^, blue line).

**Figure 4:**
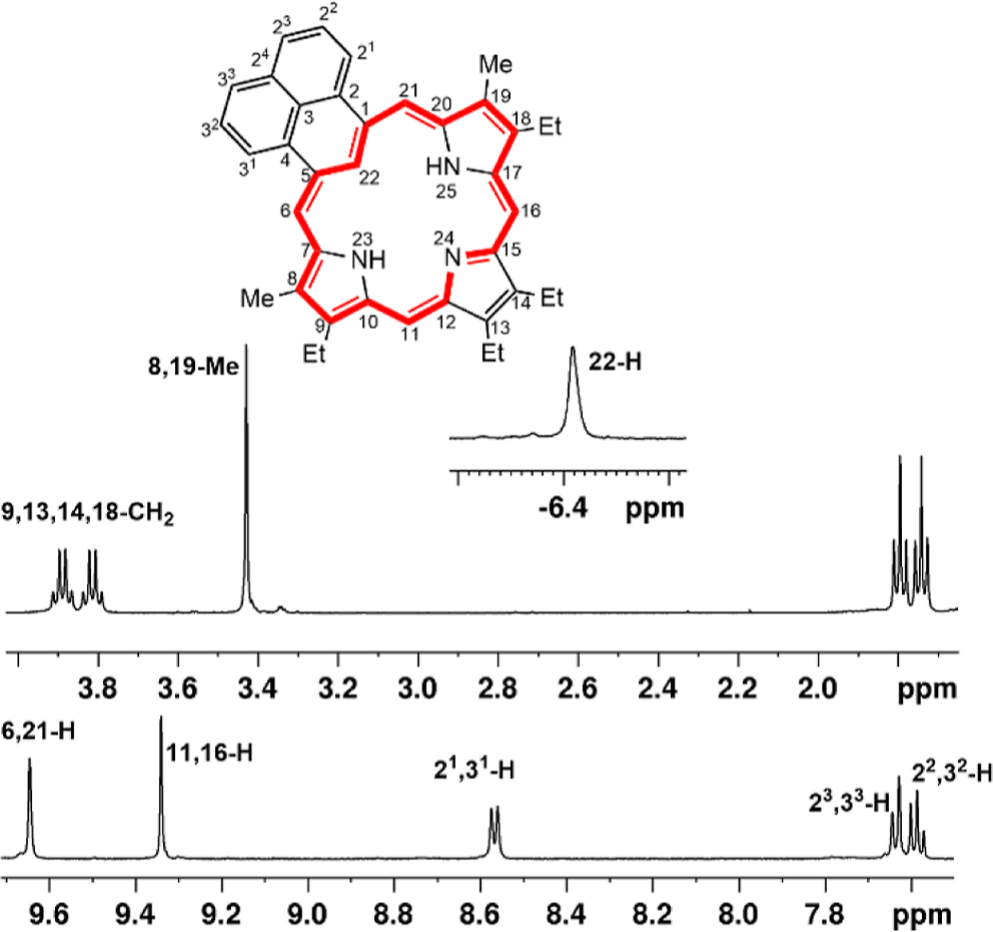
Proton NMR spectrum of phenaliporphyrin in CDCl_3_.

Crystals of phenaliporphyrin **9** suitable for X-ray crystallographic analysis were obtained and the results showed a nearly planar overall framework geometry quantified by a 0.107 Å root mean squared deviation of the 32 non-hydrogen framework atoms (excluding methyl and ethyl groups) from the plane defined by those atoms. Given the close proximity of H atoms on the adjacent *meso*-carbon atoms to two of the phenalene H atoms, the phenalene moiety is remarkably coplanar with the macrocyclic plane (Figure 5). This is illustrated by the 4.13(3)° interplanar angle between the plane defined by the 13 phenalene C atoms and the plane defined by atoms (C1, C2, C3, C4, C5, C6, C7, C8, C9, C10, C11, C14, C15, C16, C17, C18, C19, C20 and C21) (Figure S47). The remainder of the macrocyclic framework is rather flat with the two pyrrole rings adjacent to the phenalene subunit and the pyrrole ring opposite the phenalene subunit only tilted 3.36(8)°, 9.24(8)° and 1.59(8)°, respectively, from the macrocyclic plane. The long 1.480(1) Å C1–C2 and 1.481(1) Å C4–C5 bond lengths are consistent with more single bond like character which suggests the naphthalene pi system of the phenalene subunit is isolated from the main macrocyclic pi system. This is further supported by a Mogul geometry check indicating the 1.408(2) Å C1–C21 and 1.406(2) Å C5–C6 bond lengths are shorter and the 1.391(2) Å C6–C7 and 1.394(2) Å C20–C21 bond lengths are longer than typical. Similarly, the long 1.461(1) Å C12–C13 and 1.462(1) Å C14–C15 bond lengths and relatively short 1.360(2) Å C13–C14 bond length indicate a disconnection of the β C atoms of the pyrrole ring opposite the phenalene subunit from the main macrocyclic pi system. With the exception of the discussed bond lengths, a Mogul geometry check validated all other bond distances, and most angles and torsions to be within typical ranges.^35^

**Figure 5:**
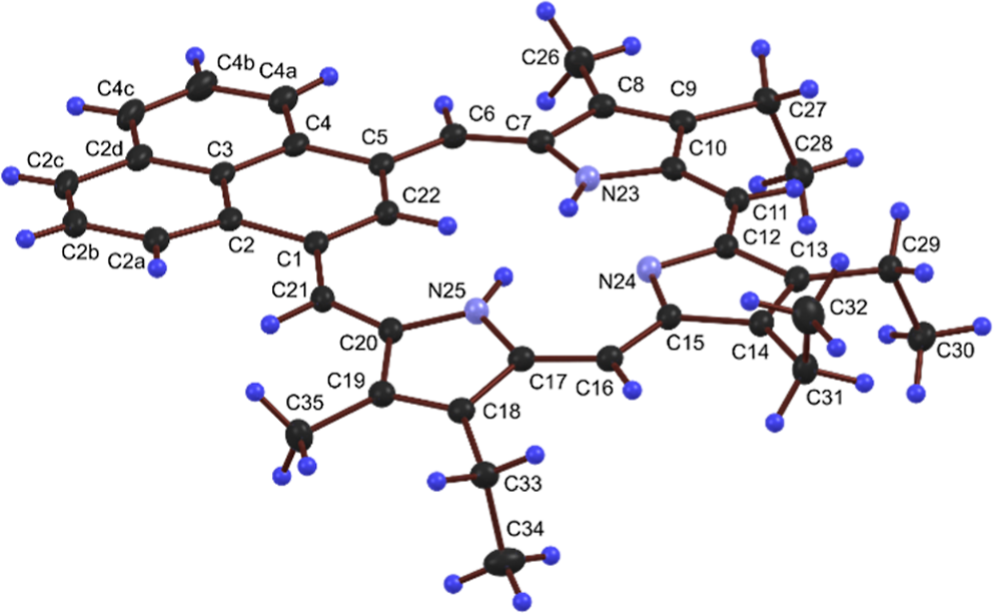
Color POV-Ray rendered ORTEP III drawing (50% probability level, hydrogen atoms rendered arbitrarily small for clarity) of phenaliporphyrin **9**.

The crystallographic data is consistent with the main residue being primarily (93%) composed of 9,13,14,18-tetraethyl-8,19-dimethylphenaliporphyrin (**9**) and the remaining 7% being composed of one or more related hexaalkylphenaliporphyrins. Additional details regarding the refinement may be found in the cif. The crystallographically observed atomic occupancies are chemically consistent with small degree of rearrangement to the pyrrole units within the tripyrrane precursor under the synthetic conditions for macrocycle formation. Acidolytic cleavage-recombination processes of this type are well documented but are not usually significant under the TFA-catalyzed condensation conditions used in this study.^18,34^ The proton NMR spectrum for **9** indicates that only small amounts (ca. 5%) of these contaminants are present, but they are nevertheless apparent in the crystallographic data. The exact composition cannot be determined from these results, but the simplest assignment is the presence of one additional isomer, namely 8,9,13,18-tetraethyl-14,19-dimethylphenaliporphyrin, as the remaining 7% of the main residue’s composition.

### Computational Studies

Density functional theory (DFT) calculations^36–39^ were used to gain better insights into the phenaliporphyrin system. Four tautomers of unsubstituted phenaliporphyrin were investigated, together with selected protonated species (Tables 1 and 3). The structures were optimized using M06–2X with the triple-ζ basis set 6-311++G­(d,p). In the free base tautomers, two protons are moved about within the cavity (positions 22–25) and are designated as **PhP**-**23**,**25-H**, **PhP**-**23**,**24-H**, **PhP**-**22**,**24-H**, and **PhP**-**22**,**23-H** (Table 1). The energies (Δ*E*) for these tautomers were compared, and the relative Gibbs free energies were determined. Tautomer **PhP**-**23**,**25-H** was shown to be the most stable, although **PhP**-**23**,**24-H** was <4 kcal/mol higher in energy (Table 1). This species is less favored because the internal hydrogens in **PhP**-**23**,**25-H** are better situated for hydrogen-bonding interactions. Tautomers **PhP**-**22**,**24-H** and **PhP**-**22**,**23-H** have internal methylene units and are much less stable, in part due to disruption of the aromatic 18π electron delocalization pathways. All four tautomers exhibit distortion to the macrocyclic structure, even in the case of favored tautomer **PhP**-**23**,**25-H** which displays a twisted phenylene unit (Table 2). This is significantly different from the X-ray crystal structure for **9** as this shows a flat phenylene ring and the overall structure is far closer to being planar (Figure 5). The differences are attributed to crystal packing forces that flatten out the phenylene subunit. Twisting of the phenalene unit results from steric interactions with the adjacent *meso*-hydrogen atoms and the results imply that this conformation is favored in solution. Nevertheless, the calculated bond lengths for **PhP**-**23**,**25-H** (Figure S49) are in good agreement with the X-ray diffraction data for **9** (Tables S1 and S4).

**Table 1:**
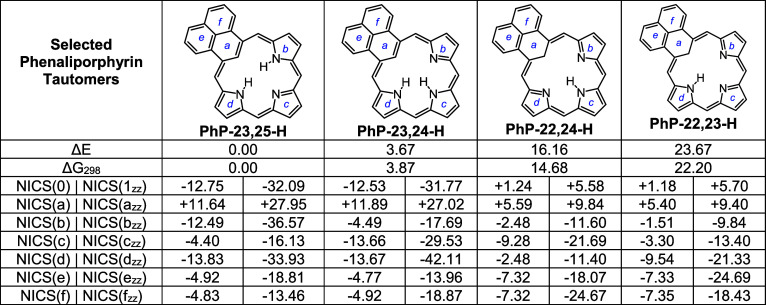
Calculated Relative Energies and NICS Values for Phenaliporphyrin Tautomers

**Table 2:** Calculated Dihedral Angles for Phenaliporphyrin Tautomers

	ab	bc	cd	da	ea	fa	average dihedral
**PhP**-**23**,**25-H**	–17.43	4.39	–0.74	4.09	–14.85	–16.32	9.64
**PhP**-**23**,**24-H**	–2.99	3.36	–9.98	18.91	16.94	11.73	10.65
**PhP**-**22**,**24-H**	11.59	–3.29	–3.29	11.59	26.22	26.21	13.70
**PhP**-**22**,**23-H**	–11.10	5.61	1.53	–12.31	–25.67	–25.69	13.65
**PhPaH** ^+^	–27.98	18.61	–18.61	27.95	12.56	–12.64	19.72
**PhPbH** ^+^	–14.09	5.36	5.34	–14.07	–26.51	–26.53	15.32
**PhPcH** ^+^	–10.41	–1.91	11.63	–18.29	–27.79	–23.68	15.62
**PhPaH**_2_^2+^	16.09	–24.45	24.44	–16.06	–17.36	17.42	19.30
**PhPbH**_2_^2+^	45.60	–30.89	19.55	–30.04	–7.37	2.74	22.70
**PhPcH**_2_^2+^	–32.30	31.68	–44.95	42.96	14.58	–11.48	29.66

Nucleus-independent chemical shift (NICS) calculations^40^ were used to determine the diatropic character of the phenaliporphyrin tautomers (Table 1). NICS calculations give a large negative value in the center of aromatic structures, while a large positive result may indicate antiaromaticity. Values close to zero indicate that the system is nonaromatic. Standard NICS calculations consider the effects due to σ and π electrons, while NICS_*zz*_ calculations mostly measure the effects due to the π system. The latter is generally considered to be more reliable. In this work, both NICS and NIC(1)_*zz*_ calculations were carried out on all of the structures. The NICS_*zz*_ calculations were performed 1 Å above the ring (NICS(1)_*zz*_). Even though the values determined using NICS(1)_*zz*_ are larger than those obtained for standard NICS calculations, the same trends were noted using both methods. The NICS and NICS(1)_*zz*_ values for tautomers with an internal methylene group gave small positive values, indicating that these structures are nonaromatic. However, **PhP**-**23**,**25-H** and **PhP**-**23**,**24-H** gave large negative NICS_*zz*_ values of −32.09 and −31.77 ppm, respectively, demonstrating that these structures are strongly aromatic (Table 3). The NICS and NICS(1)_*zz*_ values for the individual rings (a-f) were also calculated and these provide insights into the favored conjugation pathways for these systems. The results for **PhP**-**23**,**25-H** and **PhP**-**23**,**24-H** are consistent with conventional 18π electron delocalization pathways (Figure 4). Ring *a* gave a NICS(1)_*zz*_ values of +27.95 and +27.02 ppm for **PhP**-**23**,**25-H** and **PhP**-**23**,**24-H**, respectively. This result is consistent with this unit being deshielded due to it lying next to the macrocyclic 18π electron circuit and the naphthalene system, and the result does not imply the presence of antiaromatic character. Anisotropy of induced current density (AICD) plots^41^ for these tautomers are also consistent with this interpretation and the result for tautomer **PhP**-**23**,**25-H** is illustrated in Figure 6.

**Table 3:**
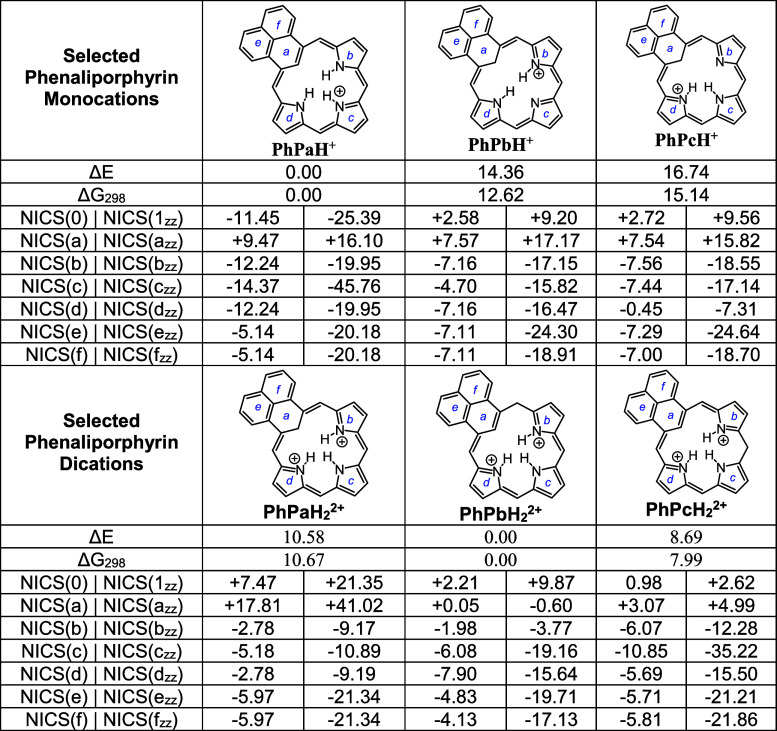
Calculated Relative Energies and NICS Values for Selected Mono- and Diprotonated Phenaliporphyrins

**Figure 6:**
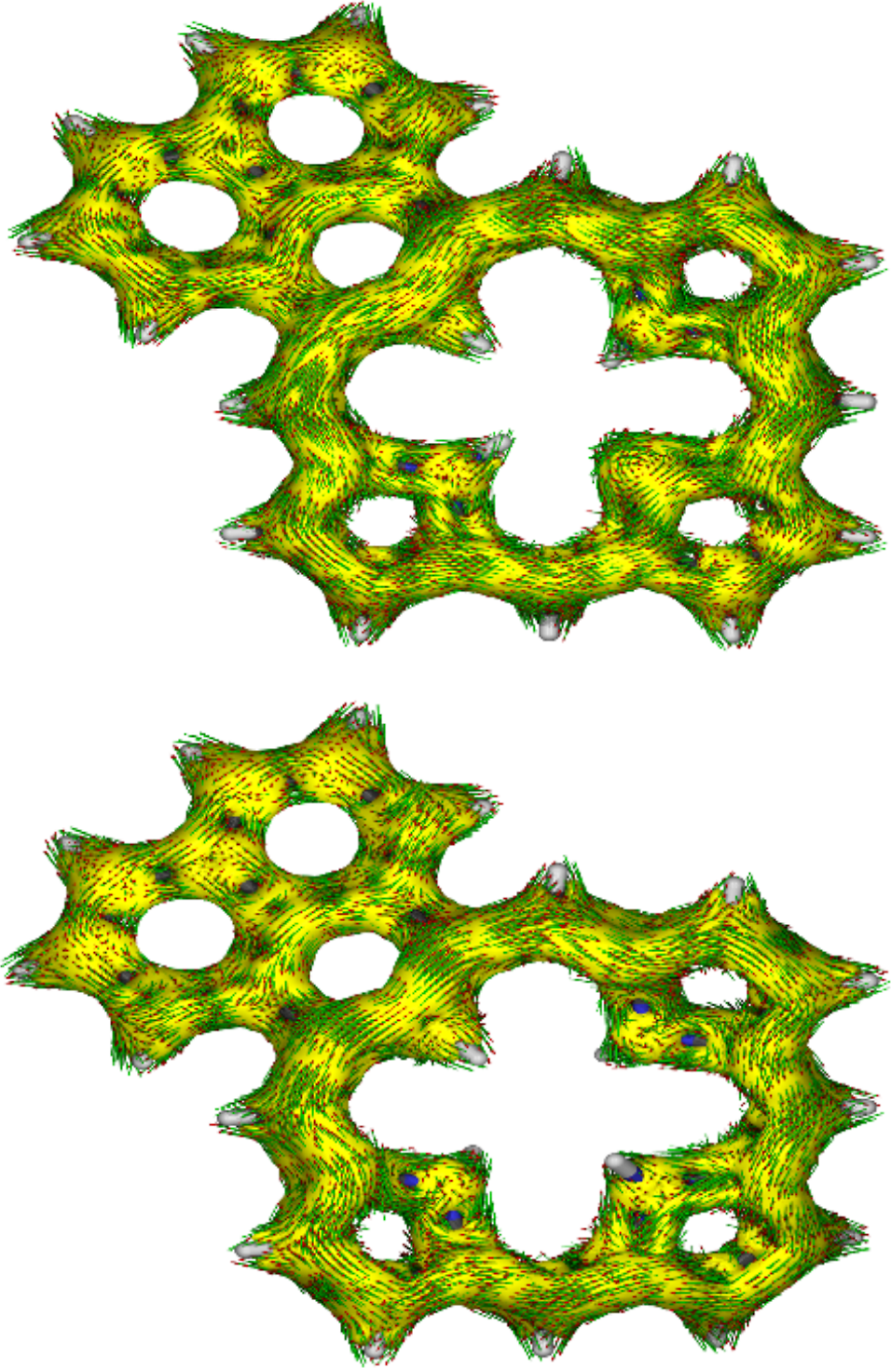
AICD plots (isovalues = 0.05) of phenaliporphyrin tautomer **PhP**-**23**,**25-H** (top) and monocation **PhPaH** ^+^ (bottom).

Three potential monoprotonated phenaliporphyrins were examined, including two with internal methylene units. Tautomer **PhPH** ^+^ proved to be by far and away the most stable and was the only one to exhibit global aromatic character with a NICS­(1_*zz*_) value of −25.39 ppm. Apart from ring a, the individual rings all showed strongly negative NICS_*zz*_ values, and this implies that a 19-atom 18π-electron circuit is favored for this species (Figure 7). The AICD plot for **PhPH** ^+^ confirms this conjecture as it shows electron-flow around the periphery of all three of the pyrrolic subunits (Figure 6). Furthermore, this interpretation is also consistent with the calculated bond lengths for **PhPH** ^+^ (Figure S50). Three dicationic species were examined (Table 3) but none of these show aromatic characteristics. Protonation is favored at the *meso*-positions over the internal carbon atom. The nature of these structures is consistent with the observed instability of diprotonated phenaliporphyrin.

**Figure 7:**
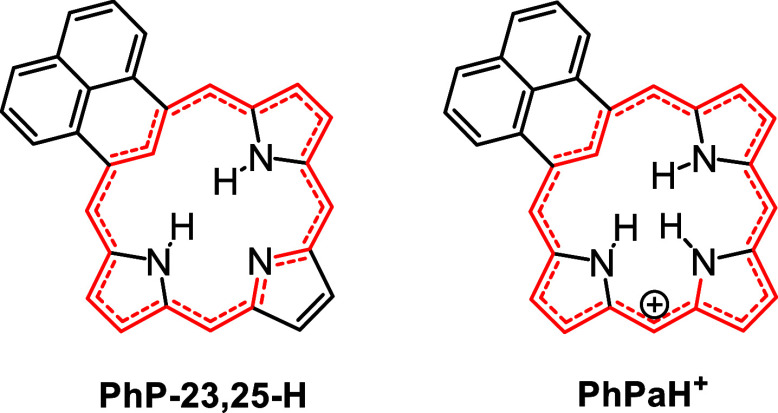
Favored aromatic circuits in phenaliporphyrin and the related monocation.

## Conclusions

Phenaliporphyrin, a new carbaporphyrinoid system incorporating a phenalene subunit, has been prepared from an acenaphthene-fused cyclopropane dialdehyde. The proton NMR spectrum shows that the *meso*-protons are strongly deshielded by a diamagnetic ring current, while the inner C–H resonance appears upfield at ca. −6.5 ppm, and the aromatic character of the macrocycle was confirmed by NICS calculations and AICD plots. The UV–vis spectrum for phenaliporphyrin was also similar to other aromatic porphyrinoids showing a strong Soret band at 443 nm and four Q bands in the visible region, although the absorption at 601 nm was atypically strong. DFT calculations indicate that the phenalene is twisted, but the X-ray structure for **9** shows that this unit is planar; the differences can be attributed to crystal packing forces. Investigations into the reactivity of the unique phenaliporphyrin system are currently underway. In addition, the application of cyclopropane dialdehydes to the synthesis of porphyrinoid systems represents a new strategy and the method shows promise for future applications.

## Experimental Section

NMR spectra were recorded using a 400 or 500 MHz NMR spectrometer and were run at 302 K unless otherwise indicated. ^1^H NMR values are reported as chemical shifts δ, relative integral, multiplicity (s, singlet; d, doublet; dd, doublet of doublets; t, triplet; q, quartet; m, multiplet; br, broad peak) and coupling constant (*J*). Chemical shifts are reported in parts per million (ppm) relative to CDCl_3_ (^1^H residual CHCl_3_ δ 7.26, ^13^C CDCl_3_ triplet δ 77.23) or DMSO-*d*_6_ (^1^H residual *d*_*5*_-DMSO pentet δ 2.49, ^13^C DMSO-*d*_6_ heptet δ 39.7), and coupling constants were taken directly from the spectra. NMR assignments were made with the aid of ^1^H–^1^H COSY, HSQC, DEPT-135, and NOE difference proton NMR spectroscopy. 2D-NMR experiments were performed using standard software. 2D experiments were performed by using standard software. High-resolution mass spectral data (HR MS) were obtained using positive-mode (ESI^+^) and negative mode (ESI^–^) electrospray ionization on a time-of-flight mass spectrometer. ^1^H and ^13^C­{^1^H} NMR spectra for all new compounds are provided in the Supporting Information.
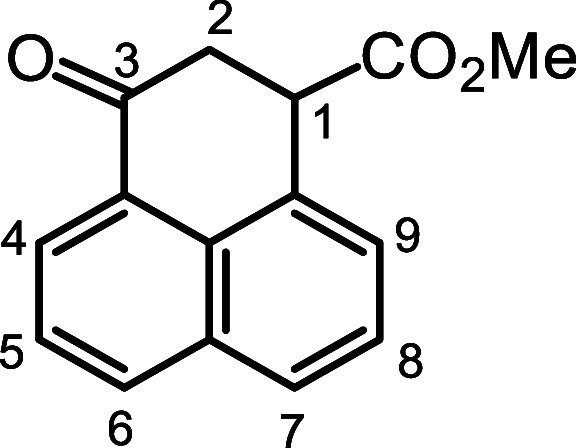


### Methyl 3-Perinaphthanone-1-carboxylate (**16**)

Anhydride **17** ^22,23^ (9.484 g, 0.042 mol) was dissolved in nitromethane (100 mL), AlCl_3_ (22.74 g, 4 equiv) was added, and the resulting mixture stirred at room temperature under anhydrous conditions for 16 h. The nitromethane was removed under reduced pressure, and the residue dissolved in ethyl acetate and added to a mixture of ice (350 g) and conc. hydrochloric acid (300 mL). The mixture was extracted with ether, and the organic layer was separated, washed with water and dried over magnesium sulfate. Evaporation of the solvent gave carboxylic acid **18** ^22,23^ (8.249 g, 36.5 mmol, 87%) as a brown solid and this was used without further purification.

The foregoing carboxylic acid (2.213 g, 9.79 mmol) was dissolved in methanol (8 mL) and toluene (56 mL). A 2.0 M solution of trimethylsilyldiazomethane in diethyl ether (6.5 mL, 13.0 mmol) was added via a syringe and the resulting mixture was stirred at room temperature for 2.5 h. The product was purified by column chromatography on silica gel, eluting with dichloromethane, to give the methyl ester (1.865 g, 7.77 mmol, 79%) as a light brown solid. A sample was recrystallized from ethanol to give white crystals, mp 84.5–85.5 °C. ^1^H NMR (500 MHz, CDCl_3_): δ 8.27 (dd, 1H, *J* = 1,2, 7.2 Hz, 4H), 8.10 (dd, 1H, *J* = 1.1, 8.2 Hz, 6H), 7.88 (1H, dd, *J* = 1.1, 8.2 Hz, 7H), 7.65–7.62 (m, 2H, 5,9H), 7.54 (dd, 1H, *J* = 7.1, 8.1 Hz, 8H), 4.47 (dd, 1H, *J* = 3.3, 6.1 Hz, 1H), 3.62 (s, 3H, OMe), 3.25 (dd, 1H, *J* = 3.3, 15.8 Hz), 3.08 (dd, 1H, *J* = 15.8, 6.2 Hz) (CH_2_). ^13^C­{^1^H} NMR (125 MHz, CDCl_3_): δ 195.8 3 C = O), 173.1 (ester (C = O), 134.3 (6-CH), 133.7, 131.1, 129.7, 129.2, 128.1 (7 CH), 127.3 (9-CH), 126.5 (8-CH), 126.2 (5-CH), 125.8 (4-CH), 52.8 (OMe), 46.2 (1-CH), 40.5 (2-CH_2_). HRMS (ESI^+^) *m*/*z*: [M + H]^+^ calcd for C_15_H_13_O_3_^+^ 241.0859; found, 241.0850.
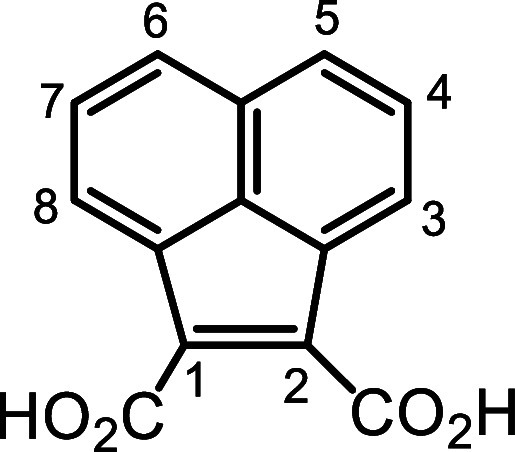


### 1,2-Acenaphthylendicarboxylic Acid (**24**)

A mixture of 1,2-dicyanoacenaphthylene^30,31^ (447 mg), water (14 mL), glacial acetic acid (14 mL), and concentrated sulfuric acid (14 mL) was stirred under reflux. The initial suspension gradually dissolved, and the reaction mixture turned bright red. After 1.5 h, an orange precipitate formed. The reflux was continued for 0.5 h after the appearance of the precipitate and the mixture was then cooled to ca. 50 °C, poured into 57 g of ice, and filtered. The filter cake was dissolved in 5% NaOH (68 mL), washed with chloroform (3 × 70 mL), and filtered. The aqueous solution was acidified to pH ∼ 1 with concentrated HCl, and the resulting precipitate was isolated by suction filtration. Desiccation in vacuo overnight yielded 1,2-acenaphthylenedicarboxylic acid (0.528 g, 2.20 mmol, 99%) as a yellow solid, mp > 260 °C. ^1^H NMR (500 MHz, DMSO-*d*_6_): δ 8.60 (d, 2H, *J* = 7.1 Hz, 3,8H), 8.01 (d, 2H, *J* = 8.1 Hz, 5,6H), 7.66 (dd, 2H, *J* = 7.1, 8.1 Hz, 4,7H). ^13^C­{^1^H} NMR (125 MHz, DMSO-*d*_6_): δ 166.7, 137.9, 137.0, 130.3 (3,8-CH), 129.4 (5,6-CH), 128.0 (4,7-CH), 127.9, 126.9. HRMS (ESI^–^) *m*/*z*: [M – H]^−^ calcd for C_14_H_7_O_4_^–^ 239.0350; found, 239.0345.
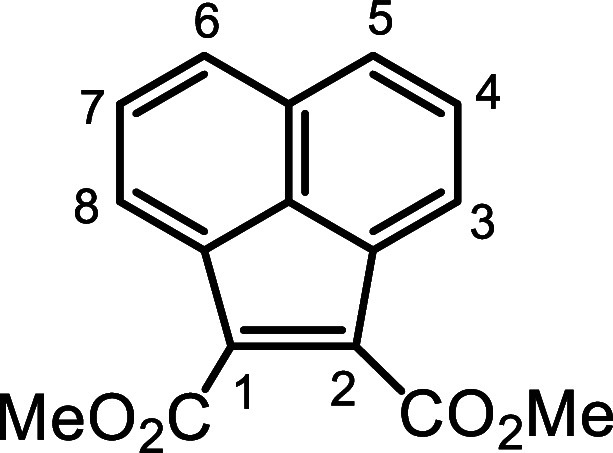


### Dimethyl 1,2-Acenaphthenedicarboxylate (**25**)

1,2-Acenaphthylendicarboxylic acid (318 mg, 1.32 mmol) and methanol (10 mL) were added to a 25 mL round-bottom flask, followed by 16 drops of concentrated sulfuric acid. The mixture was refluxed for 1 h, then cooled. The solvent was removed under reduced pressure, yielding a yellow solid which was then dissolved in cold chloroform (100 mL). The organic solution was washed with ice-cold aqueous NaHCO_3_ (3 × 50 mL) and then dried over magnesium sulfate. The drying agent was suction filtered, and the filtrate concentrated under reduced pressure. The residue was recrystallized from methanol–water to give dimethyl 1,2-acenaphthenedicarboxylate (271 mg, 1.01 mmol, 76%) as golden-yellow crystals, mp 103.4–104.1 °C. ^1^H NMR (500 MHz, CDCl_3_): δ 8.13 (d, 2H, *J* = 7.1 Hz, 3,8H), 7.97 (d, 2H, *J* = 8.1 Hz, 5,6H), 7.64 (dd, 2H, *J* = 7.1, 8.1 Hz, 4,7H), 4.01 (s, 6H, 2 × OMe). ^13^C­{^1^H} NMR (125 MHz, CDCl_3_): δ 165.3, 135.3, 134.7, 130.5 (5,6 CH), 128.7, 128.5 (4,7 CH), 128.4 (3,8H), 127.2, 52. Five (2 × OMe). HRMS (ESI^+^) *m*/*z*: [M + H]^+^ calcd for C_16_H_13_O_4_^+^ 269.0808; found, 269.0806.
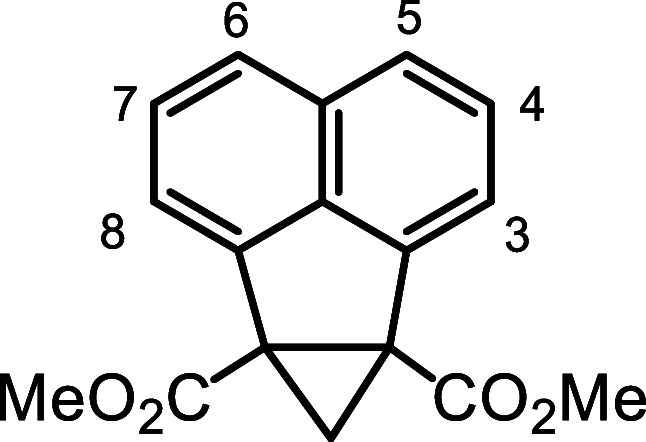


### Dimethyl 1,2-Methanoacenaphthene-1.2-dicarboxylate (**26**)

Dimethyl 1,2-acenaphthenedicarboxylate (100 mg, 0.373 mmol) was added to DMSO (15 mL) in a 100 mL round-bottom flask, followed by the addition of 1.5 equiv trimethyl sulfoxonium iodide (123 mg) and 1.5 equiv sodium hydride (22.4 mg, 60% in mineral oil). The reaction was stirred for 2 h at room temperature under a nitrogen atmosphere and the resulting mixture was transferred to a separating funnel and was extracted with dichloromethane. The solution was acidified with 5 drops of concentrated hydrochloric acid to facilitate the extraction of the organic product. The organic layer was then washed with brine to remove residual DMSO and further washed with water. The organic layer was dried over Na_2_SO_4,_ and the solvent removed under reduced pressure to yield **26** (105 mg, 0.372 mmol, quantitative) as a reddish crystalline solid, mp 79.7–80.8 °C. ^1^H NMR (500 MHz, CDCl_3_): δ 7.69 (dd, 2H, *J* = 0.7, 8.2 Hz, 5.6H), 7.57 (dd, 2H, *J* = 0.7, 7.0 Hz, 3,8H), 7.49 (dd, 2H, *J* = 7.0, 8.2 Hz, 4,7H), 3.85 (s, 6H, 2 × OMe), 3.01 (d, 1H, *J* = 4.6 Hz), 1.53 (d, 1H, *J* = 4.6 Hz) (CH_2_). ^13^C­{^1^H} NMR (125 MHz, CDCl_3_): δ 169.1, 140.1, 135.6, 131.9, 127.7 (4,7-CH), 124.7 (5,6-CH), 121.6 (3,8-CH), 52.9 (2 × OMe), 46.6 (1,2 C), 36.9 (CH_2_). HRMS (ESI^+^) *m*/*z*: [M + H]^+^ calcd for C_17_H_15_O_4_^+^ 283.0965; found, 283.0963.
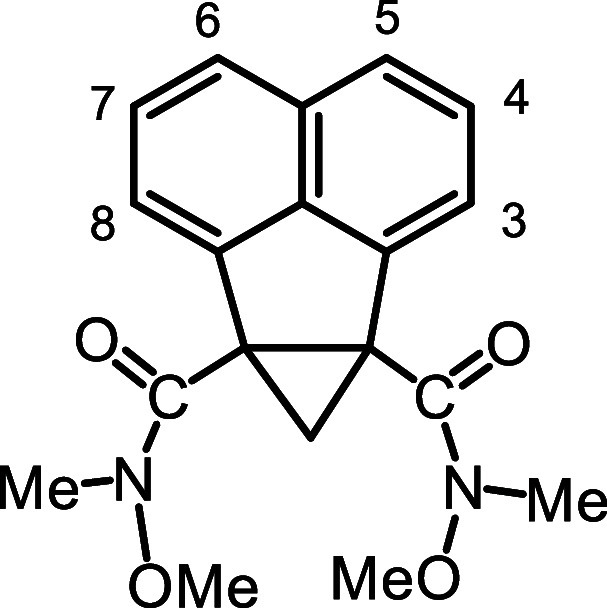


### 1,2-Methanoanenaphthene-1,2-bis­(*N*-methoxy-*N*-methylcarbamide) (**29**)

Dimethyl 1,2-methanoacenaphthene-1,2-dicarboxylate (100 mg, 0.355 mmol) and *N*,*O*-Dimethyl hydroxylamine hydrochloride (53.5 mg, 0.549 mmol) was slurried into 7 mL dry THF and cooled to −10 °C under a nitrogen atmosphere. A solution of isopropyl magnesium chloride (0.53 mL, 2.0 M in THF) was added dropwise to the reaction mixture and the resulting mixture was stirred for 2.5 h and quenched with saturated ammonium chloride solution. The product was extracted with ether and the organic solution was dried over anhydrous sodium sulfate and concentrated in vacuo. Chromatographic purification on silica eluting with ethyl acetate–hexane mixture (3:2 v/v) afforded the bis-amide (105 mg, 0.308 mmol, 87%) as light-yellow crystals, mp 75.6–76.1 °C ^1^H NMR (500 MHz, CDCl_3_): δ 7.65 (dd, 2H, *J* = 1.7, 7.3 Hz), 7.46–7.42 (m, 4H), 3.57 (s, 6H, 2 × OMe), 3.24 (s, 6H, 2 × N–Me), 2.82 (d, 1H, *J* = 4.5 Hz), 1.47 (d, 1H, *J* = 4.5 Hz) (cyclopropane-CH_2_). ^13^C­{^1^H} NMR (125 MHz, CDCl_3_): δ 168.6, 142.3, 135.1, 132.2, 127.6 (4,7-CH), 124.2 (3,8-CH), 120.4 (5,6-CH), 60.8 (2 × OMe), 45.5, 35.0 (CH_2_), 34.6 (2 × NMe). HRMS (ESI^+^) *m*/*z*: [M + H]^+^ calcd for C_19_H_21_N_2_O_4_^+^ 341.1496; found, 341.1497.
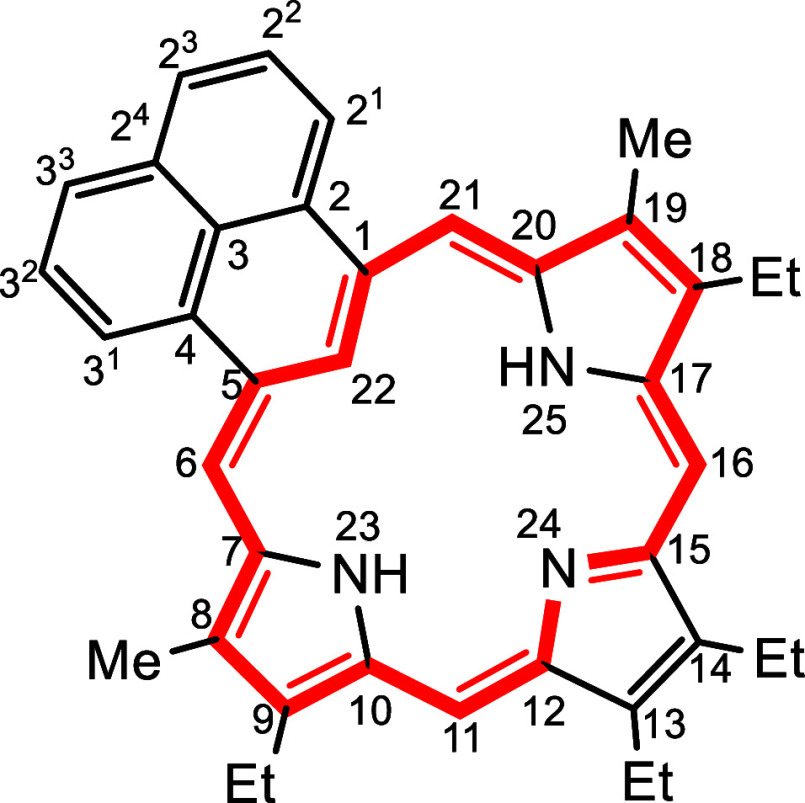


### 9,13,14,18-Tetraethyl-8,19-dimethylphenaliporphyrin (**9**)

Lithium aluminum hydride 11.1 mg, 1 equiv was added to a solution of bis-amide **29** (100 mg, 0.294 mmol) in THF (15 mL) and the resulting mixture stirred at room temperature for 30 min under a nitrogen atmosphere. The reaction was cautiously quenched with several drops of water and extracted with ethyl acetate. The resulting organic layer was dried of anhydrous sodium sulfate and the solvent removed under reduced pressure to give dialdehyde **21** (64.1 mg, 0.288 mmol, 98%) as red crystals. Due to rapid degradation, the crude product was immediately used without further purification.

Tripyrrane dicarboxylic acid **30** (100 mg, 0.220 mmol) was stirred with TFA (1 mL) under nitrogen for 2 min in a pear-shaped flask, followed by the addition of the foregoing dialdehyde (49.0 mg, 0.230 mmol) in dichloromethane (20 mL). The mixture was stirred overnight under a nitrogen atmosphere and then oxidized by shaking the solution with dilute aqueous FeCl_3_ (200 mg in 400 mL H_2_O) for 7 min. The organic layer was washed with a saturated solution of sodium bicarbonate and concentrated under reduced pressure. The residue was purified by column chromatography on grade 3 alumina eluting dichloromethane–hexane (60:40). A dark green fraction was collected and was further purified on silica eluting with dichloromethane. Recrystallization from chloroform-hexane gave phenaliporphyrin (11.5 mg, 0.0209 mmol, 10%) as dark green crystals, mp 252.8–253.5 °C. UV–vis (1% Et_3_N–CH_2_Cl_2_): λ_max_/nm (log ε): 346 (4.40), 418 (4.61), 443 (5.17), 474 (4.76), 601 (4.63), 651 (3.75), 722 (3.63). UV–vis (3 equiv TFA-CH_2_Cl_2_): λ_max_/nm (log ε): 309 (4.52), 353 (4.36), 454 (5.10), 639 (4.45), 656 (4.46), 717 (3.69). ^1^H NMR (500 MHz, CDCl_3_): δ 9.64 (s, 2H, 6,21H), 9.34 (s, 2H, 11,16H), 8.57 (d, 2H, *J* = 7.2 Hz, 2^1^,3^1^H), 7.64 (d, 2H, d, *J* = 7.9 Hz, 2^3^,3^3^H), 7.59 (t, 2H, *J* = 7.5 Hz, 2^2^,3^2^H), 3.89 (q, 4H, *J* = 7.7 Hz), 3.81 (q, 4H, *J* = 7.7 Hz) (4 × CH_2_), 3.43 (s, 6H, 8,19-Me), 1.79 (t, 6H, *J* = 7.7 Hz), 1.74 (t, 6H, *J* = 7.7 Hz) (4 × CH_2_C*H*_3_), −6.48 (s, 1H, 22H). ^13^C­{^1^H} NMR (125 MHz, CDCl_3_): δ 152.1, 143.2, 136.9, 136.1, 135.6, 134.0, 133.2, 133.0, 127.6, 127.4, 127.3 (2^2^,2^3^,3^2^,3^3^-CH), 123.8 (2^1^.3^1^-CH), 114.6 (22-CH), 99.6 (6,21-CH), 95.2 (11,16-CH), 19.9, 19.6 (4 × CH_2_), 18.5, 17.3 (4 × CH_2_ *C*H_3_), 11.8 (8,19-Me). ^1^H NMR (**9**H^+^, 500 MHz, 1 μL TFA-CDCl_3_): δ 9.91 (s, 2H, 6,21H), 9.56 (s, 2H, 11,16H), 8.70 (d, 2H, *J* = 7.2 Hz, 2^1^,3^1^H), 7.82 (d, 2H, d, *J* = 7.9 Hz, 2^3^,3^3^H), 7.77 (t, 2H, *J* = 7.5 Hz, 2^2^,3^2^H), 3.93 (q, 4H, *J* = 7.7 Hz), 3.86 (q, 4H, *J* = 7.7 Hz) (4 × CH_2_), 3.35 (s, 6H, 8,19-Me), 1.79 (t, 6H, *J* = 7.7 Hz), 1.56 (t, 6H, *J* = 7.7 Hz) (4 × CH_2_C*H*_3_), −0.72 (s, 2H, 2 × NH), −3.07 (s, 1H, NH), −5.68 (s, 1H, 22H). HRMS (ESI^+^) *m*/*z*: [M + H]^+^ calcd for C_39_H_40_N_3_^+^ 550.3217; found, 550.3198.

### Crystallographic Analysis of **9**

Clear intense green prism-shaped single crystals of **9** were obtained by room temperature vapor diffusion of hexane into dichloromethane solution. A suitable crystal 0.68 × 0.21 × 0.13 mm^3^ was selected and mounted on a suitable support on an XtaLAB Synergy, Cu micro source, HyPix-Arc 100 diffractometer operating at *T* = 100.00(10) K. The structure was solved with the XT structure solution program^42^ using the Intrinsic Phasing solution method and by using Olex2 as the graphical interface.^43^ The model was refined with version of olex2.refine 1.5-ac7-018 using Gauss–Newton minimization.^44^ CCDC-2502757 contains the supplementary crystallographic data for this structure. These data can be obtained free of charge from the Cambridge Crystallographic Data Centre via https://summary.ccdc.cam.ac.uk/structure-summary?ccdc= 2502757.

### Crystal Data

C_39_H_39_N_3_, *Mr* = 549.764, monoclinic, *P*21/*c* (No. 14), *a* = 9.7527(1) Å, *b* = 21.4909(1) Å, *c* = 14.6356(1) Å, β = 108.665(1)°, α = γ = 90°, *V* = 2906.20(4) Å3, *T* = 100.00(10) K, *Z* = 4, *Z*′ = 1, μ­(Cu Kα)=0.558, 119,949 reflections measured, 6190 unique (*Rint* = 0.0225) which were used in all calculations. The final wR^2^ was 0.0955 (all data) and R1 was 0.0383 (*I* ≥ σ­(*I*)).

### Computational Studies

Calculations on structures **PyP**-**23**,**25-H**, **PyP**-**23**,**24-H**, **PyP**-**22**,**24-H**, and **PyP**-**22**,**23-H**, together with related protonated species, were performed using Gaussian 16, Revision C.01.^45^ Geometry optimization of these structures was performed with the M06-2X functional and the 6-311+ + G­(d,p) basis set.^46^ Vibrational frequencies were computed to confirm the absence of imaginary frequencies to confirm that the structures are minima and to derive zero-point energy and vibrational entropy corrections from unscaled frequencies. Single Point Energy (SPE) calculations were performed on the optimized geometries using the M06-2X functional with a cc-PVTZ basis set.^47^ Two types of NMR calculations were performed; the GIAO method was used to obtain NICS values,^48^ and CGST was used to obtain ACID plots,^41,49^ NICS(0) was calculated at the mean position of all the four heavy atoms in the middle of the macrocycle. NICS­(*a*), NICS­(*b*), NICS­(*c*), NICS­(*d*), NICS­(*e*), and NICS­(*f*) values were obtained by applying the same method to the mean position of the heavy atoms that comprise the individual rings of each macrocycle. In addition, NICS(1)_*zz*_, NICS­(1*a*)_*zz*_, NICS­(1*b*)_*zz*_, NICS­(1*c*)_*zz*_, NICS­(1*d*)_*zz*_, NICS­(1*e*)_*zz*_, and NICS­(1*f*)_*zz*_ were obtained by applying the same method to ghost atoms placed 1 Å above each of the corresponding NICS(0) points and extracting the *zz* contribution of the magnetic tensor. The resulting Cartesian coordinates, energies, 3D geometries, and AICD plots for all the molecules can be found in the Supporting Information.

## Supplementary Material



## Data Availability

The data underlying this study are available in the published article and its online Supporting Information.

## Notes

Selected UV–vis (Figures S1–S6), IR (Figures S7–S9), ^1^H NMR, ^1^H–^1^H COSY, HSQC, DEPT-135, ^13^C­{^1^H} NMR (Figures S10–S38), mass spectra (Figures S39–S44), alternative plots of the X-ray structure for phenaliporphyrin **9** (Figures S45–S48), results from XRD analysis of **9** (Tables S1–S8), selected calculated bond lengths and AICD plots (Figures S49–S60), calculated Gibbs free energies for optimized structures (Table S9), and Cartesian coordinates (Table S10) are provided (PDF)
