# Supplementary material for: Synthesis of Phenaliporphyrin, a PAH-Porphyrin Hybrid, from an Acenaphthene-Fused Cyclopropane Dialdehyde
Source: J Org Chem. 2025 Dec 1;90(49):17422–32. doi: 10.1021/acs.joc.5c02277 (PMC12706790; doi:10.1021/acs.joc.5c02277)
Supplement: Supplementary file 1 [file jo5c02277_si_001.pdf]

Supporting information for
Synthesis of Phenaliporphyrin, a PAH-Porphyrin Hybrid, from an Acenaphthene-fused Cyclopropane Dialdehyde

Sunday Oladapo Jacob,[1] Emily D. Harris,[1] Melissa A. Mathius,[1] Deyaa I. AbuSalim,[1,2,3] Gregory M. Ferrence[1] and Timothy D. Lash[1]*

[1]Department of Chemistry, Illinois State University, Normal, Illinois 61790-4160

[2]Department of Chemistry, Rowan University, Glassboro, New Jersey 08028

[3]STEM Department, Rowan College of South Jersey, Vineland, New Jersey 08360

* E-mail: tdlash@ilstu.edu

Table of Contents

Page

S2-S4	Selected UV-Vis spectra (Figures S1-S6)
S5-S6	Selected IR spectra (Figures S7-S9)
S7-S28	Selected proton, DEPT-135, ^{1}H-^{1}H COSY, HSQC and carbon-13 NMR spectra (Figures S10-S38)
S29-S32	Selected mass spectra (Figures S39-S44)
S33-S35	Alternative views for the X-ray crystal structure of **9** (Figures S45-S48)
S36-S44	Results for the single crystal X-ray diffraction analysis of **9** (Tables S1-S8)
S45-S46	Calculated bond lengths for **PhP-23,25-H** and **PhPaH$^+$** (Figures S49-S50)
S47S56	AICD plots (Figures S51-S60)
S57	Calculated Gibbs free energies for the optimized structures (Table S9)
S58-S66	Cartesian Coordinates (Table S10)

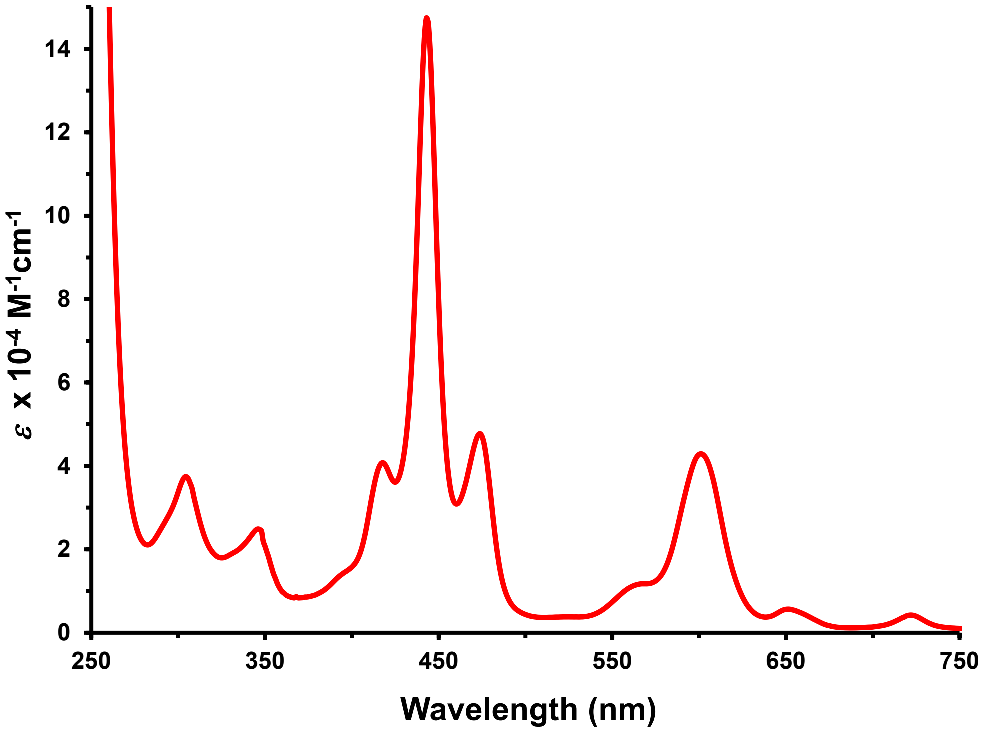

Figure S1. UV-vis spectrum of phenaliporphyrin **9** in 1% Et_3N-CH_2Cl_2.

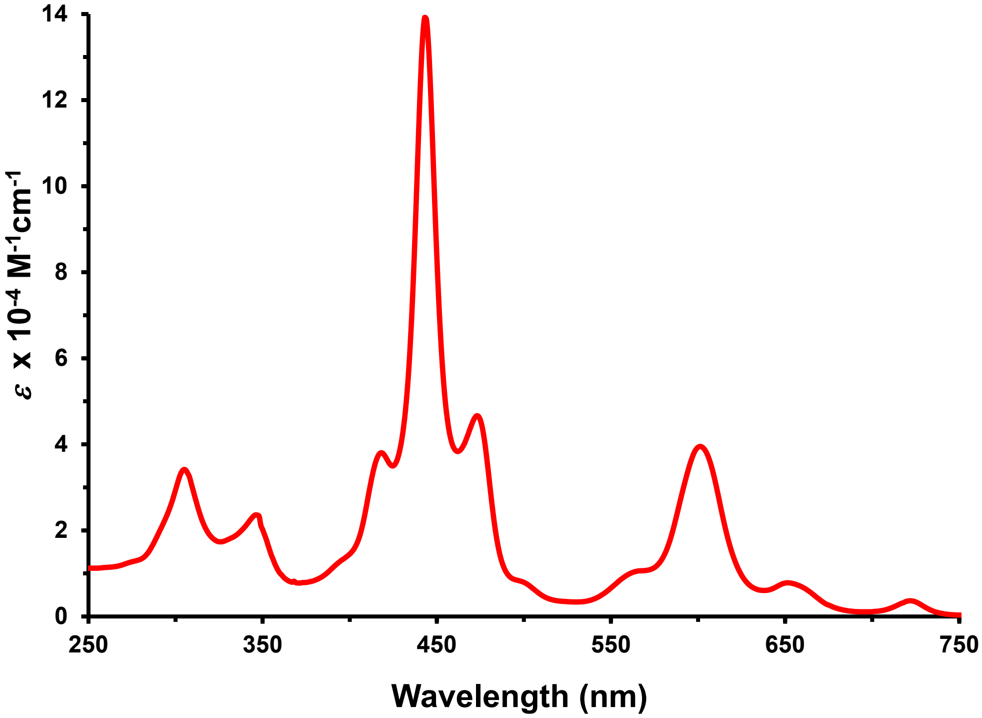

Figure S2. UV-vis spectrum of phenaliporphyrin **9** in CH_2Cl_2.

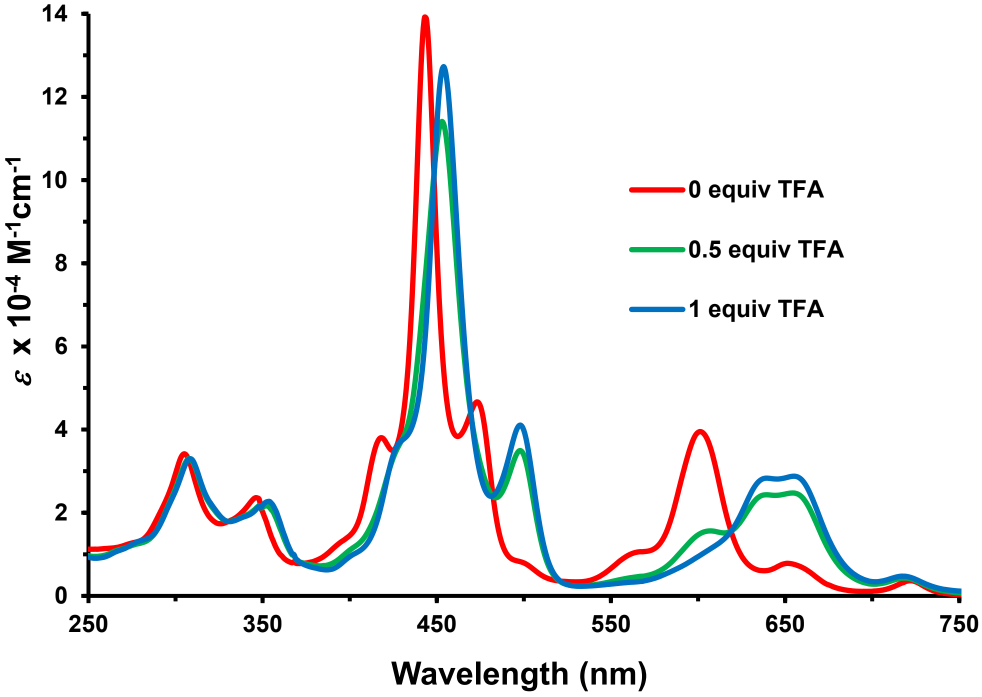

Figure S3. UV-vis spectra of phenaliporphyrin **9** in CH_2Cl_2 with 0, 0.5 and 1 equiv TFA.

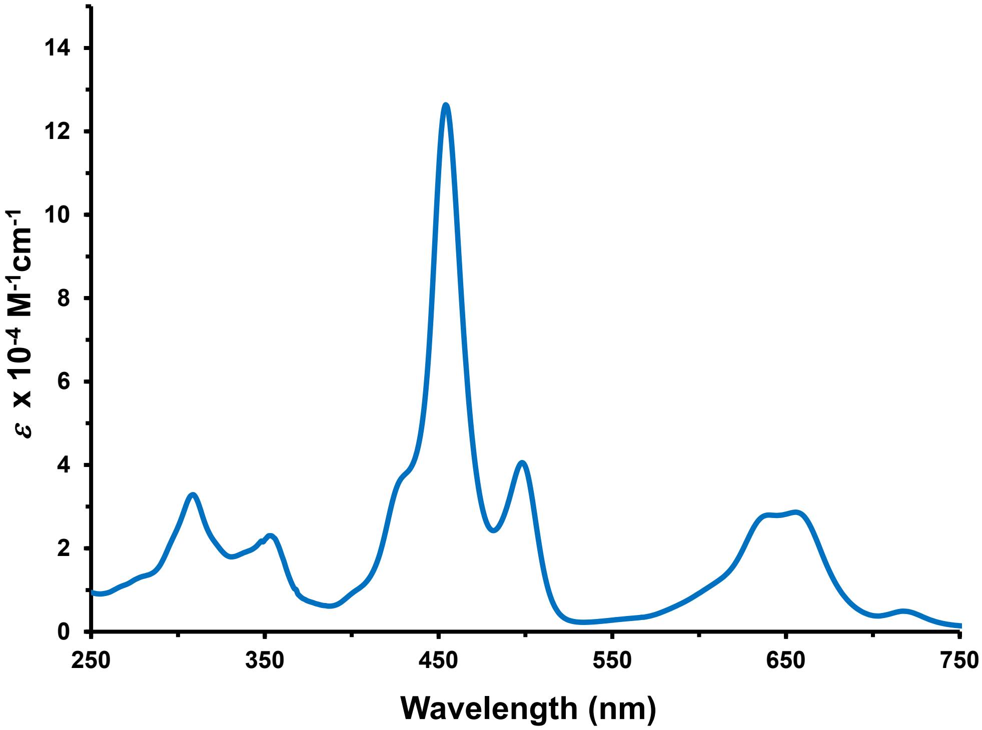

Figure S4. UV-vis spectrum of phenaliporphyrin **9** in 3 equiv. TFA in CH_2Cl_2.

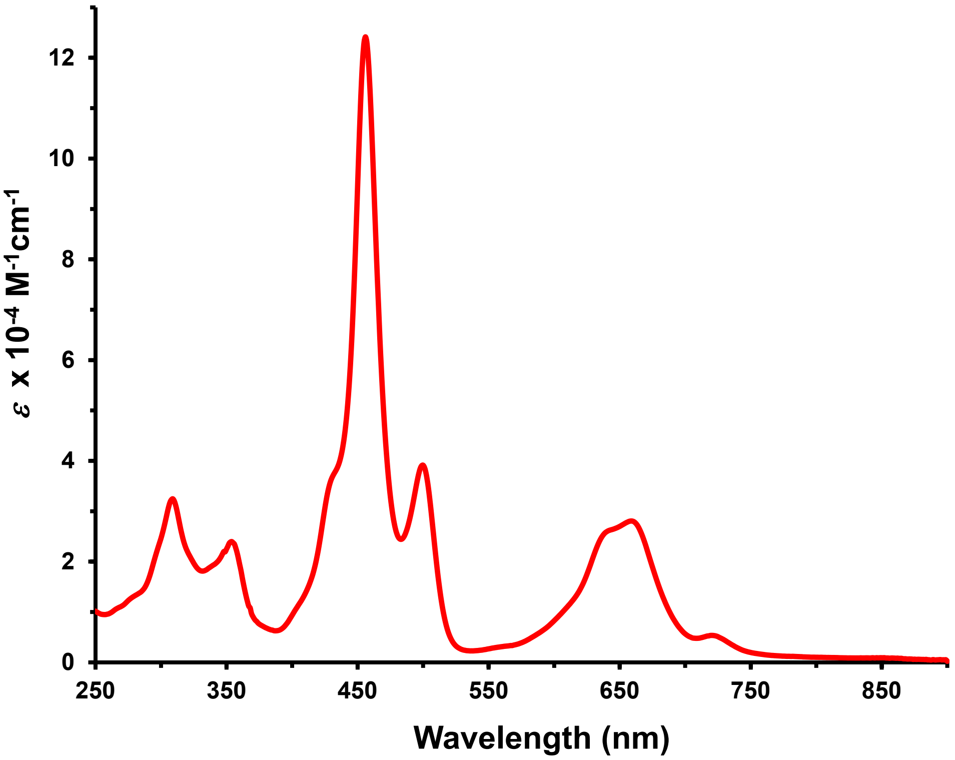

Figure S5. UV-vis spectrum of phenaliporphyrin **9** with 100 equiv. TFA in CH_2Cl_2.

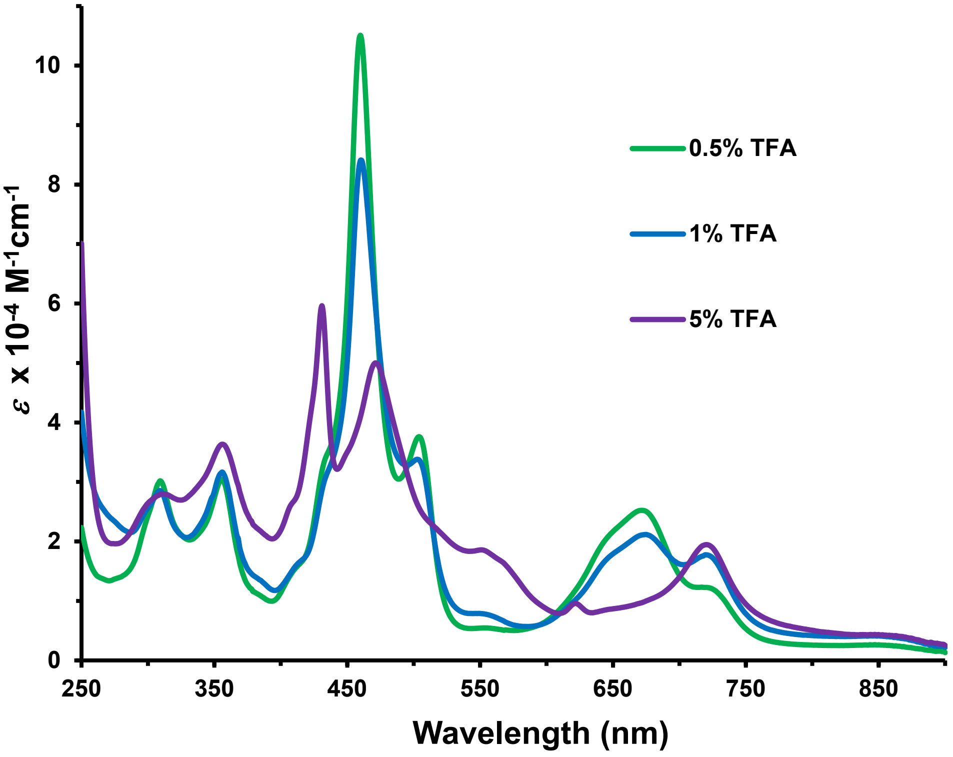

Figure S6. UV-vis spectra of phenaliporphyrin **9** in 0.5%, 1% and 5% TFA in CH_2Cl_2.

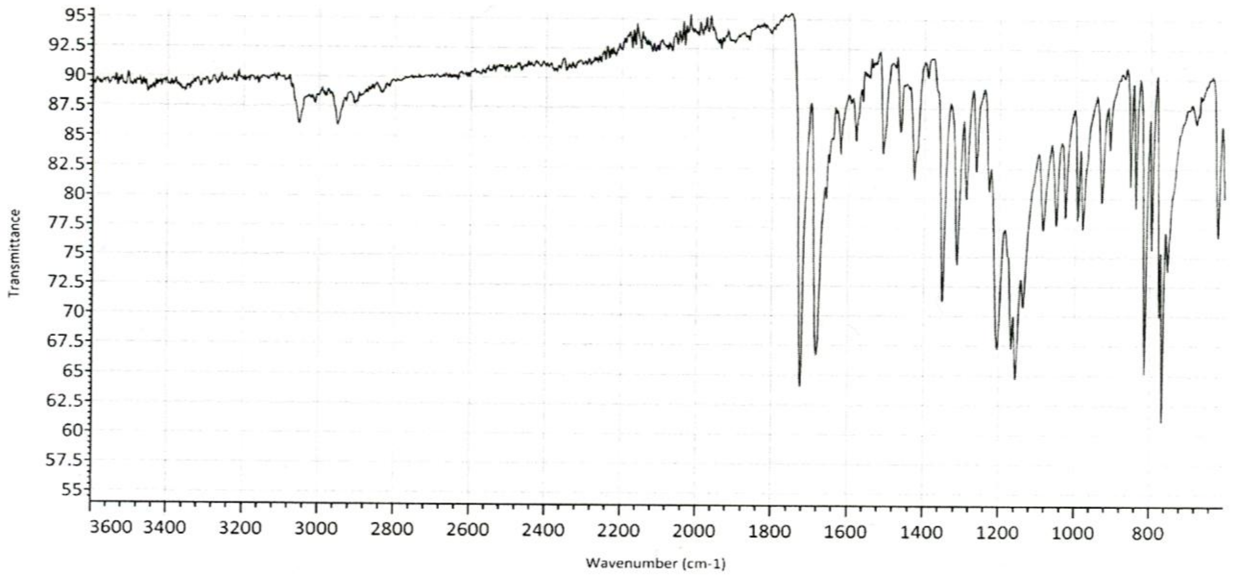

Figure S7. IR spectrum of keto ester **16**.

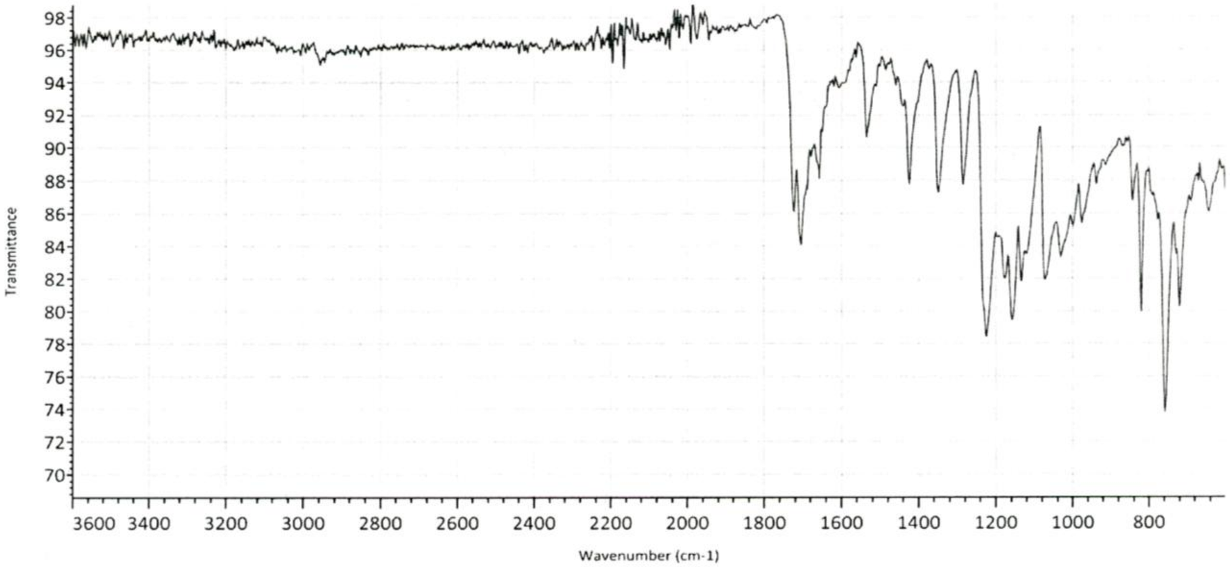

Figure S8. IR spectrum of acenaphthylene diester **25**.

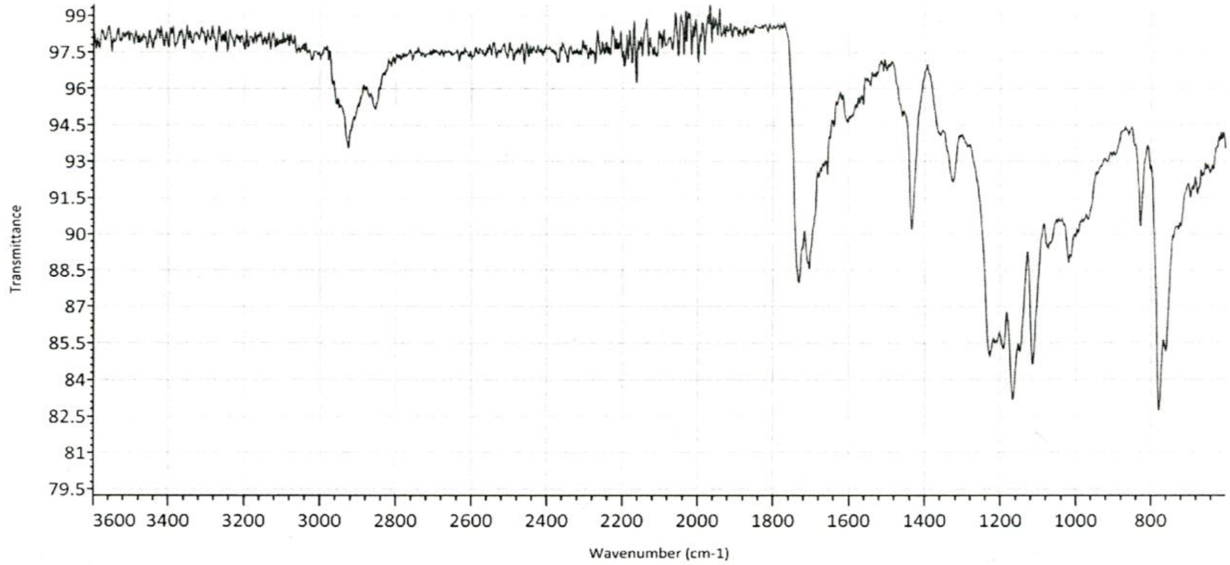

Figure S9. IR spectrum of methanoacenaphthene diester **26**.

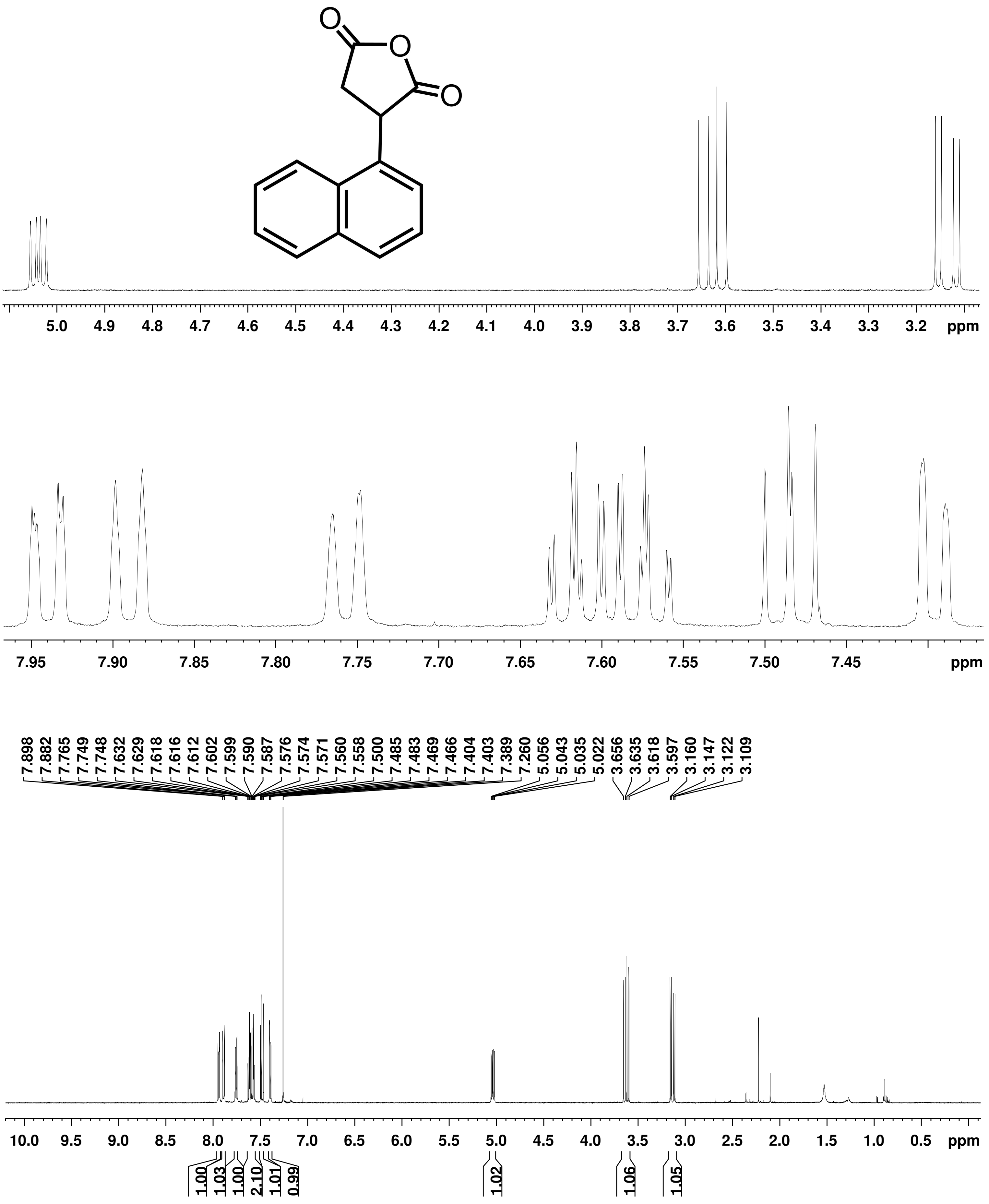

Figure S10. 500 MHz proton NMR spectrum of anhydride **17** in $CDCl_3$.

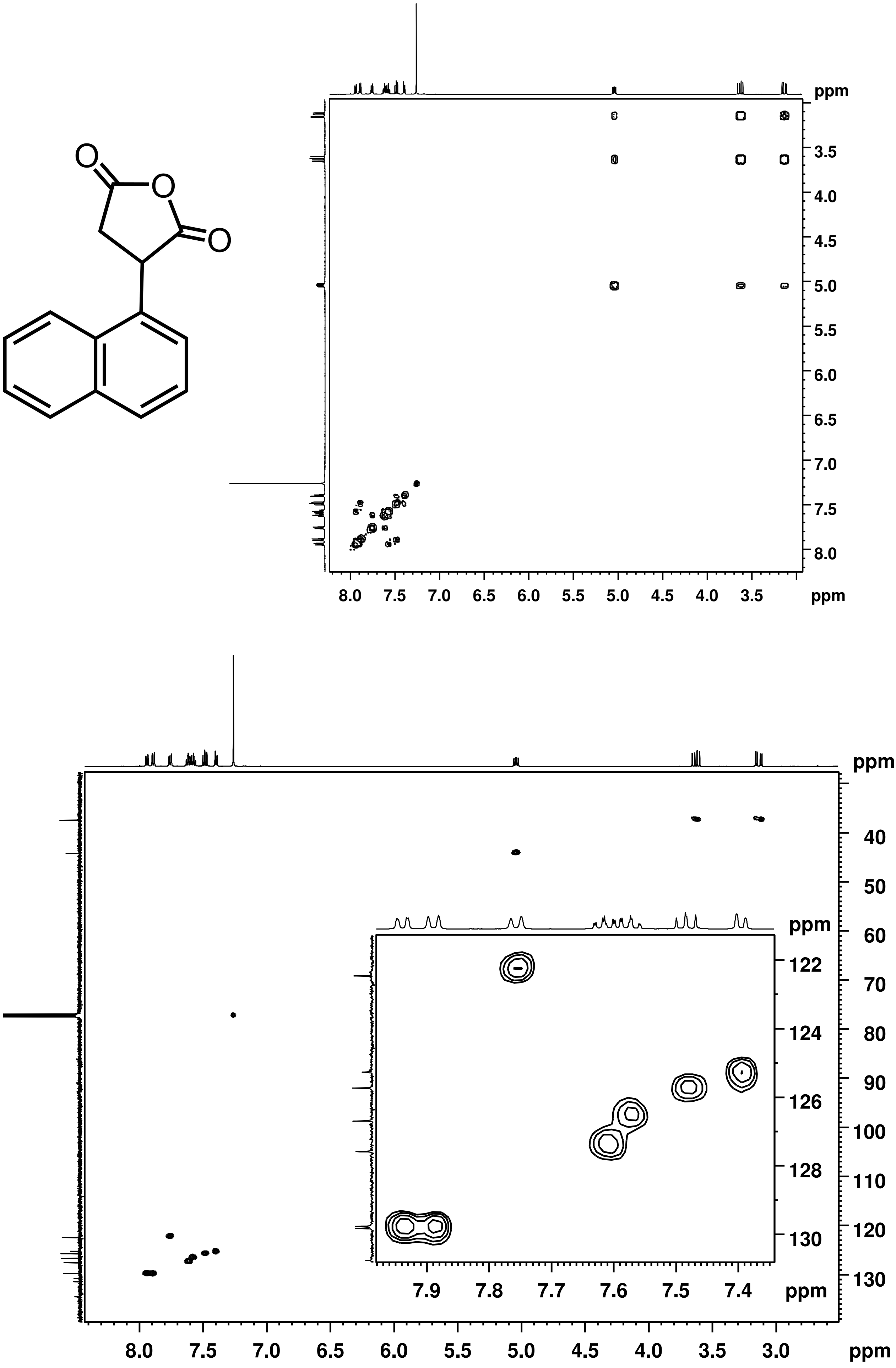

Figure S11. ^{1}H-^{1}H COSY (top) and HSQC (bottom) NMR spectra of anhydride **17** in $CDCl_3$.

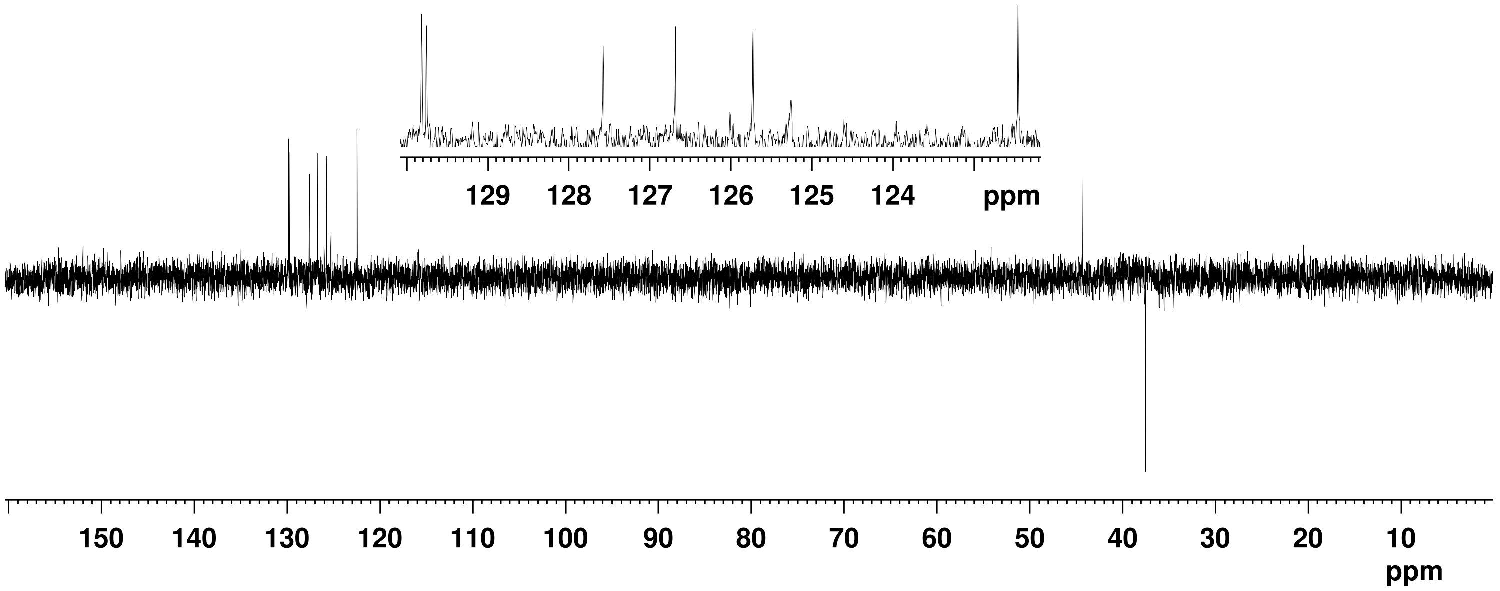

Figure S12. DEPT-135 NMR spectrum of anhydride **17** in $CDCl_3$.

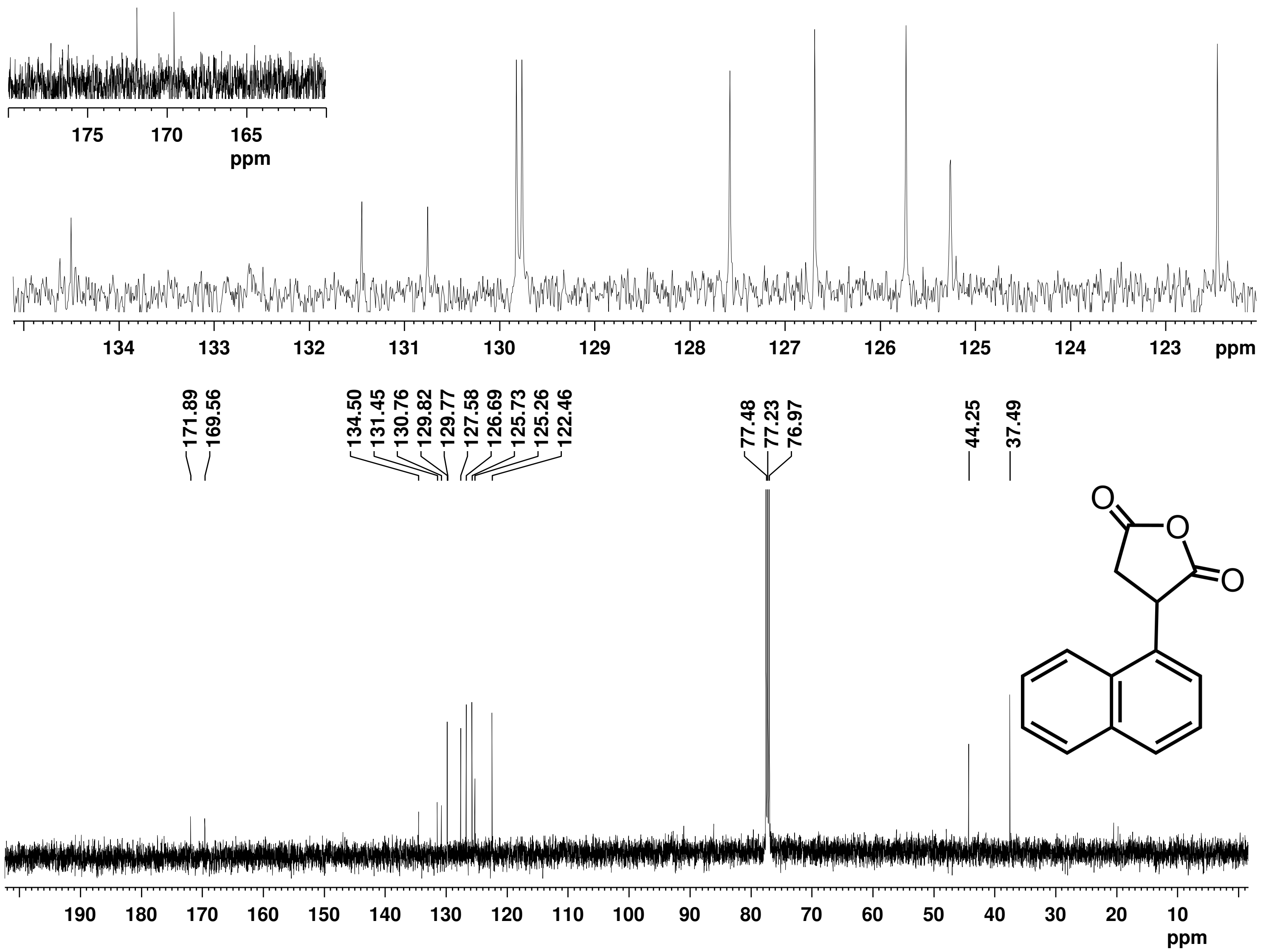

Figure S13. 125 MHz $^{13}C\{^1H\}$ NMR spectrum of anhydride **17** in $CDCl_3$.

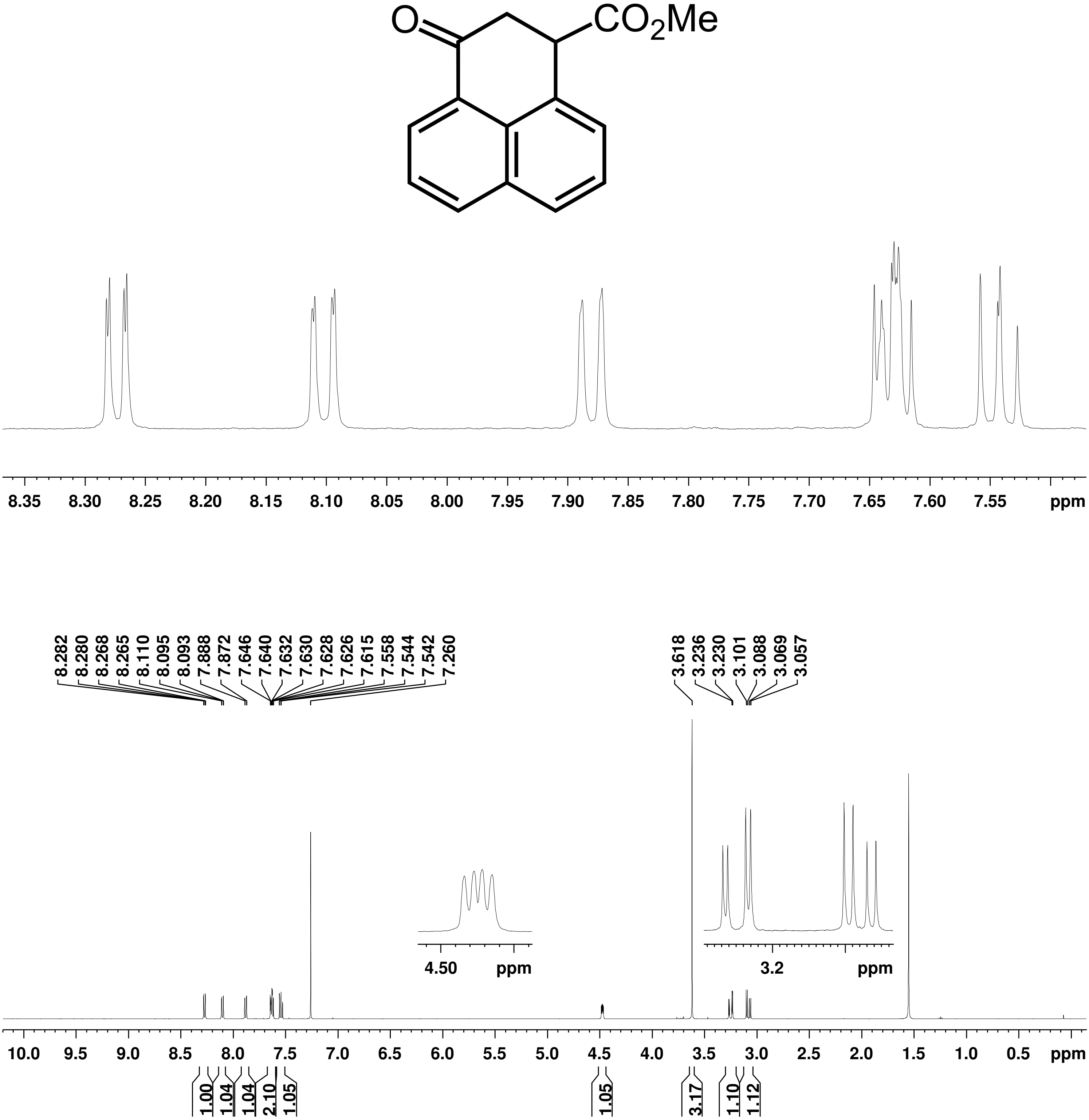

Figure S14. 500 MHz proton NMR spectrum of keto ester **16** in $CDCl_3$.

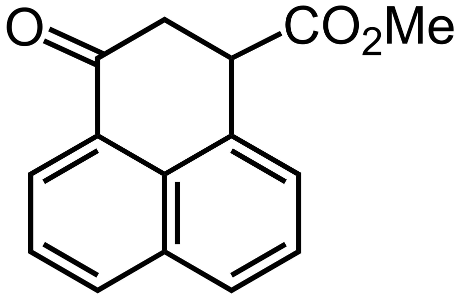

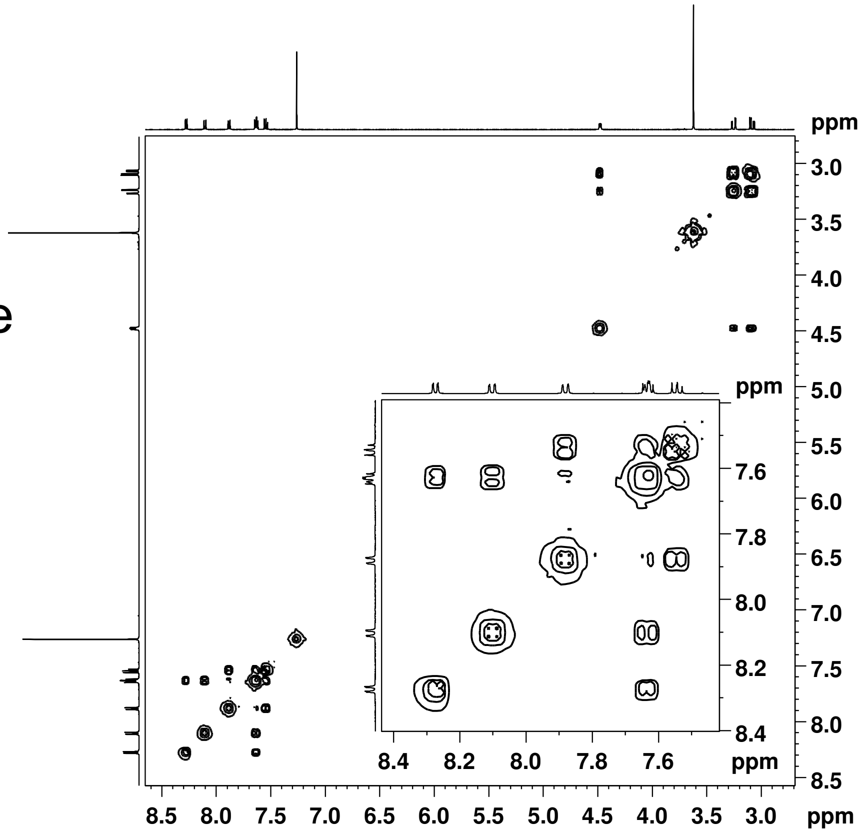

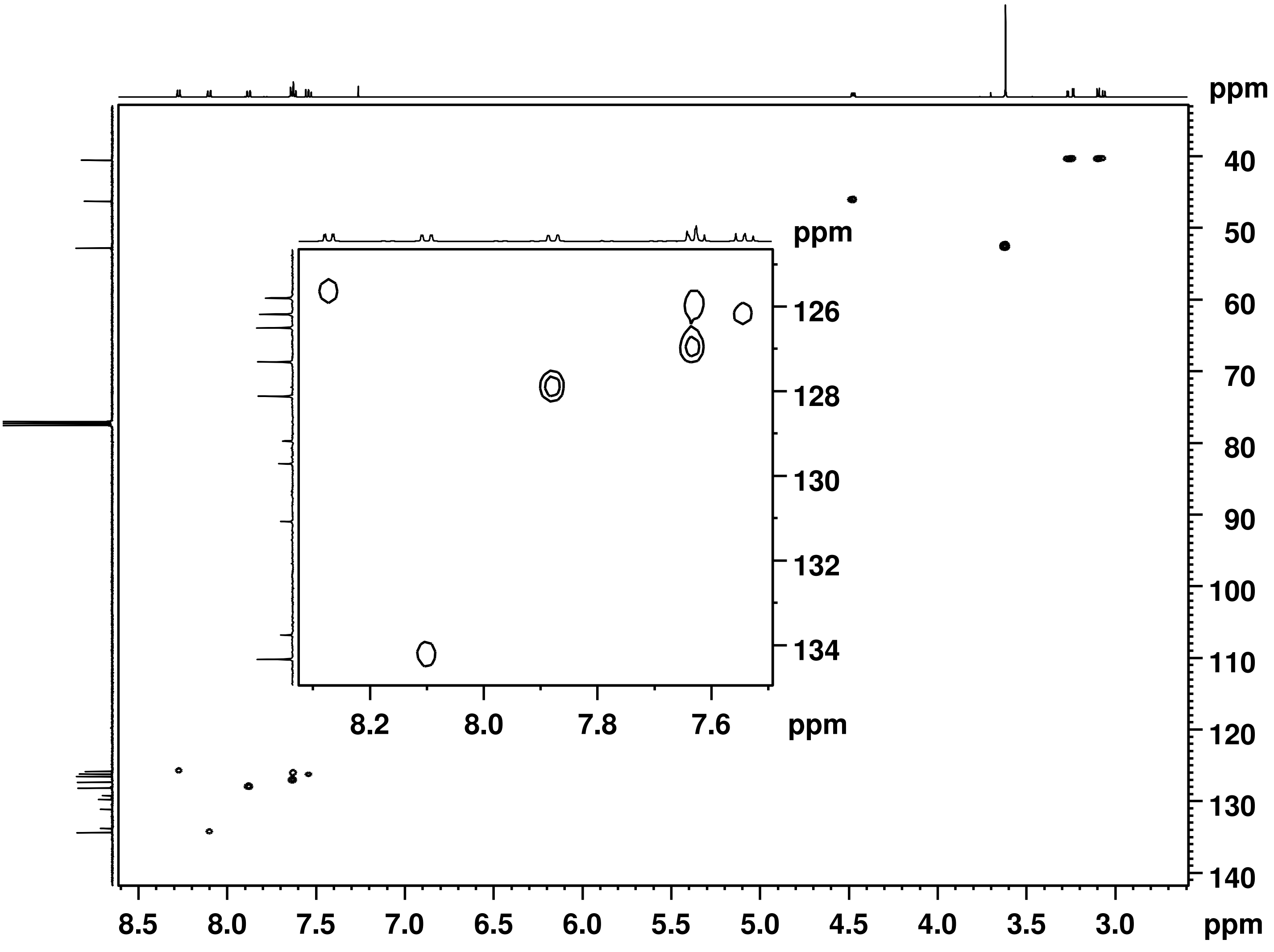

Figure S15. ^{1}H-^{1}H COSY (top) and HSQC (bottom) NMR spectra of keto ester **16** in $CDCl_3$.

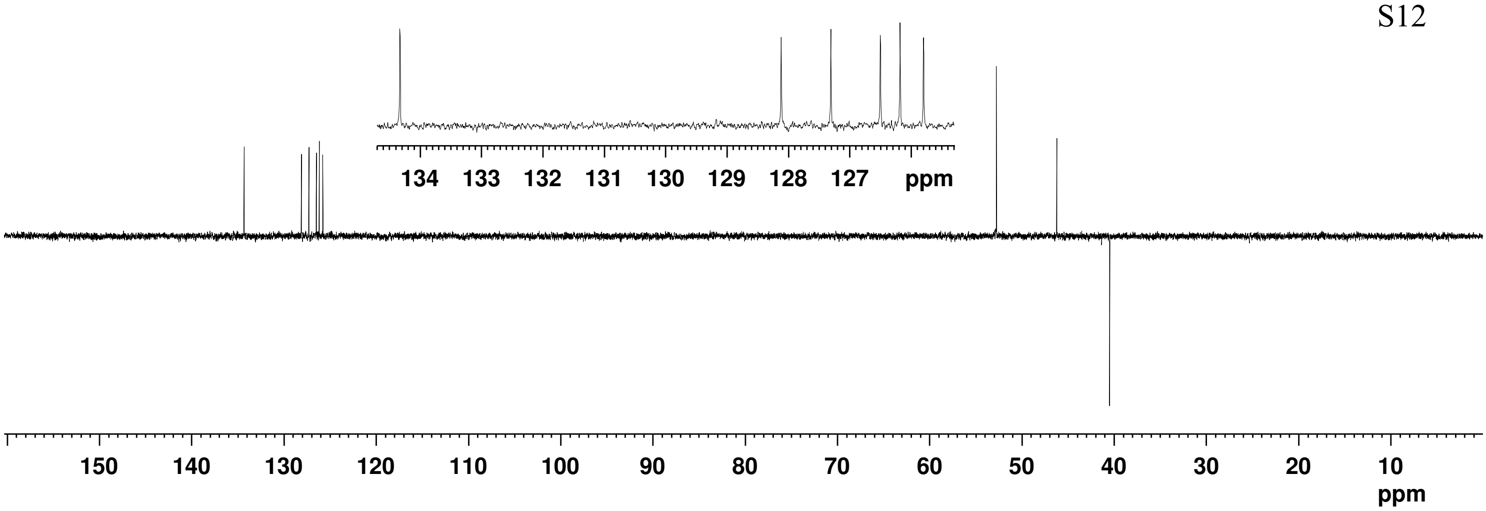

Figure S16. DEPT-135 NMR spectrum of keto ester **16** in $CDCl_3$.

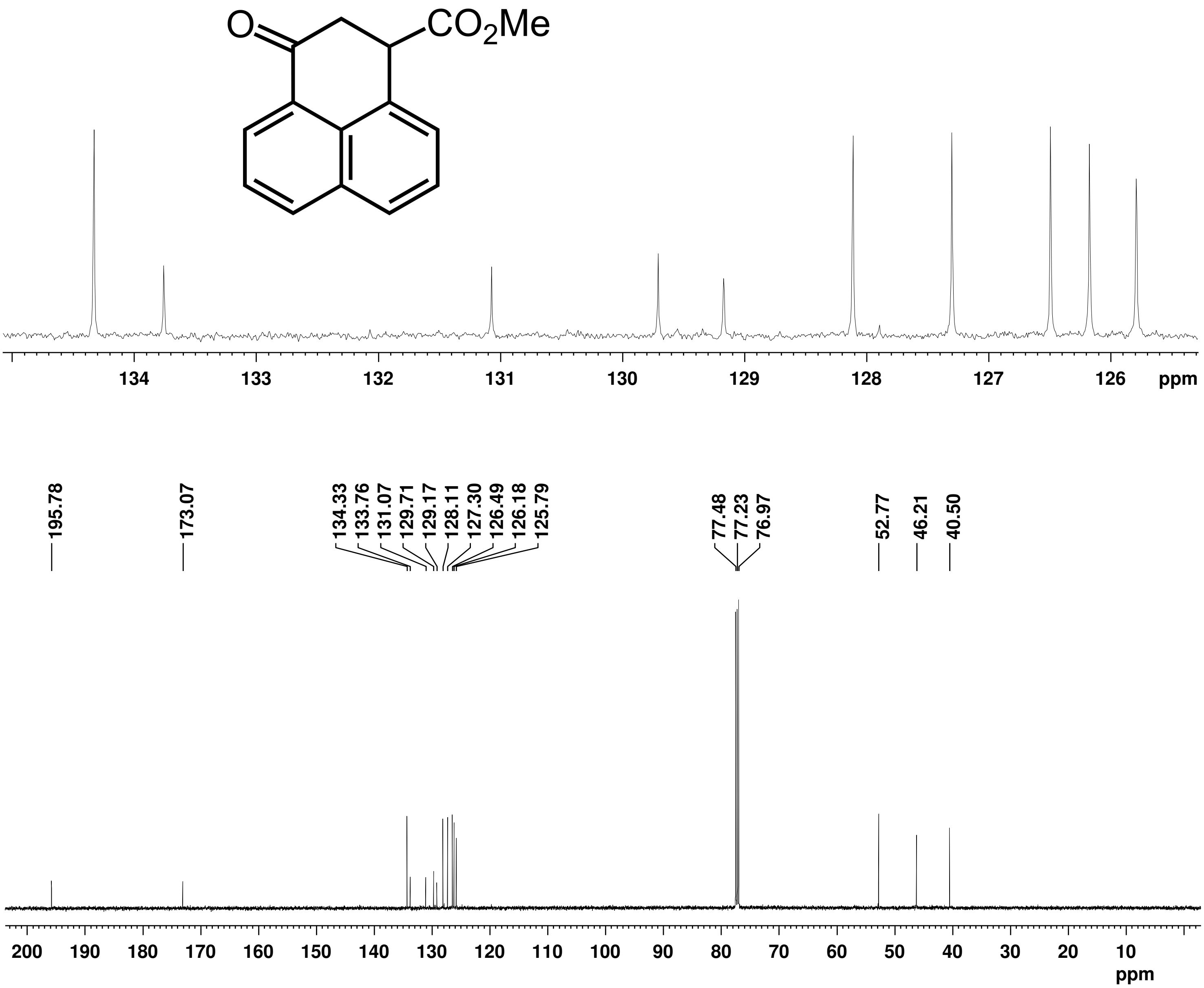

Figure S17. 125 MHz $^{13}C\{^1H\}$ NMR spectrum of keto ester **16** in $CDCl_3$.

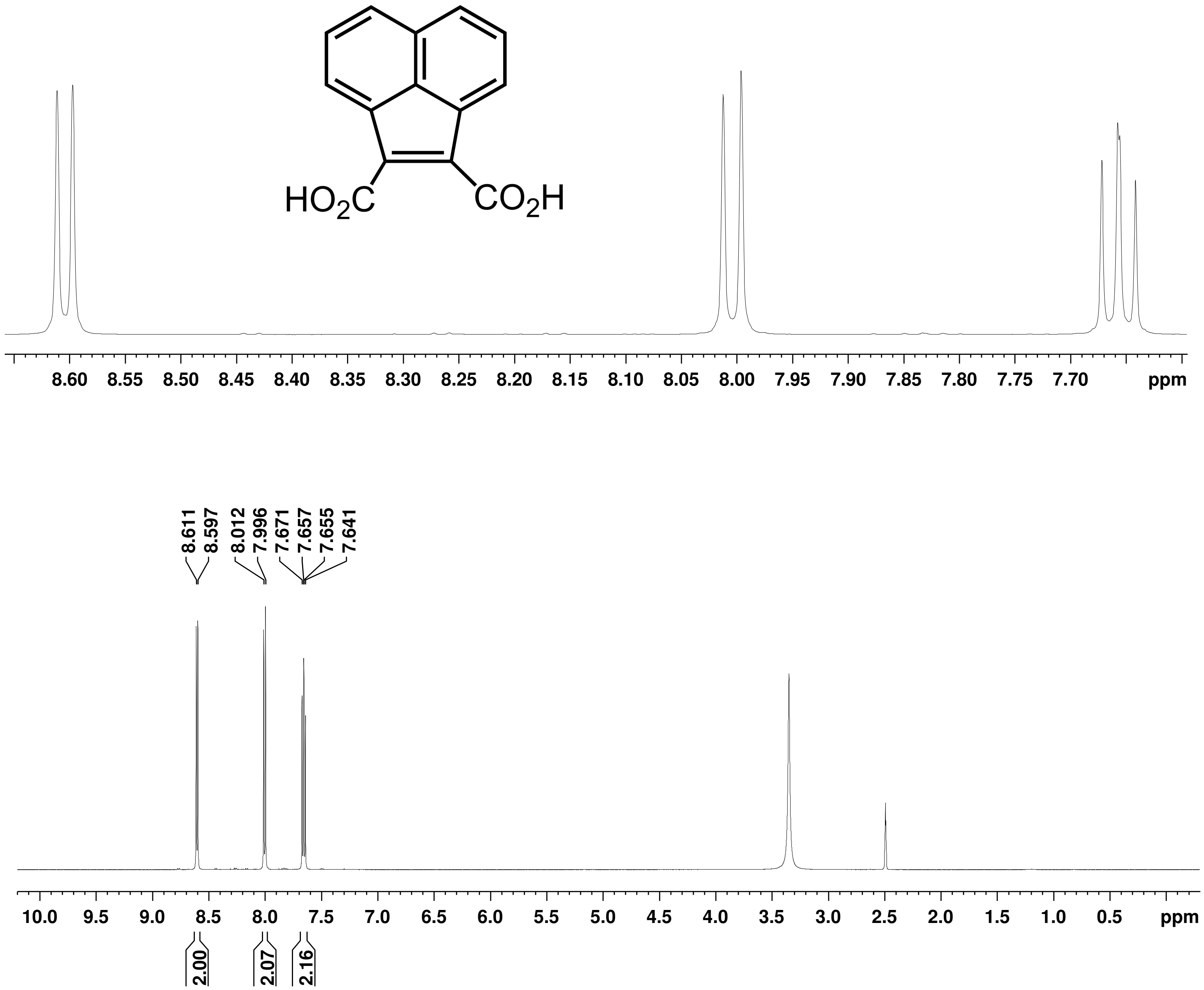

Figure S18. 500 MHz proton NMR spectrum of dicarboxylic acid **24** in DMSO-d_6.

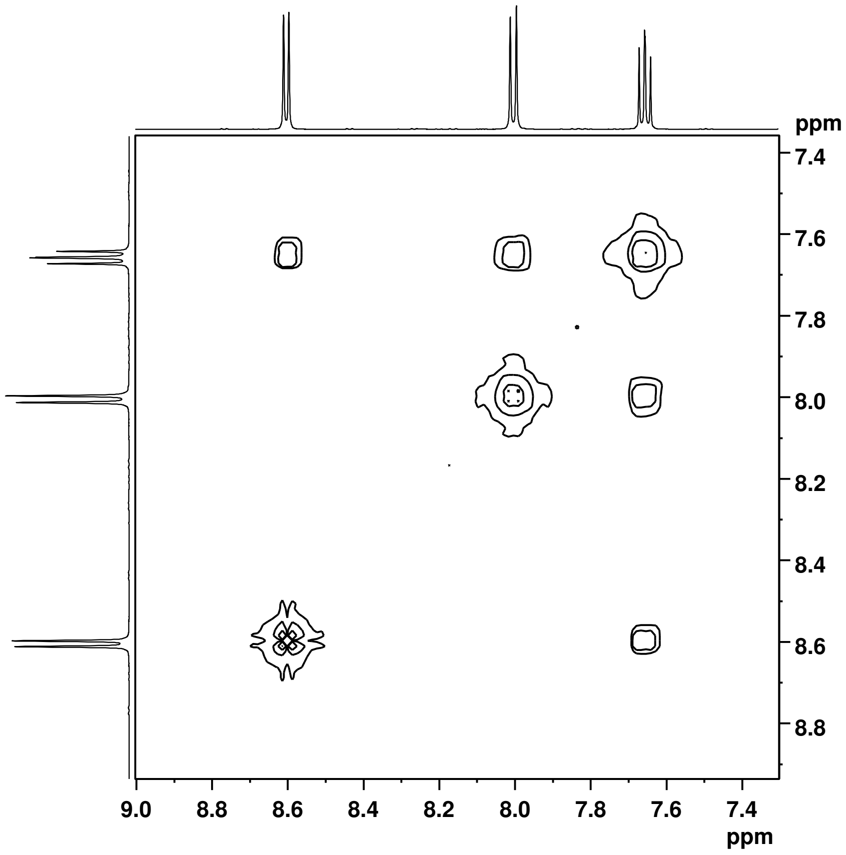

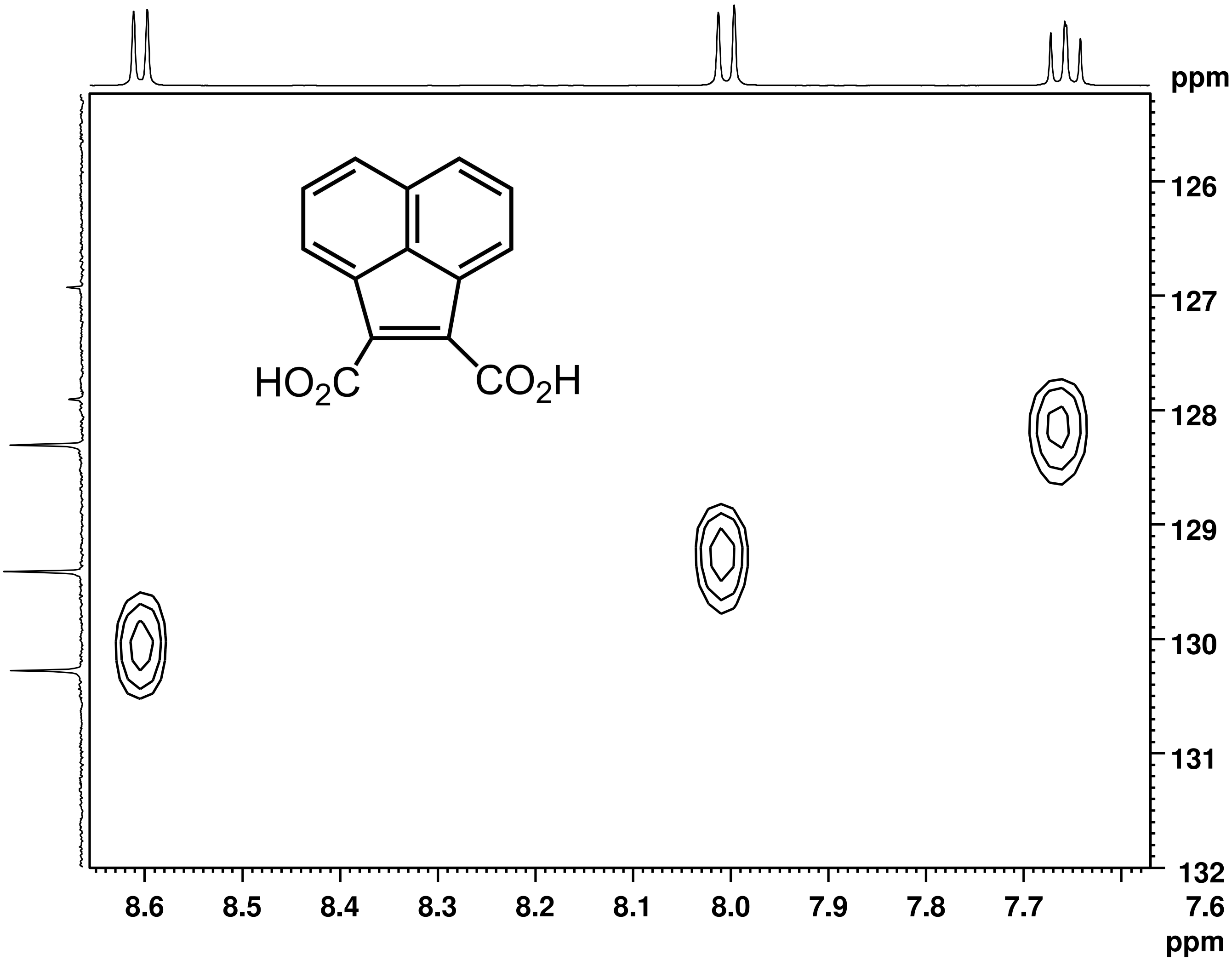

Figure S19. ^{1}H-^{1}H COSY (top) and HSQC (bottom) NMR spectra of dicarboxylic acid **24** in DMSO-d_6.

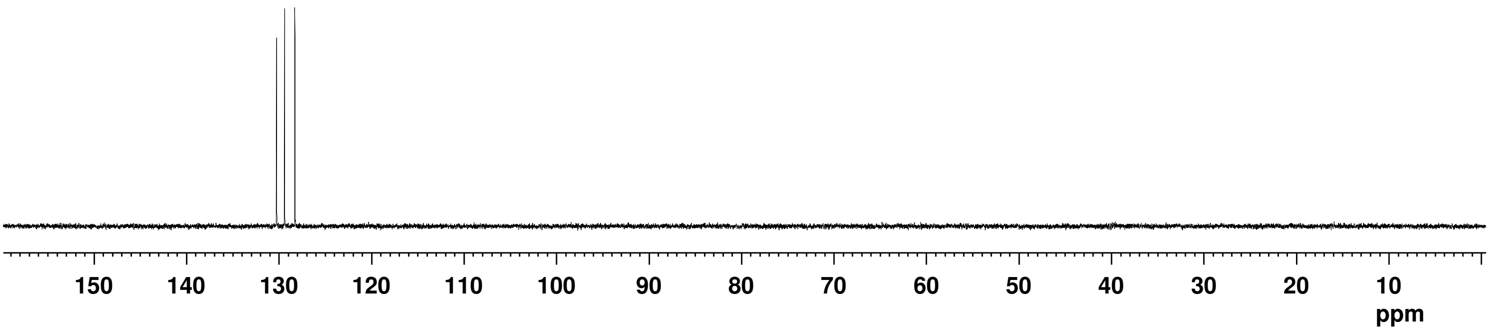

Figure S20. DEPT-135 NMR spectrum of dicarboxylic acid **24** in DMSO-d_6.

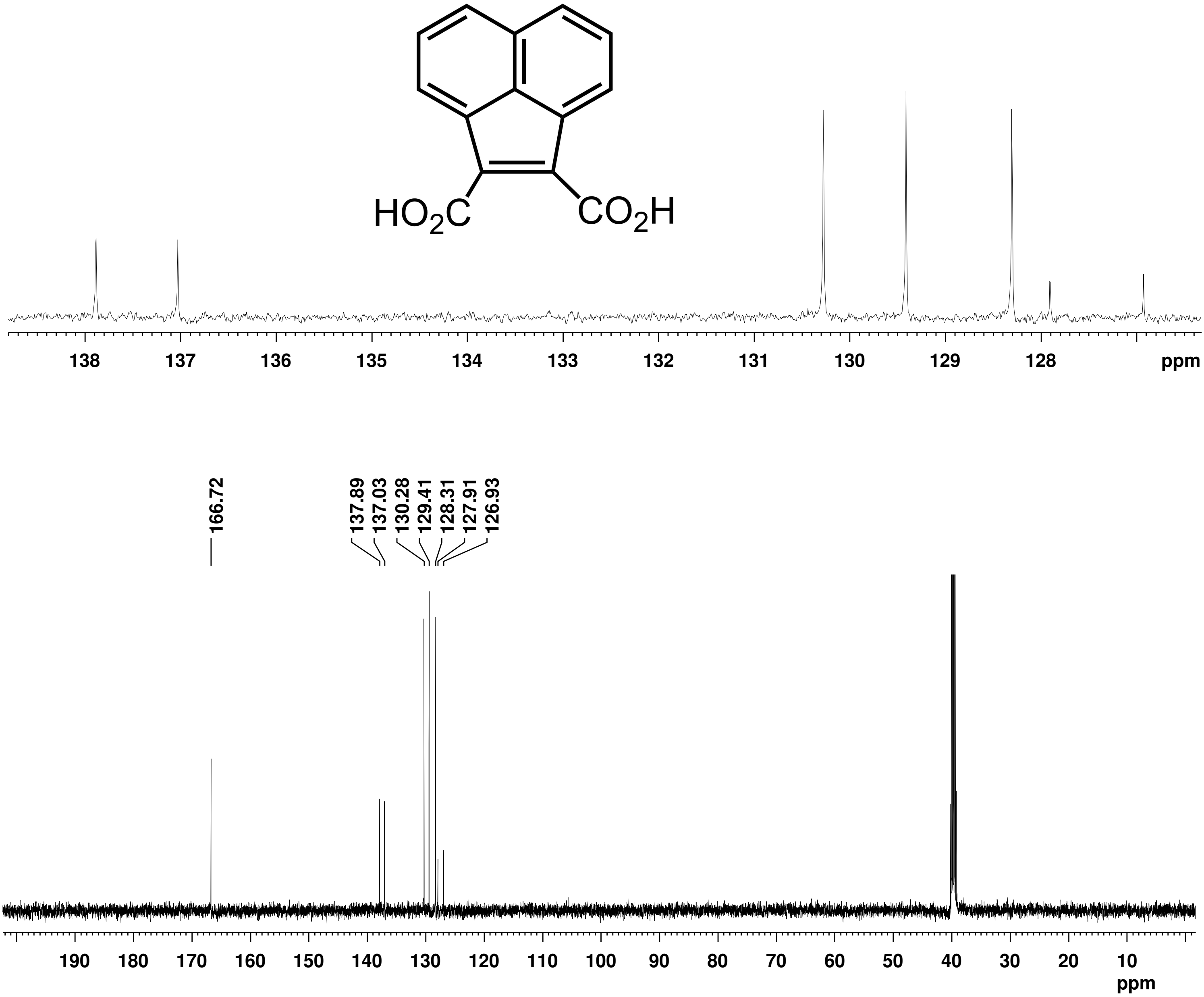

Figure S21. 125 MHz $^{13}C\{^{1}H\}$ NMR spectrum of dicarboxylic acid **24** in DMSO-d_6.

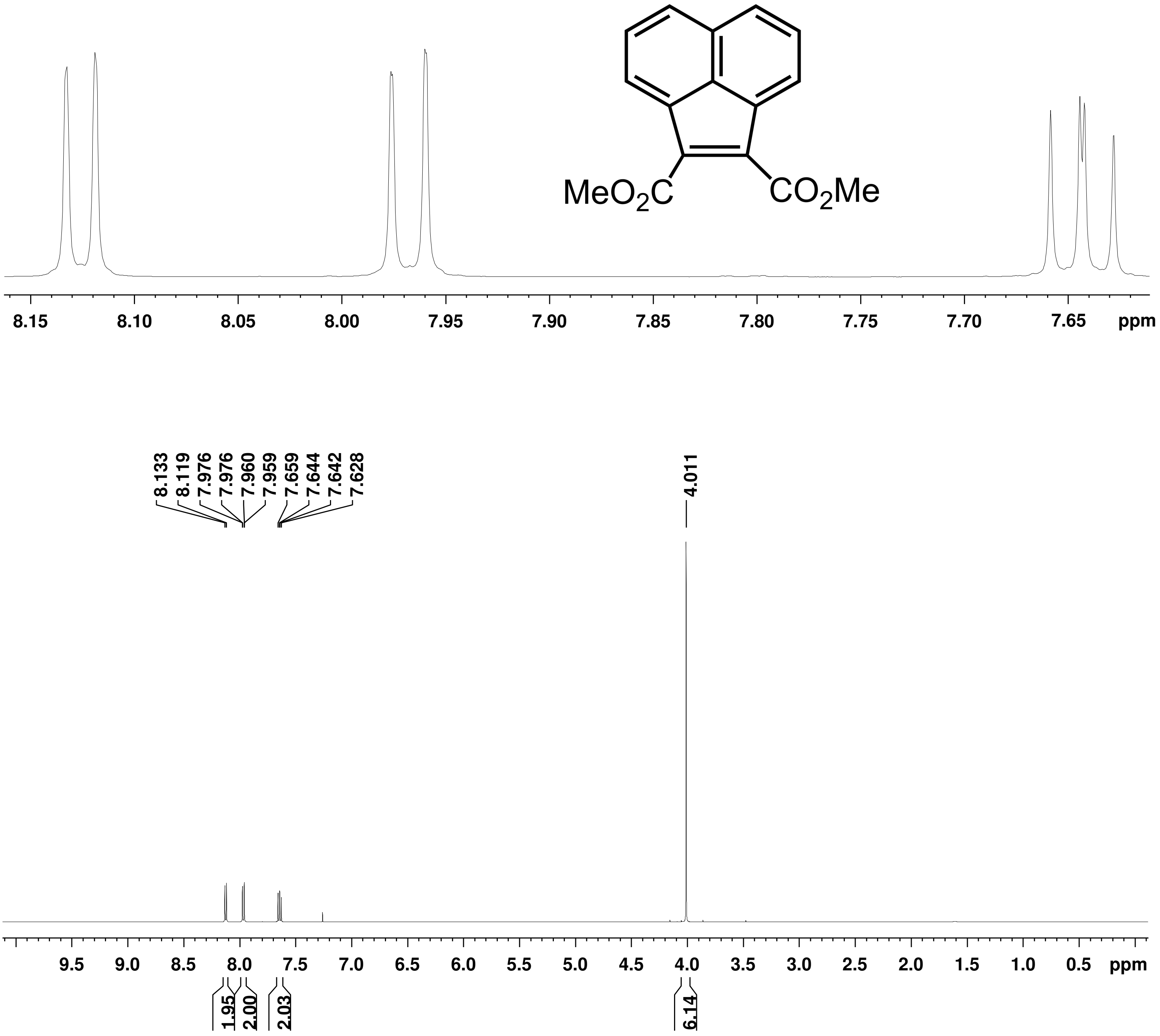

Figure S22. 500 MHz proton NMR spectrum of dimethyl ester **25** in $CDCl_3$.

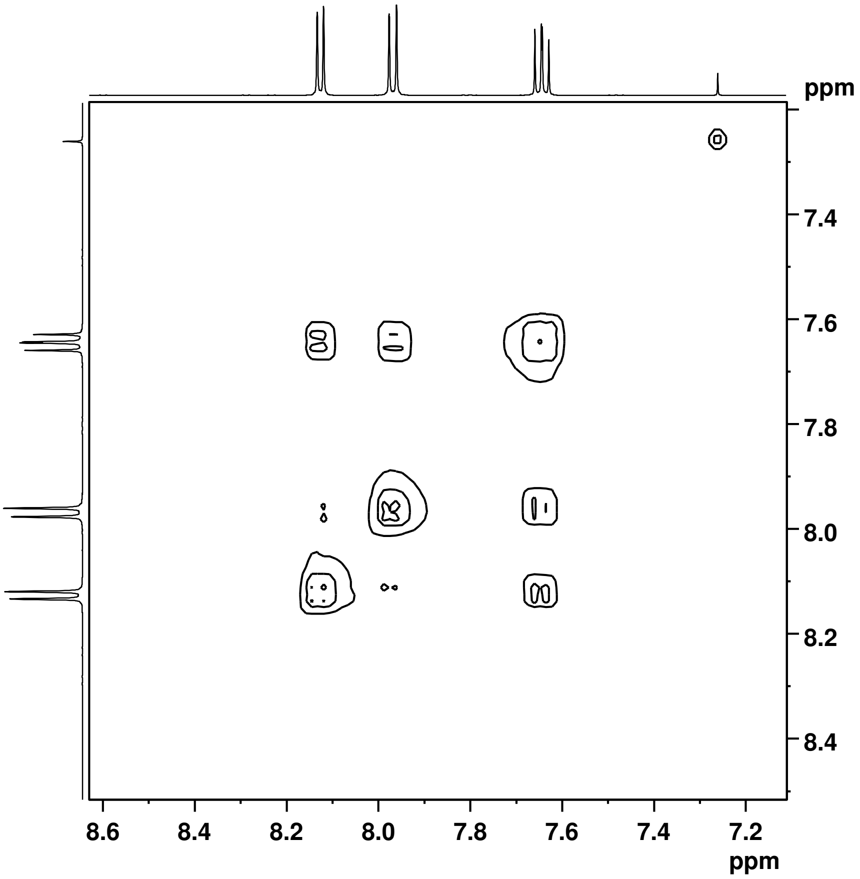

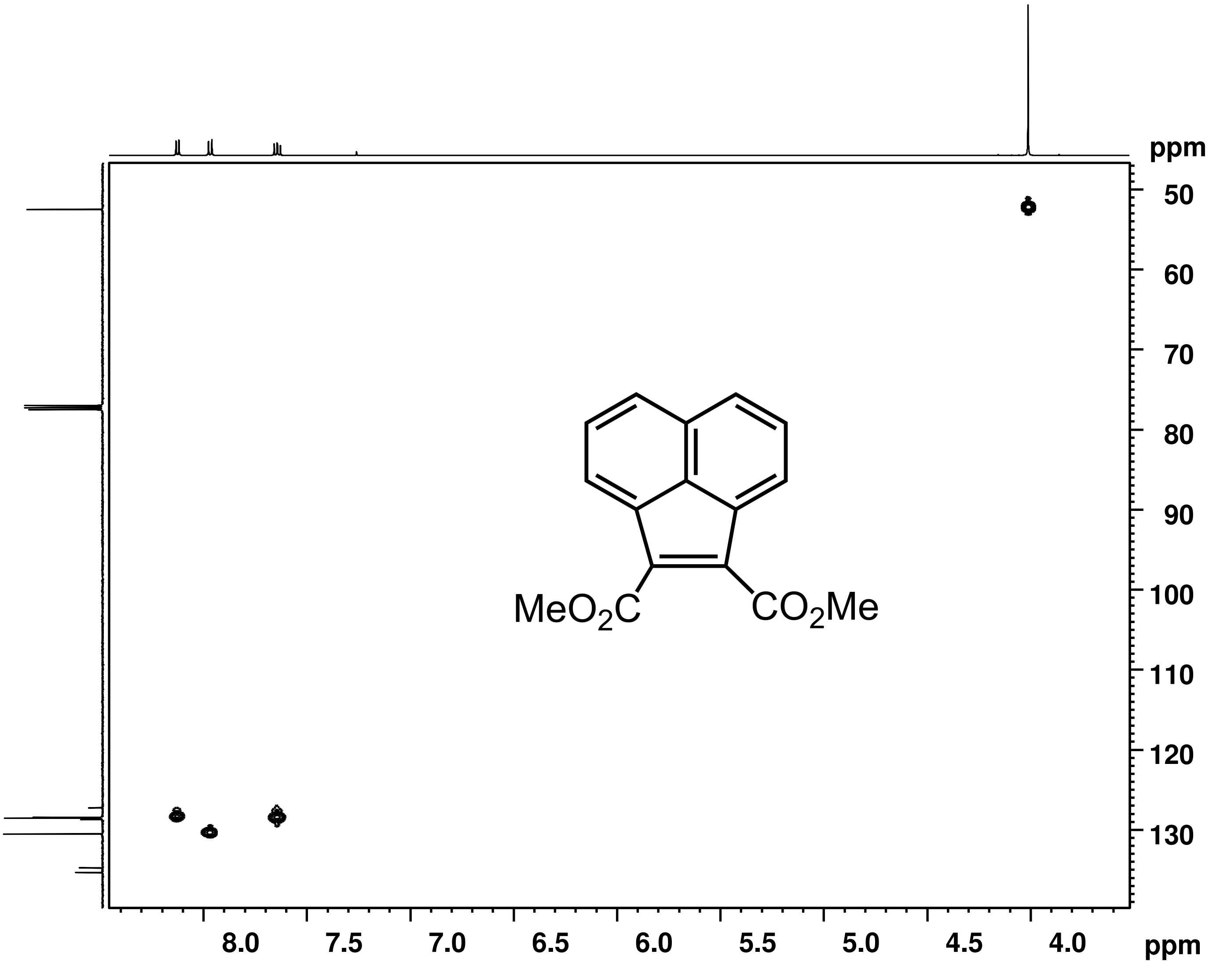

Figure S23. ^{1}H-^{1}H COSY (top) and HSQC (bottom) NMR spectra of dimethyl ester **25** in $CDCl_3$.

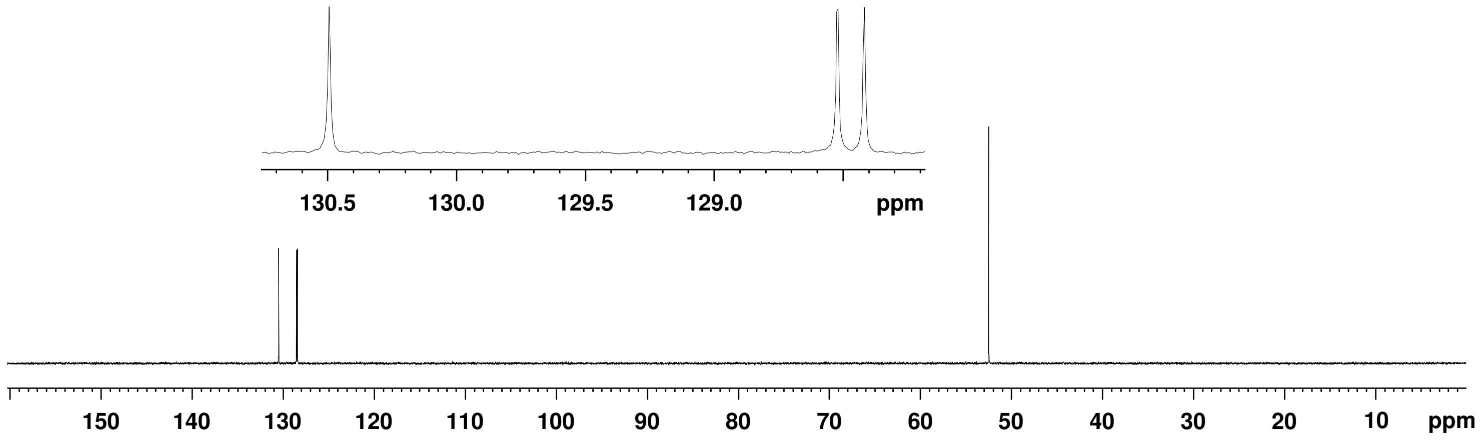

Figure S24. DEPT-135 NMR spectrum of dimethyl ester **25** in $CDCl_3$.

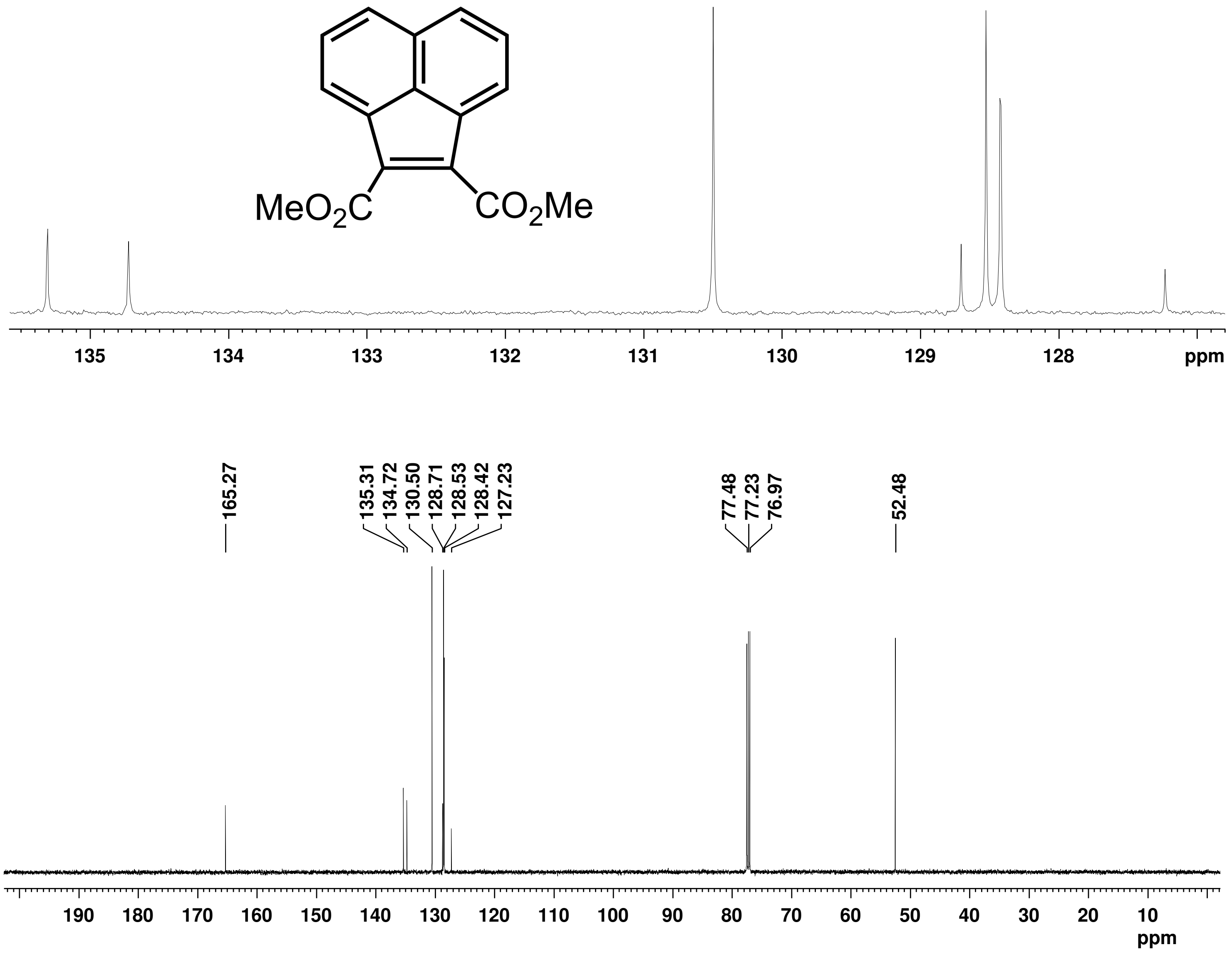

Figure S25. 125 MHz $^{13}C\{^{1}H\}$ NMR spectrum of dimethyl ester **25** in $CDCl_3$.

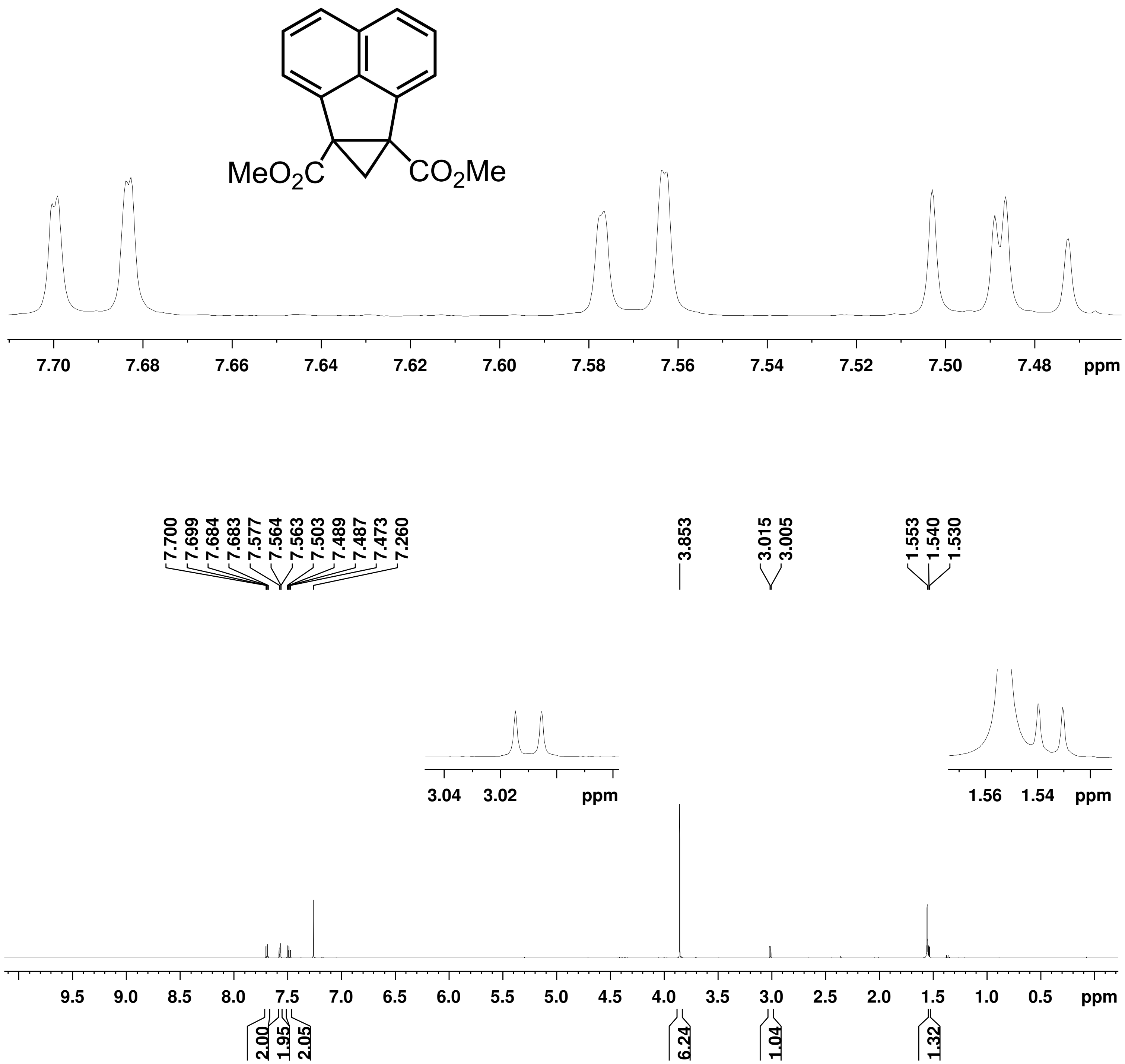

Figure S26. 500 MHz proton NMR spectrum of methanoacenaphthene dimethyl ester **26** in $CDCl_3$.

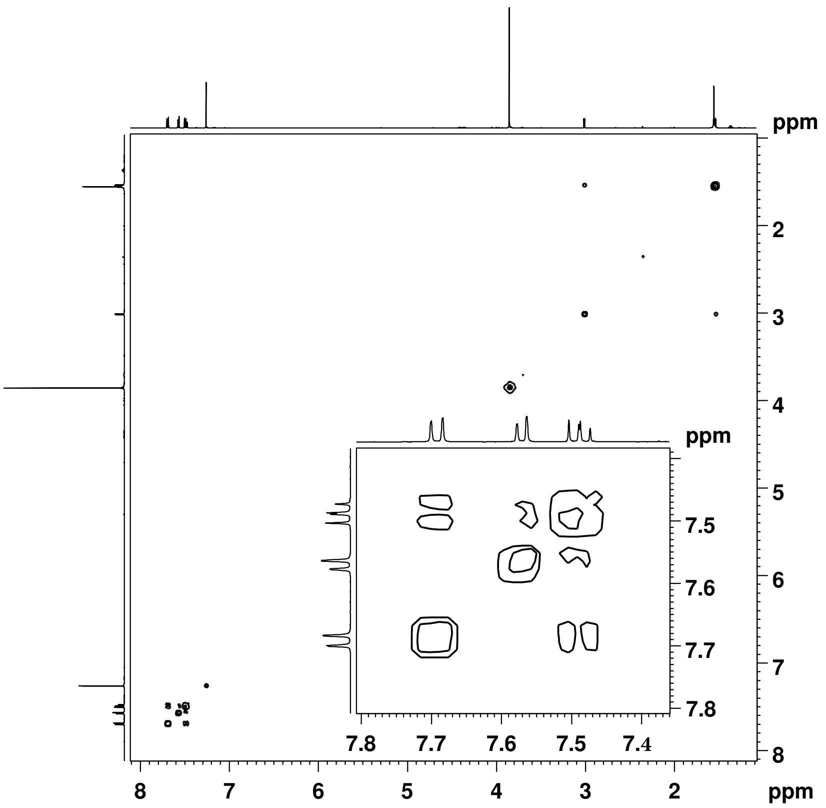

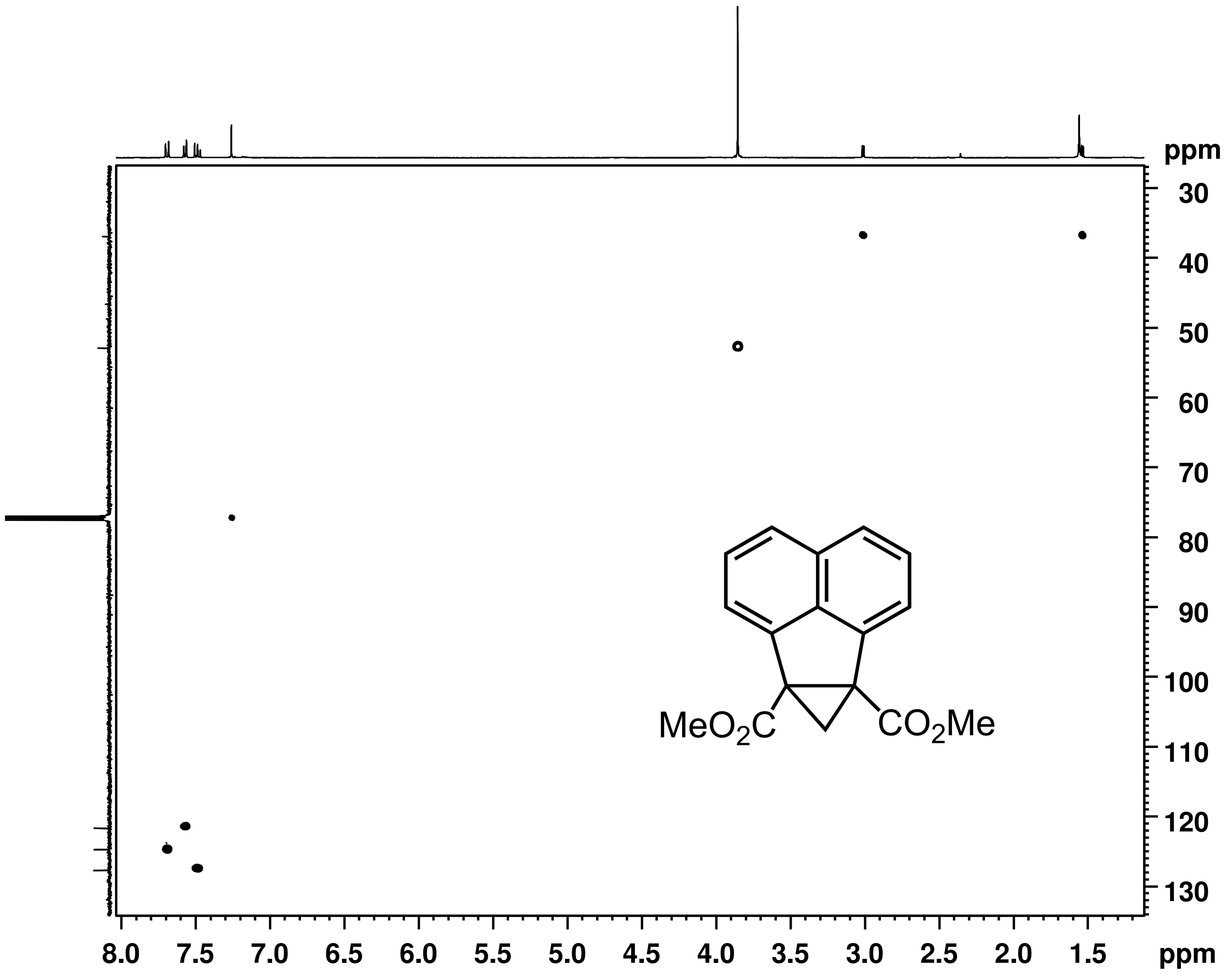

Figure S27. ^{1}H-^{1}H COSY (top) and HSQC (bottom) NMR spectra of **26** in $CDCl_3$.

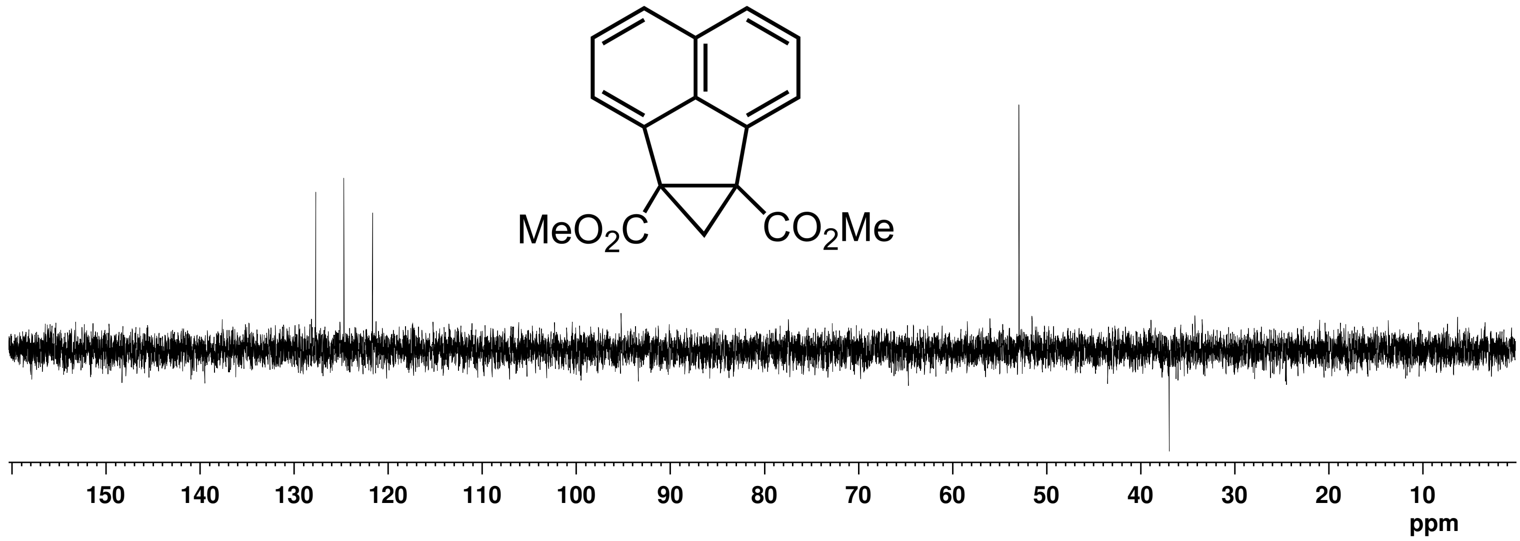

Figure S28. DEPT-135 NMR spectrum of methanoacenaphthene dimethyl ester **26** in $CDCl_3$.

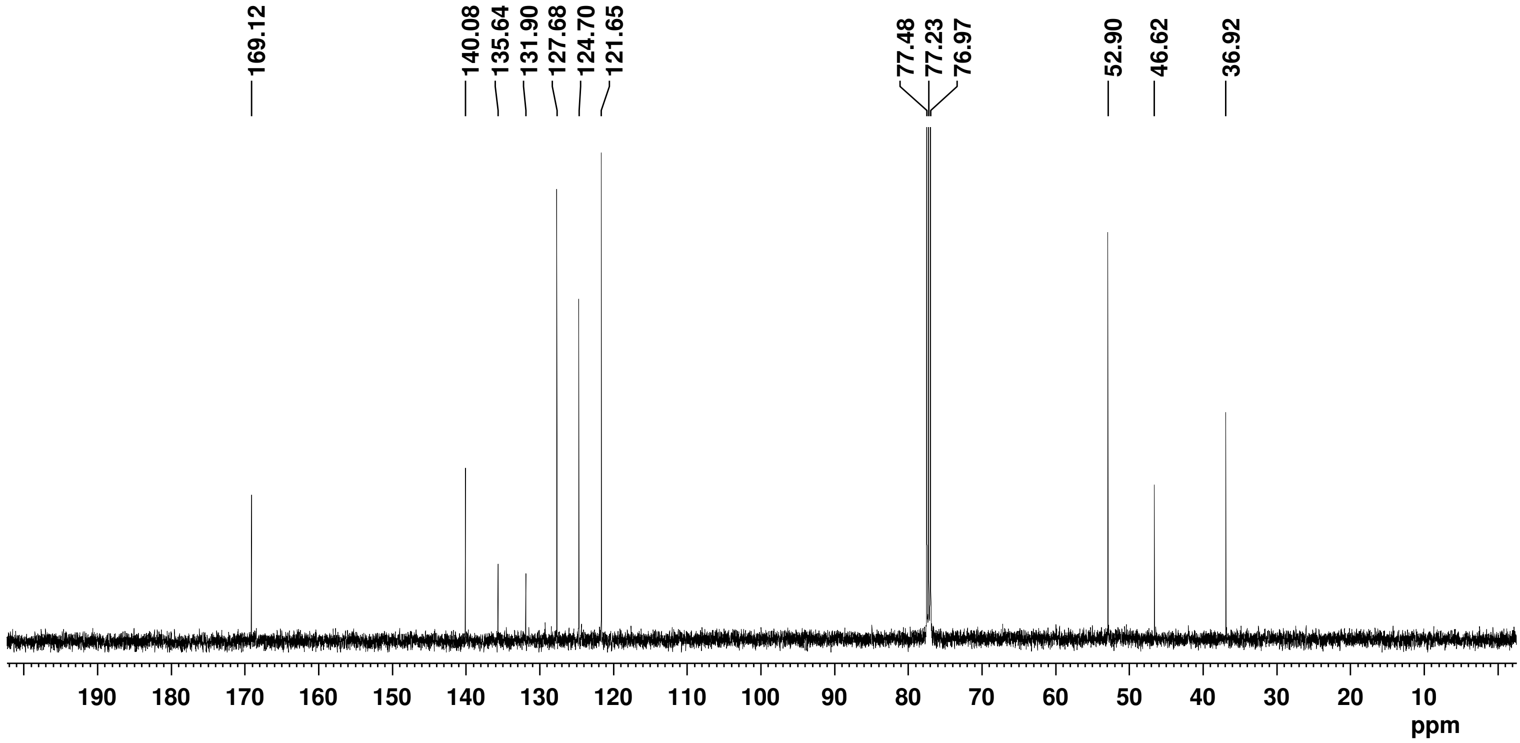

Figure S29. 125 MHz $^{13}C\{^{1}H\}$ NMR spectrum of methanoacenaphthene dimethyl ester **26** in $CDCl_3$.

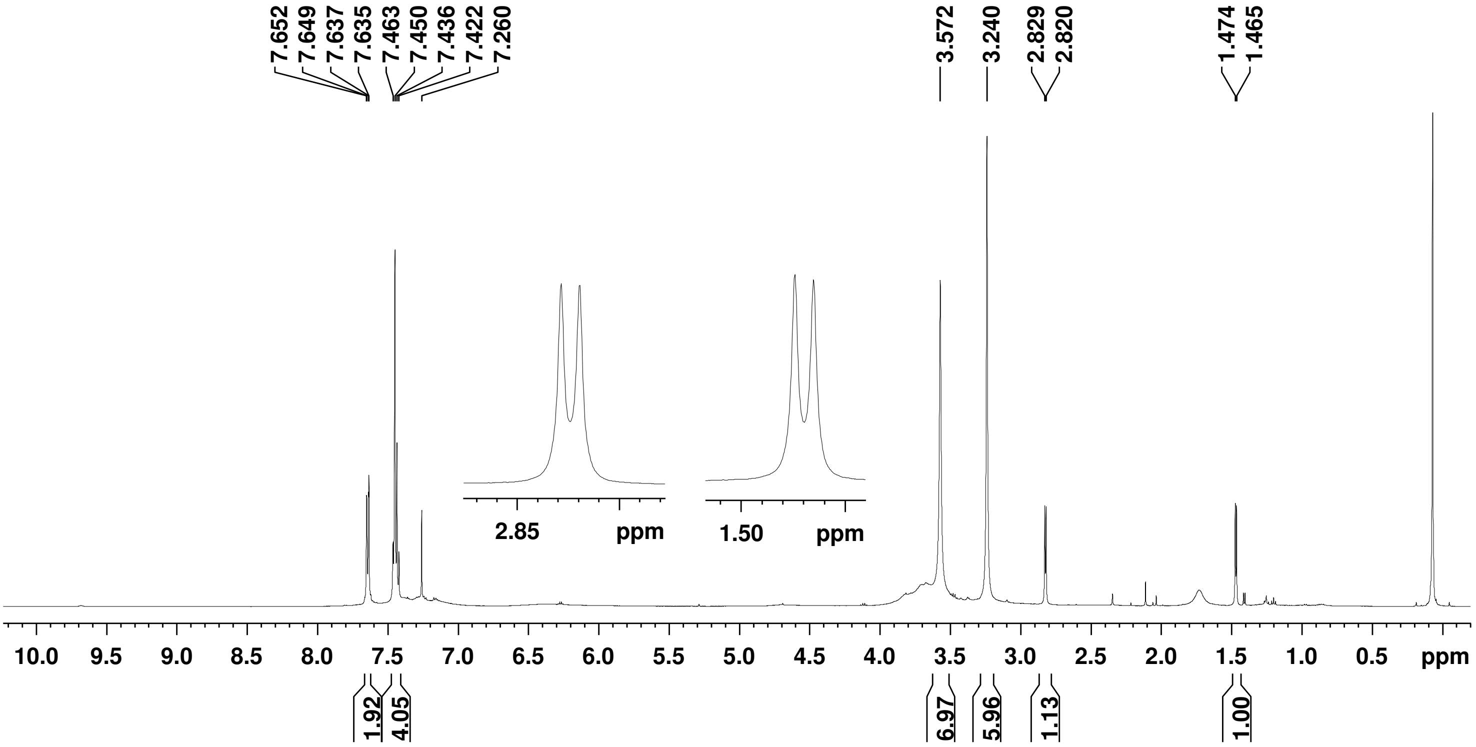

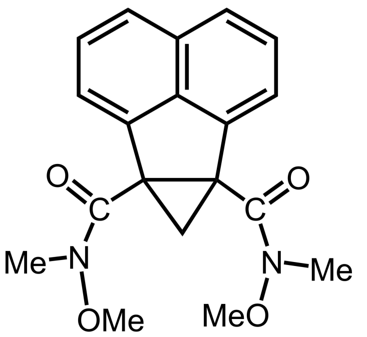

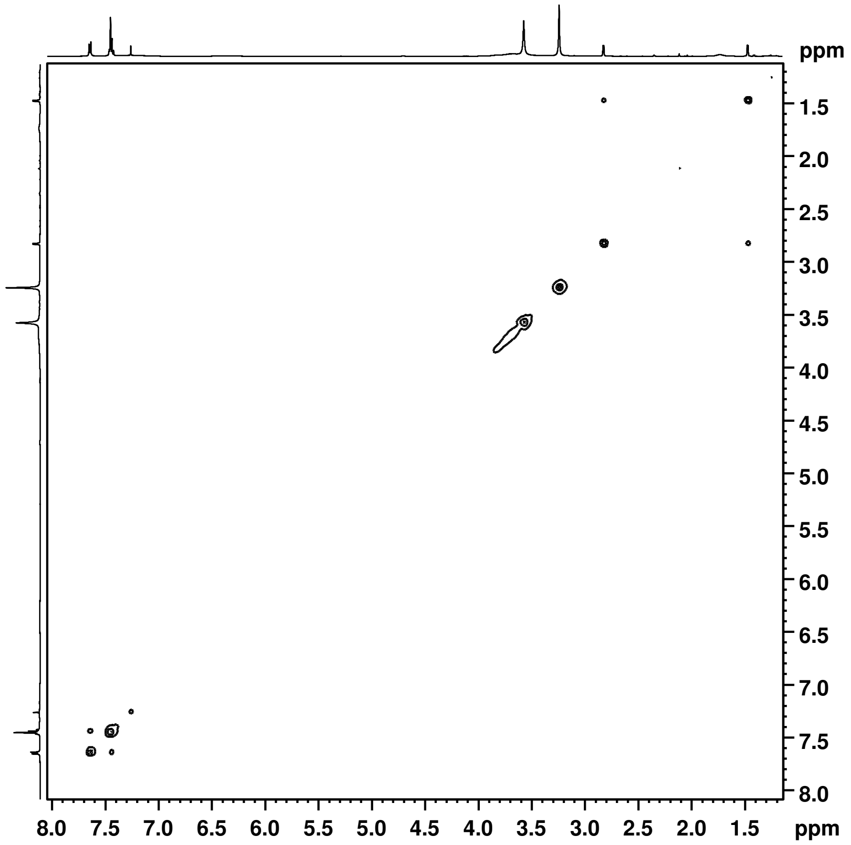

Figure S30. 500 MHz proton (top) and ^{1}H-^{1}H COSY (right) NMR spectra of diamide **29** in $CDCl_3$.

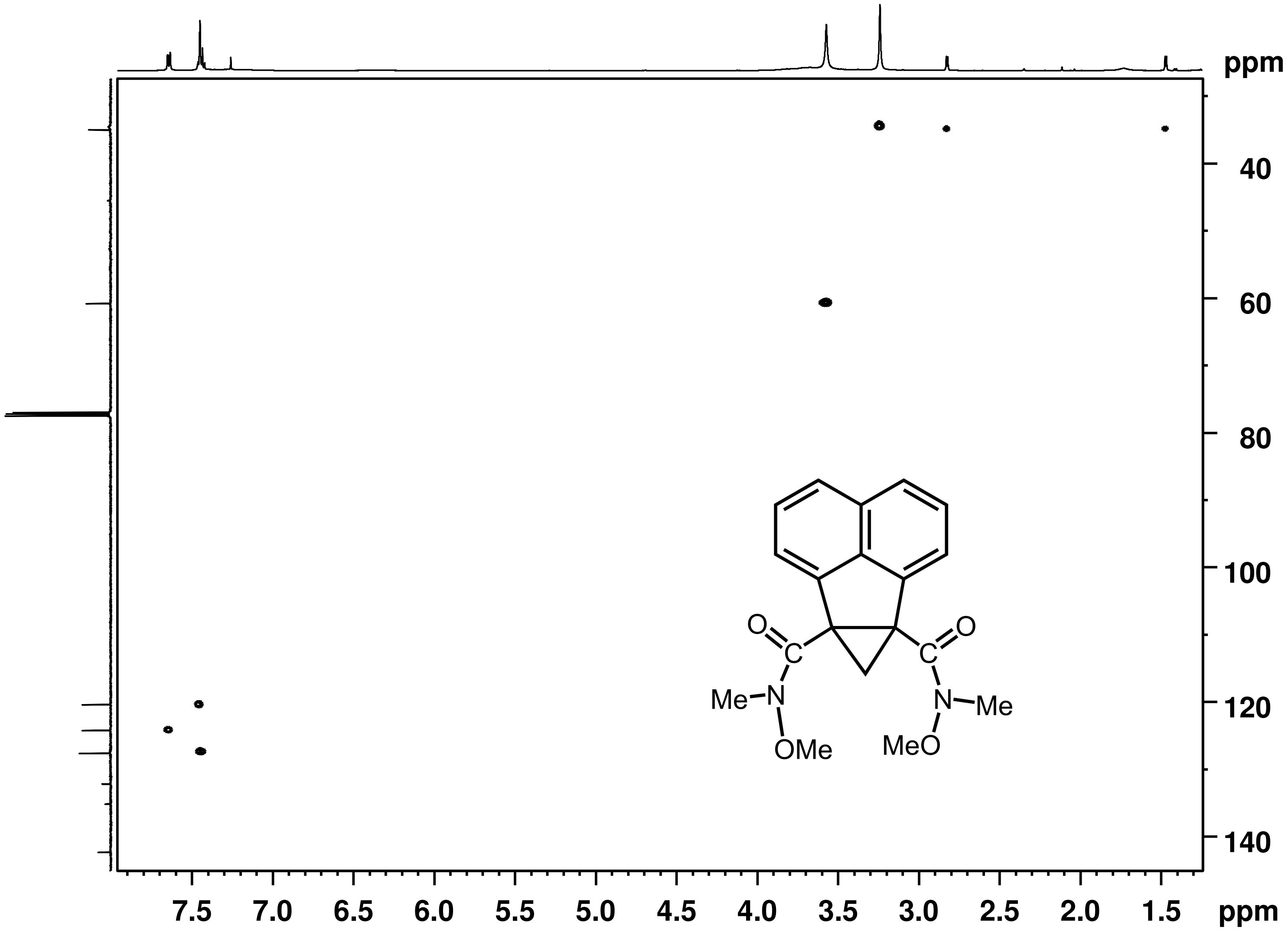

Figure S31. HSQC NMR spectrum of diamide **29** in $CDCl_3$.

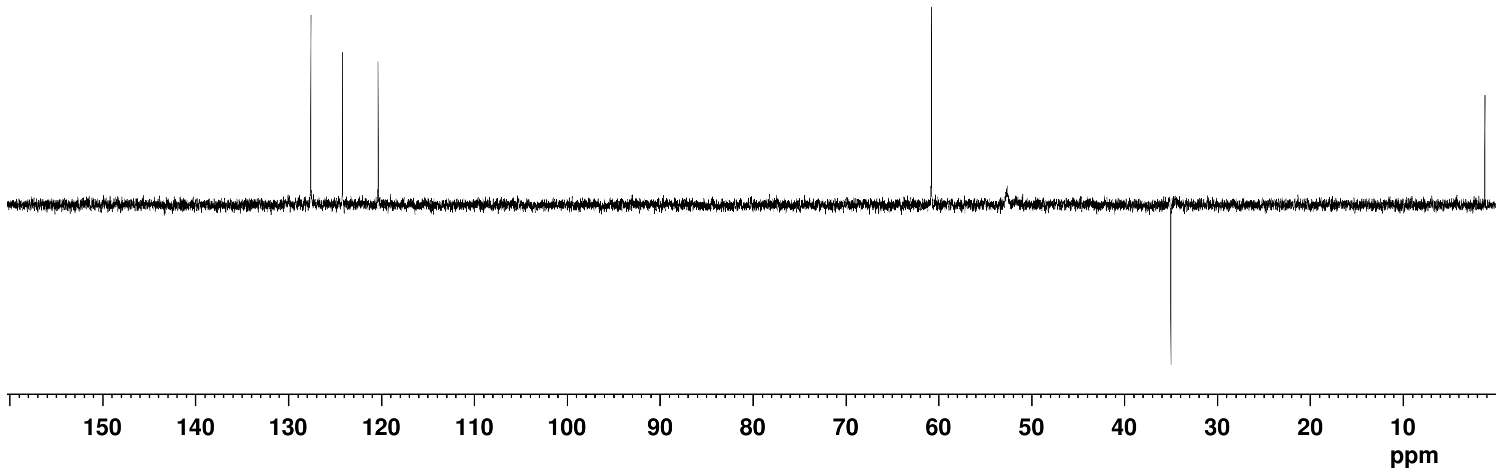

Figure S32. DEPT-135 NMR spectrum of diamide **29** in $CDCl_3$.

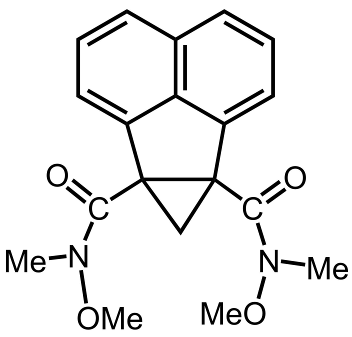

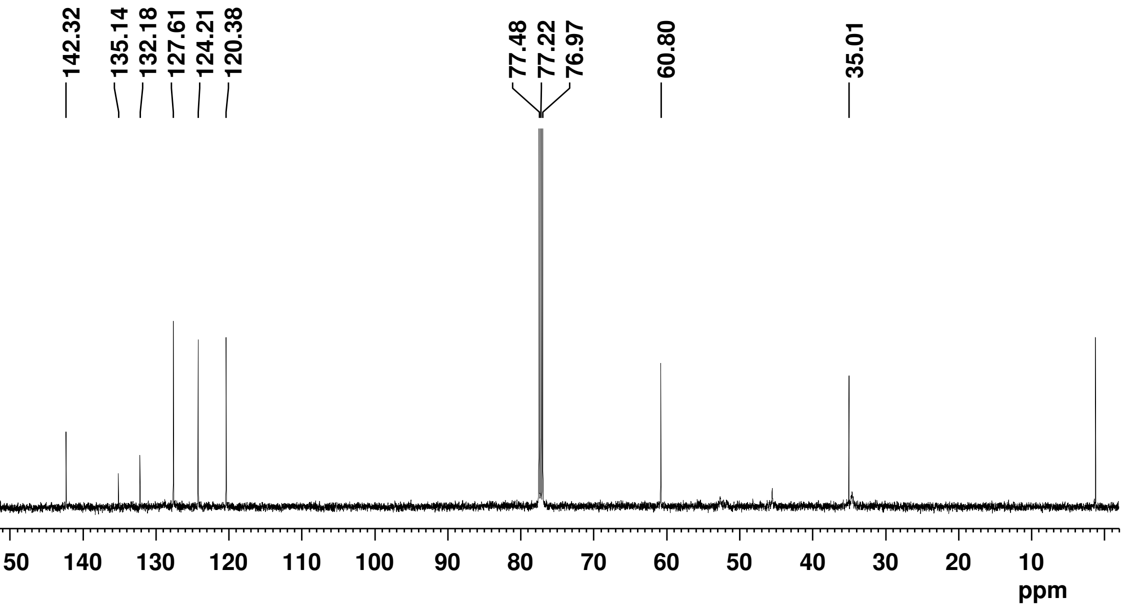

Figure S33. 125 MHz $^{13}C\{^{1}H\}$ NMR spectrum of diamide **29** in $CDCl_3$.

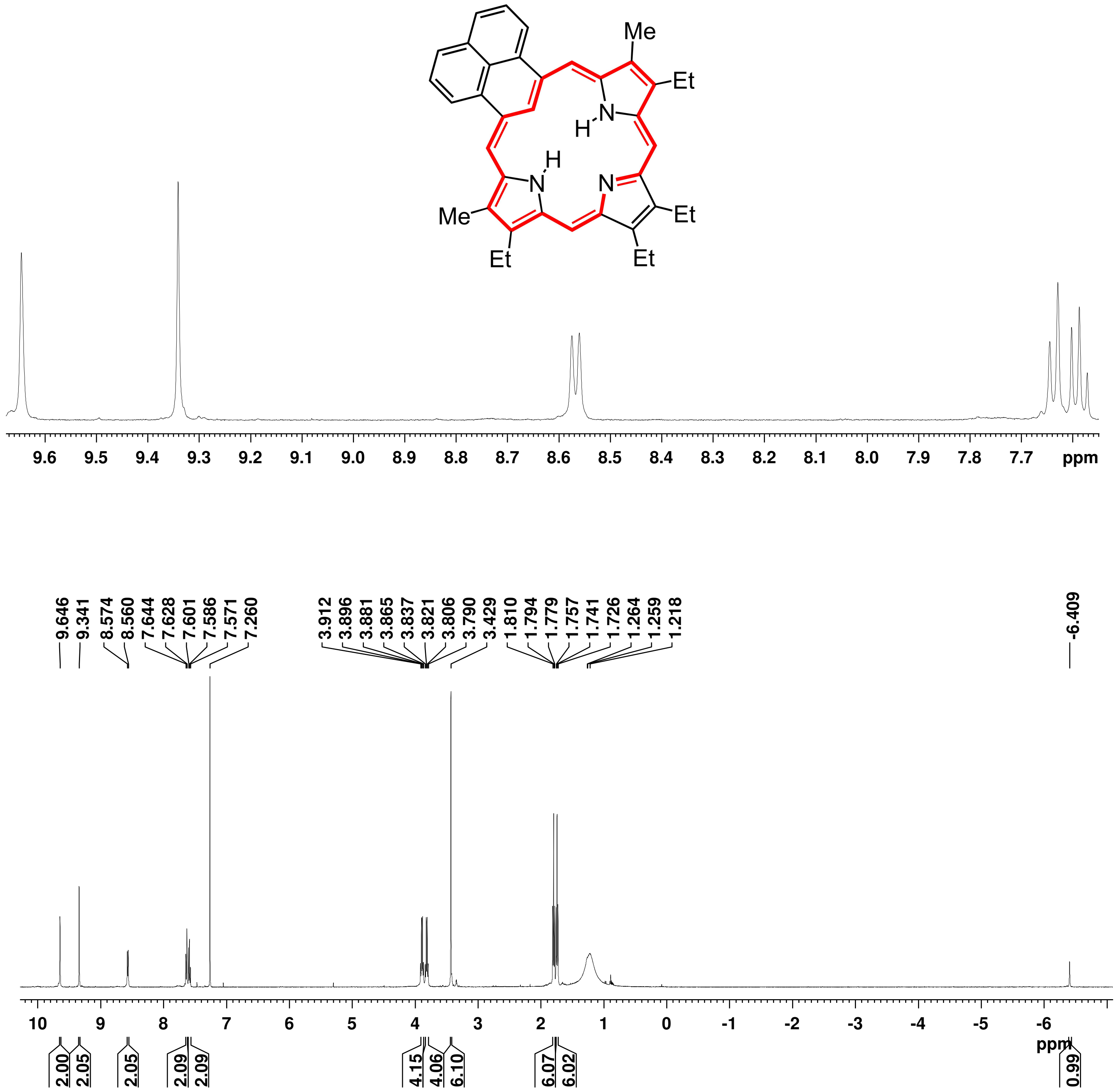

Figure S34. 500 MHz proton NMR spectrum of phenaliporphyrin **9** in $CDCl_3$.

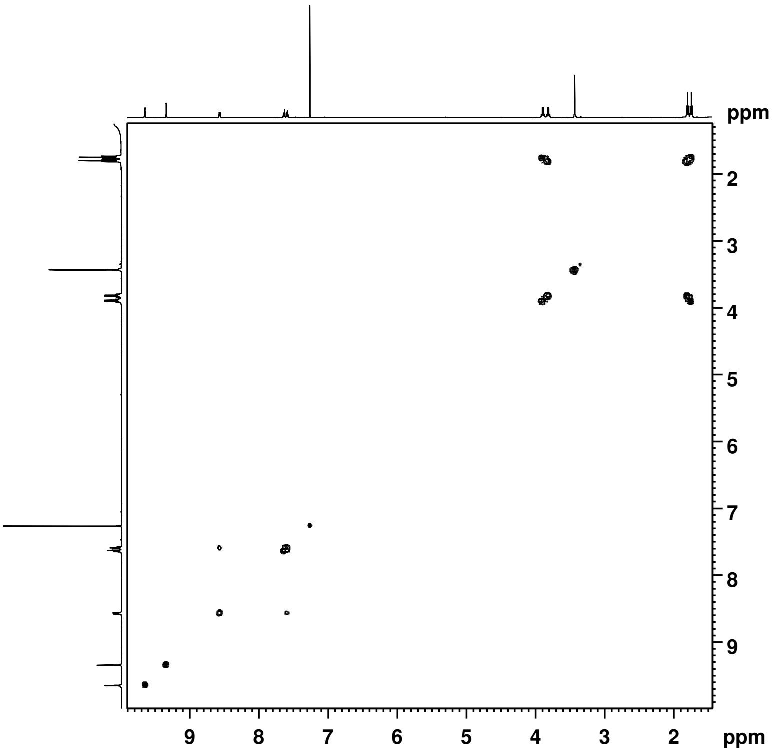

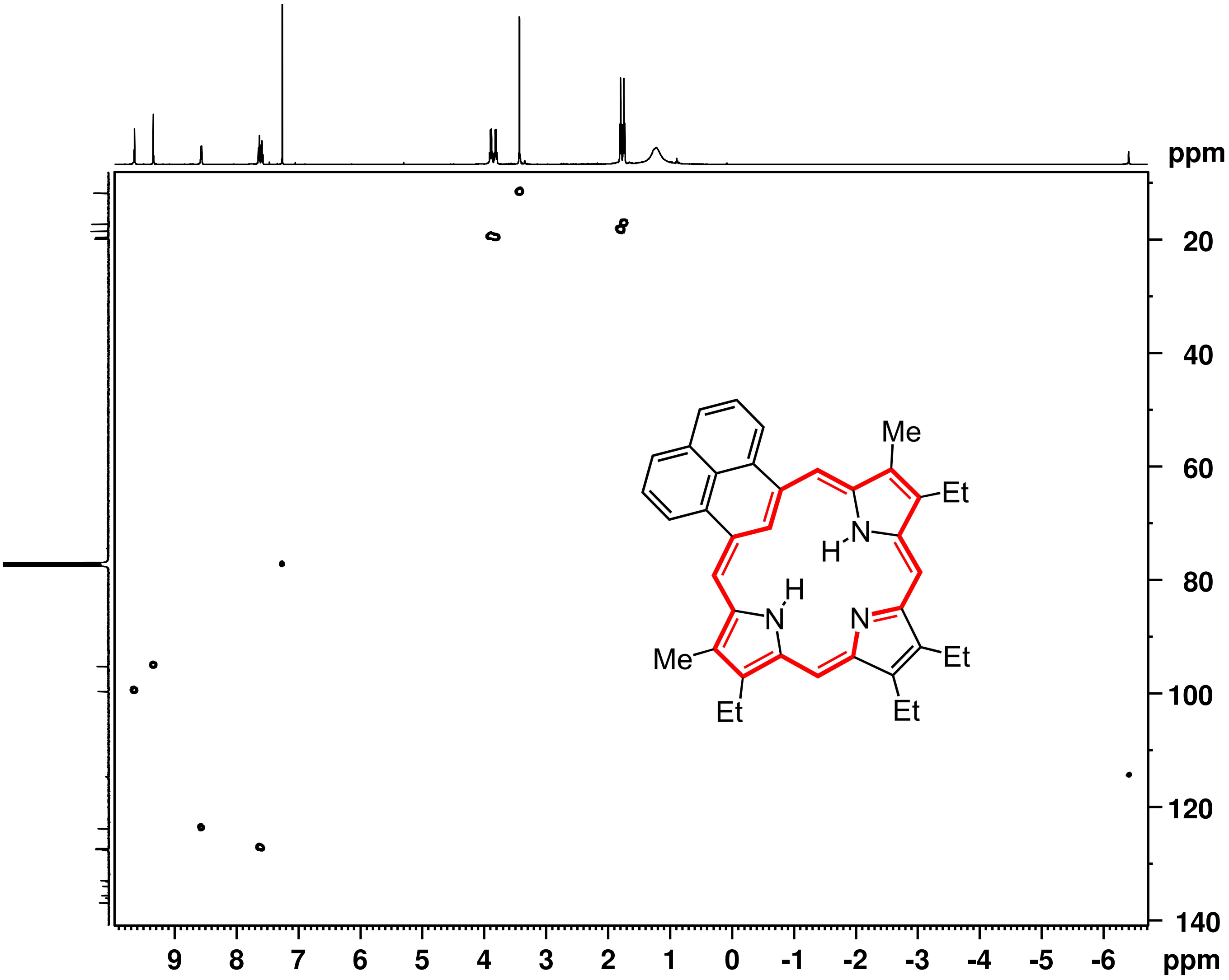

Figure S35. ^{1}H-^{1}H COSY (top) and HSQC (bottom) NMR spectra of phenaliporphyrin **9** in $CDCl_3$.

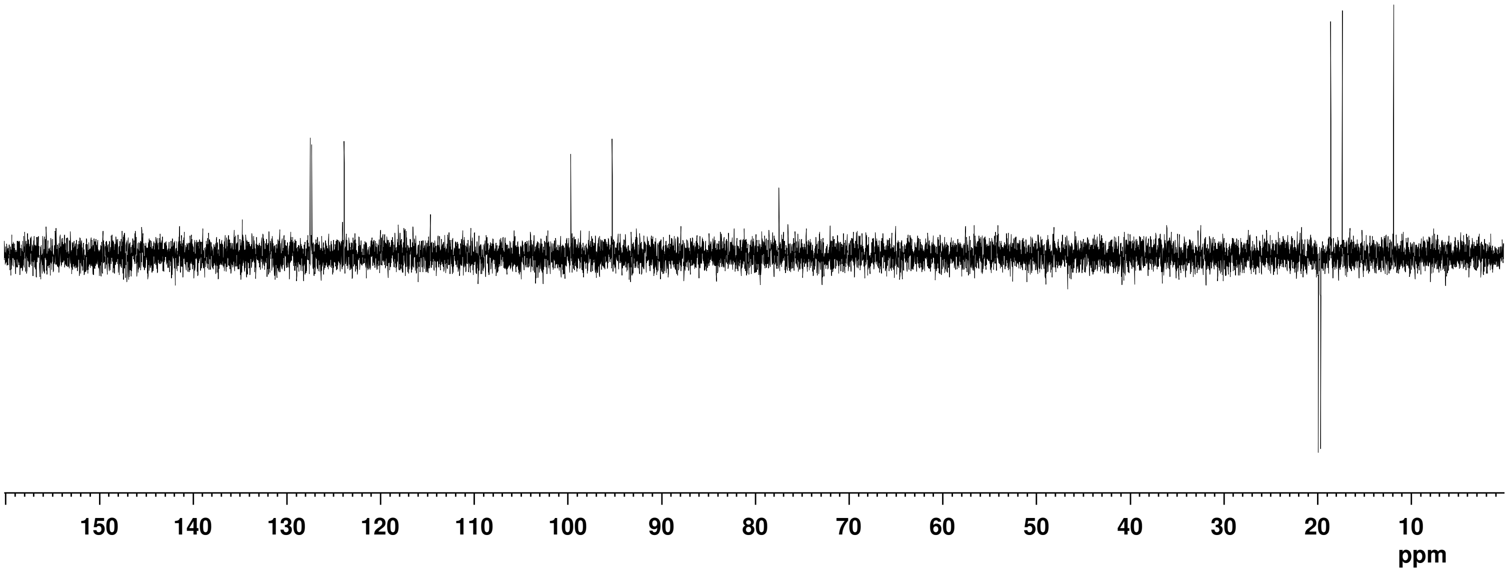

Figure S36. DEPT-135 NMR spectrum of phenaliporphyrin **9** in $CDCl_3$.

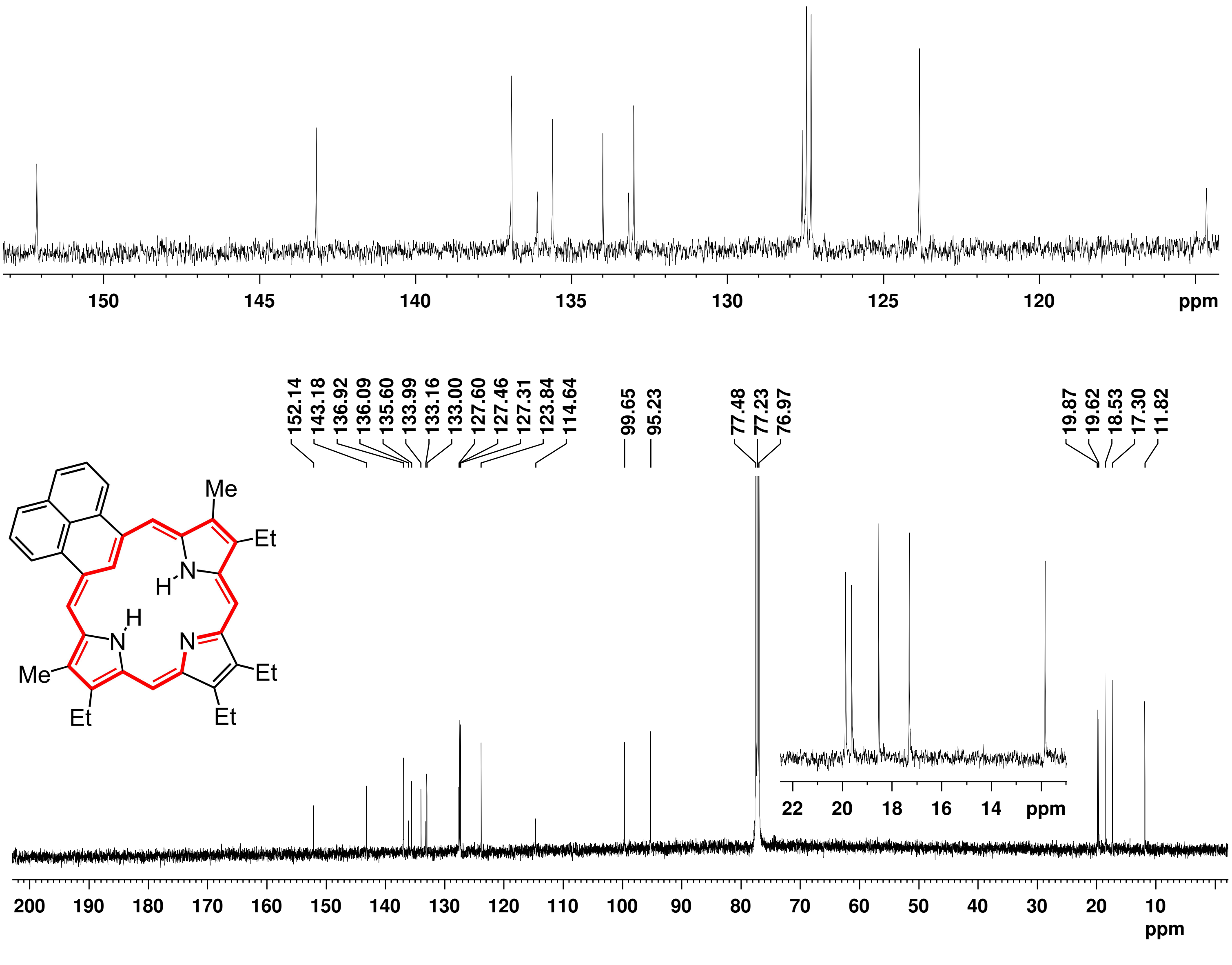

Figure S37. 125 MHz $^{13}C\{^{1}H\}$ NMR spectrum of phenaliporphyrin **9** in $CDCl_3$.

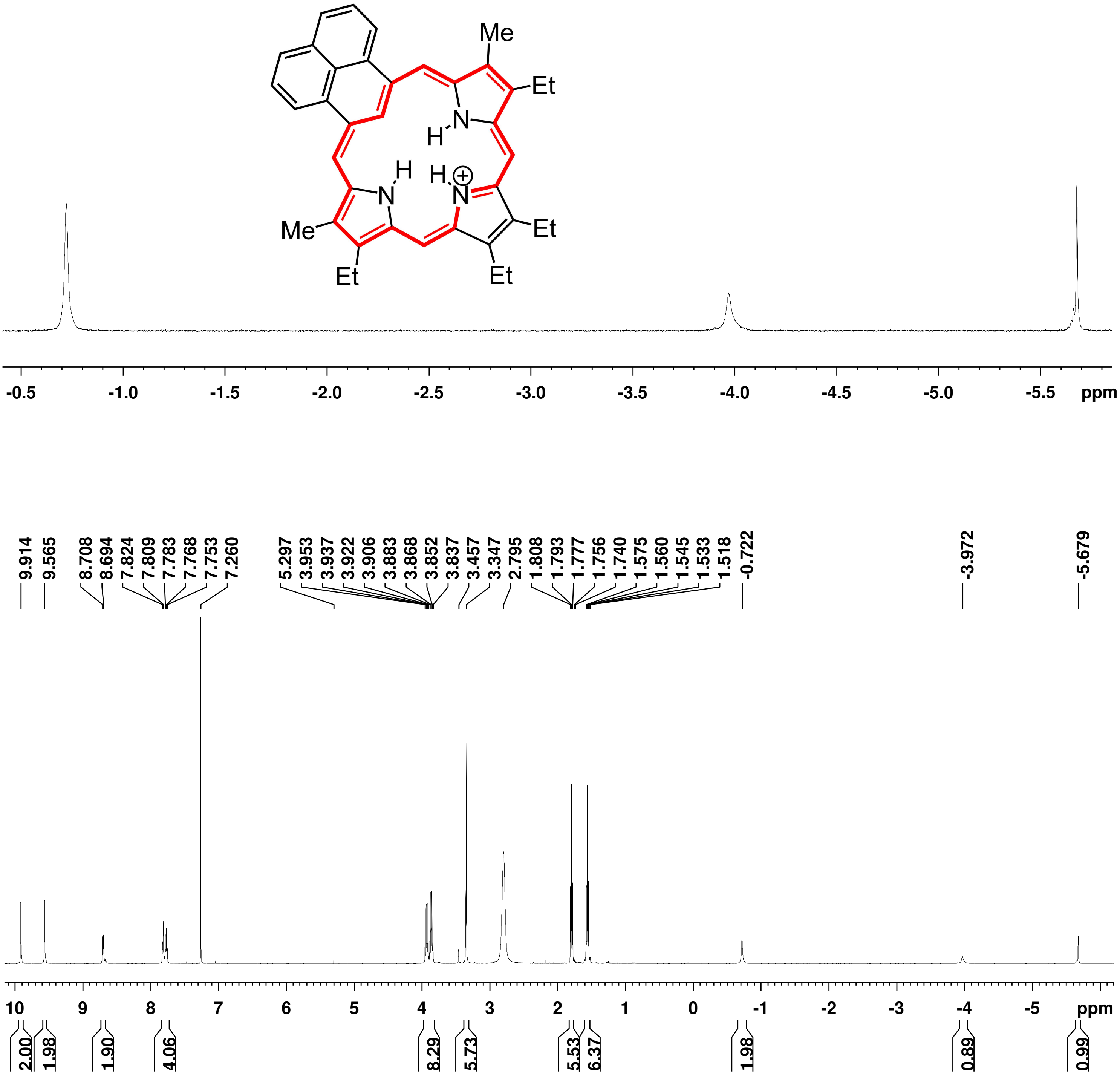

Figure S38. 500 MHz proton NMR spectrum of monoprotonated phenaliporphyrin **9**H$^+$ in $CDCl_3$.

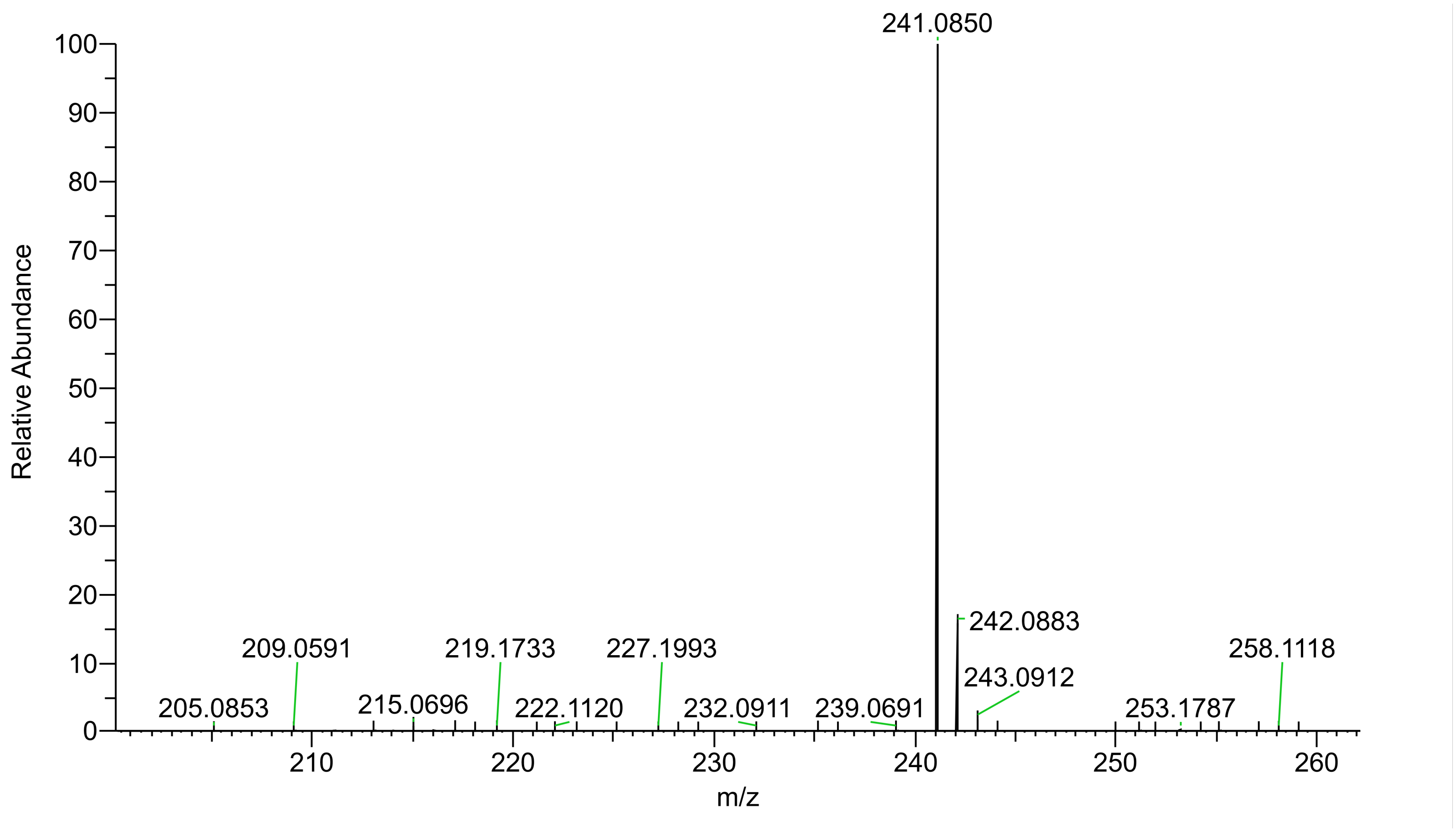

Figure S39. High resolution TOF-ESI MS of keto ester **16**.

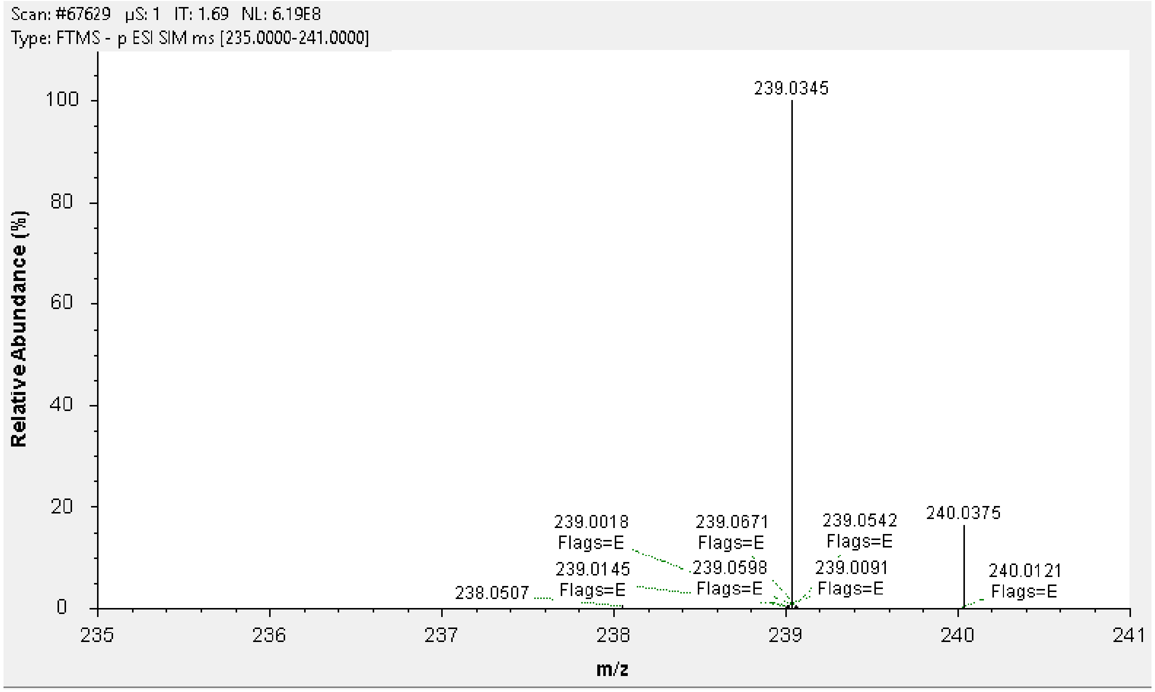

Figure S40. High resolution TOF-ESI mass spectrum of 1,2-acenaphthylendicarboxylic acid (**24**).

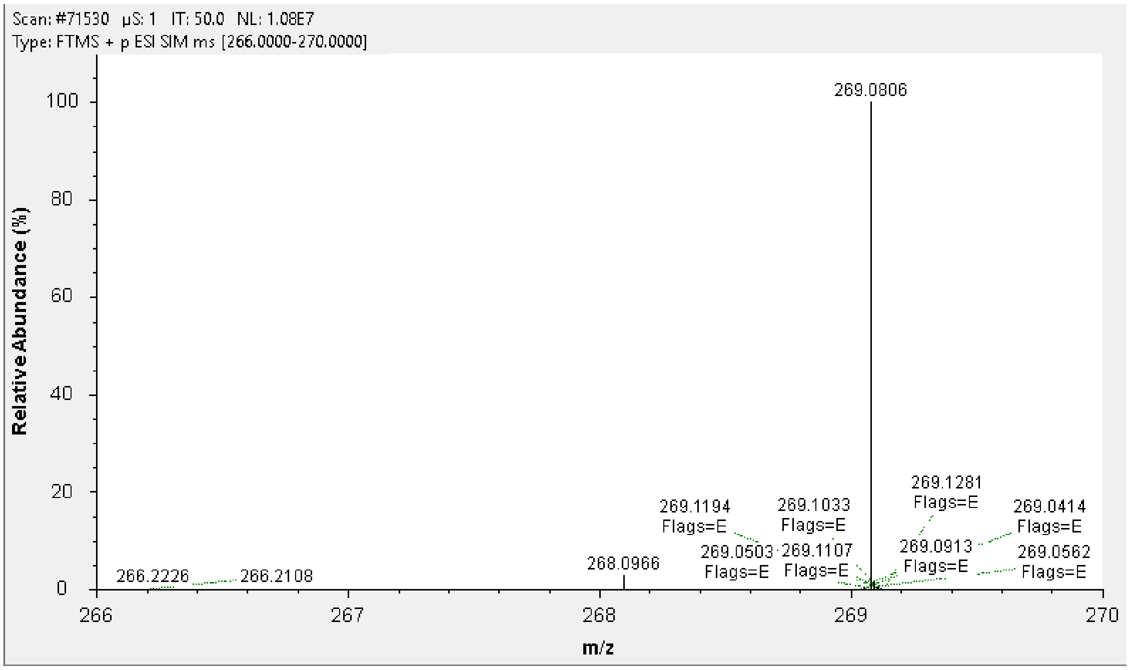

Figure S41. High resolution TOF-ESI MS of dimethyl 1,2-acenaphthylendicarboxylate (**25**).

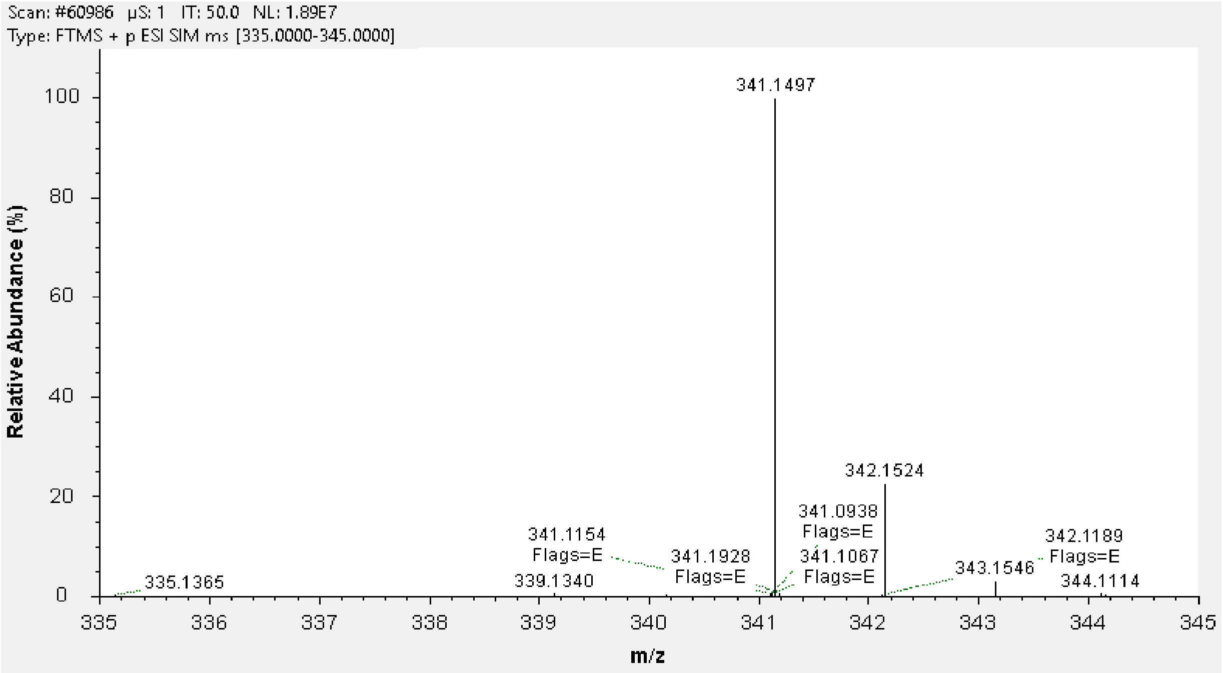

Figure S42. High resolution TOF-ESI MS of dimethyl 1,2-methanoacenaphthenedicarboxylate (**26**).

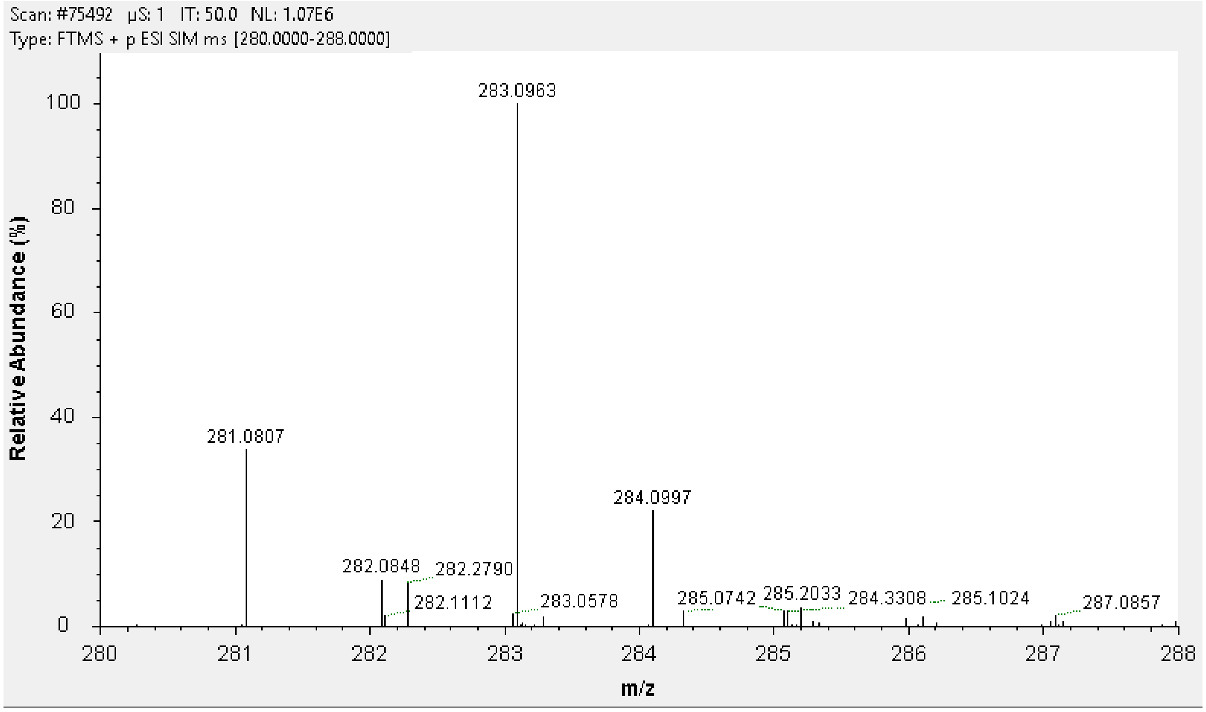

Figure S43. High resolution TOF-ESI MS of bis(*N*-methoxy-*N*-methylamide **29**.

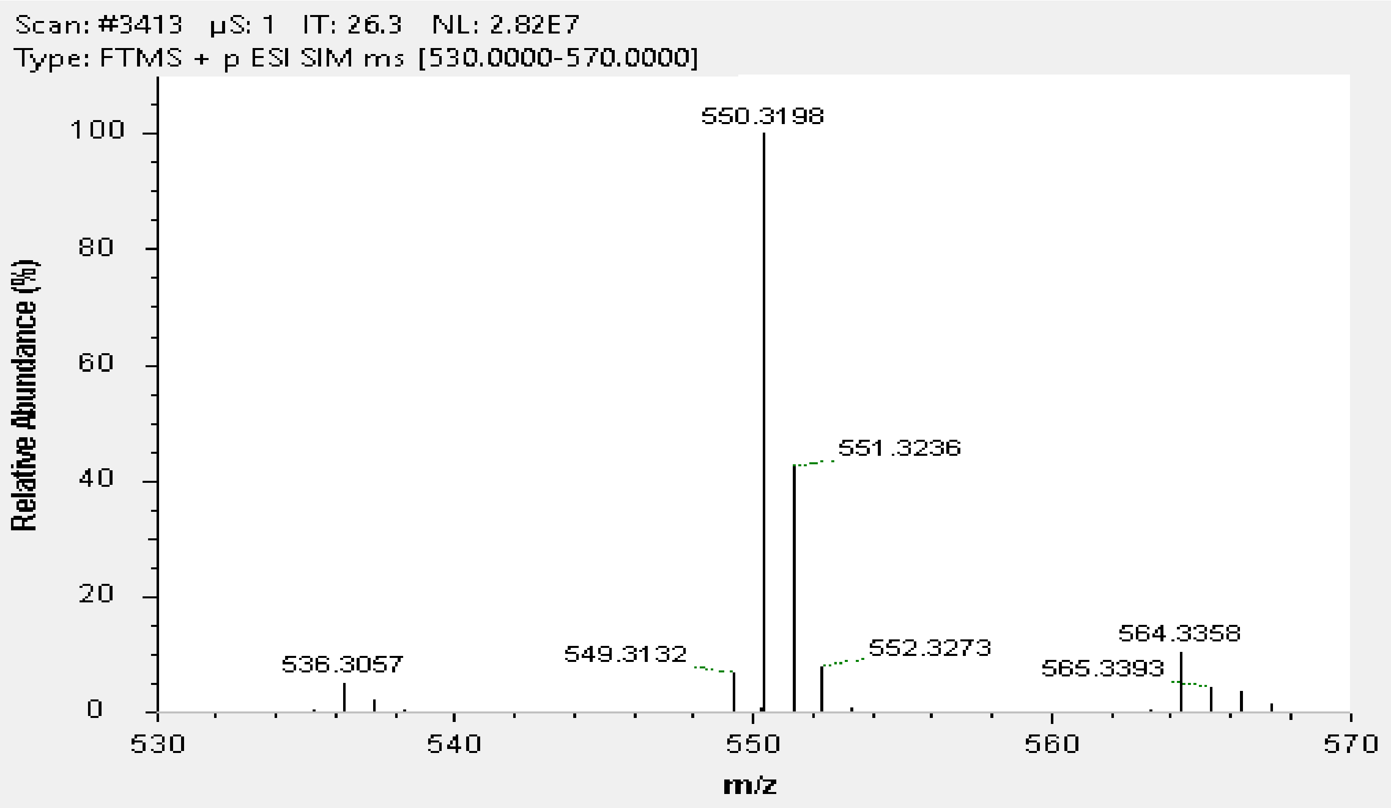

Figure S44. High resolution TOF-ESI mass spectrum of phenaliporphyrin **9**.

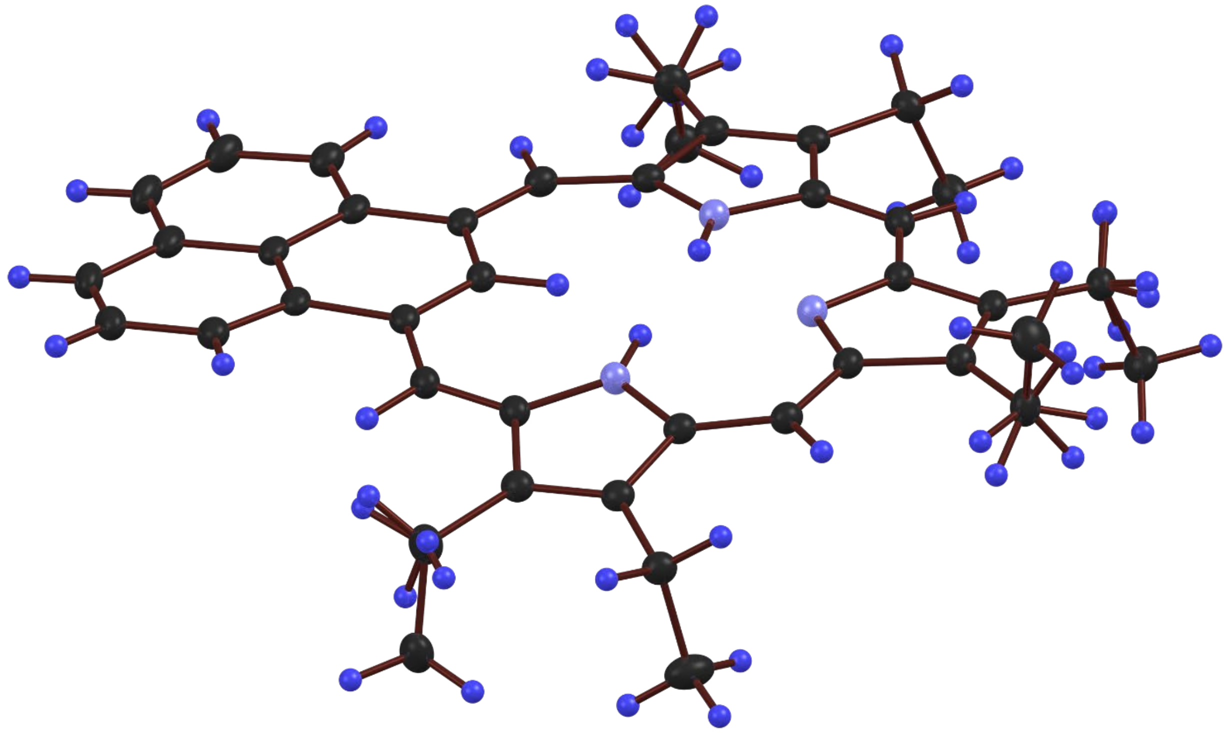

Figure S45. Color POV-Ray rendered ORTEP III drawing (50% probability level, hydrogen atoms rendered arbitrarily small for clarity) including "disorder" of major and minor component of phenaliporphyrin **9**.

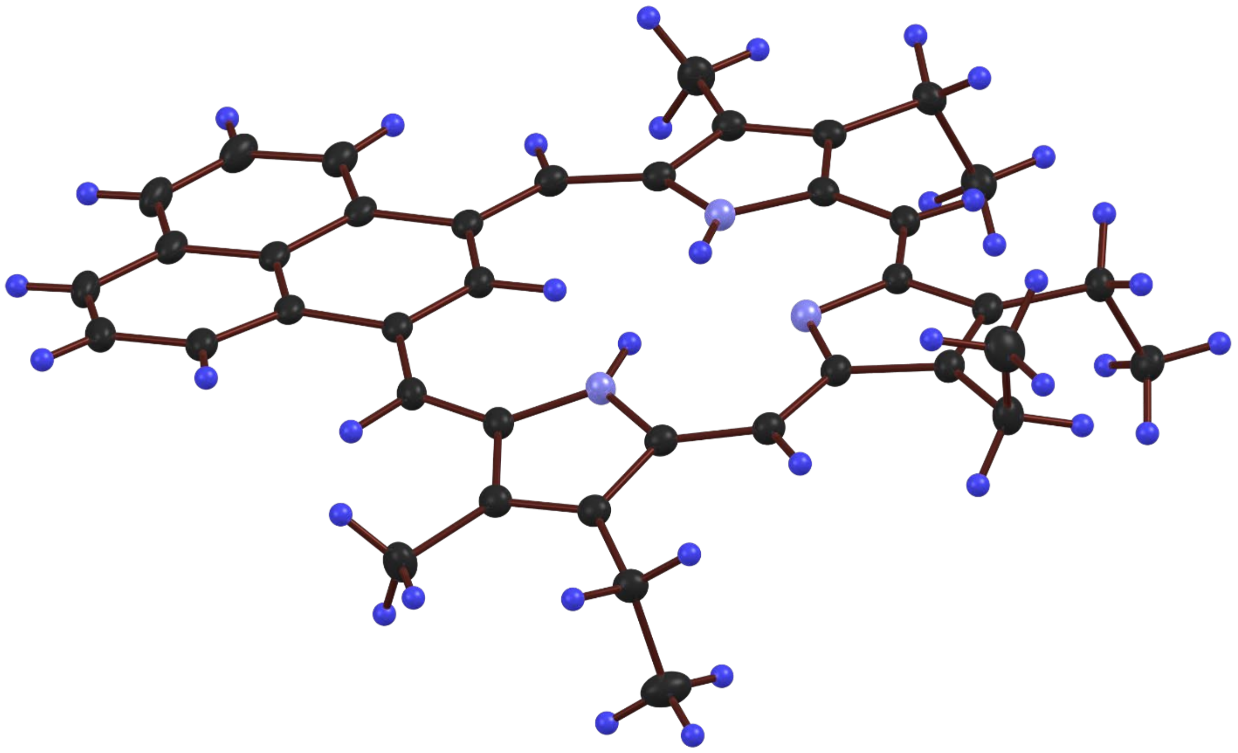

Figure S46 Color POV-Ray rendered ORTEP III drawing (50% probability level, hydrogen atoms rendered arbitrarily small for clarity) of major component of **9**.

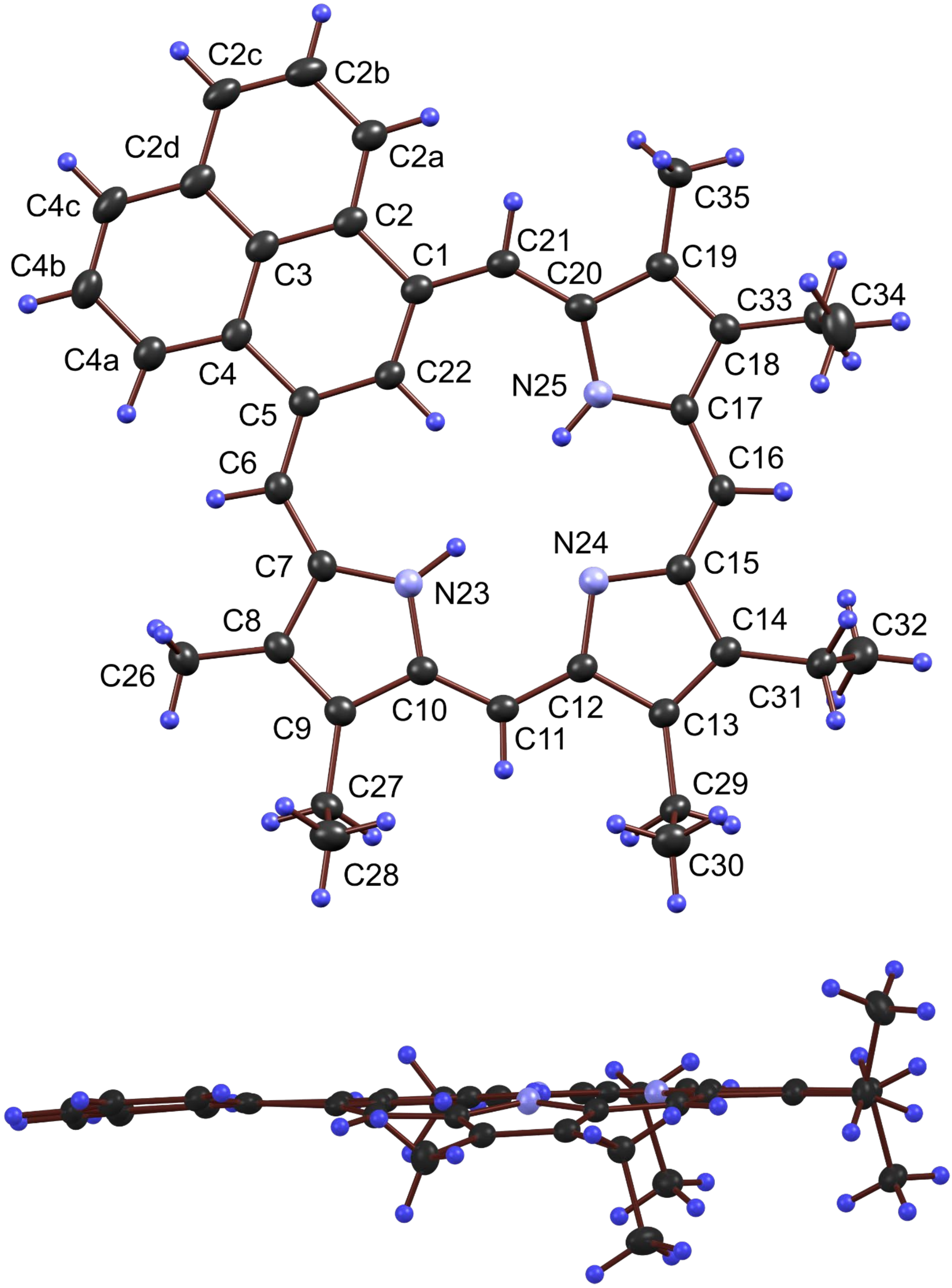

Figure S47. Color POV-Ray rendered ORTEP III drawing (50% probability level, hydrogen atoms rendered arbitrarily small for clarity) of major component of **9**.

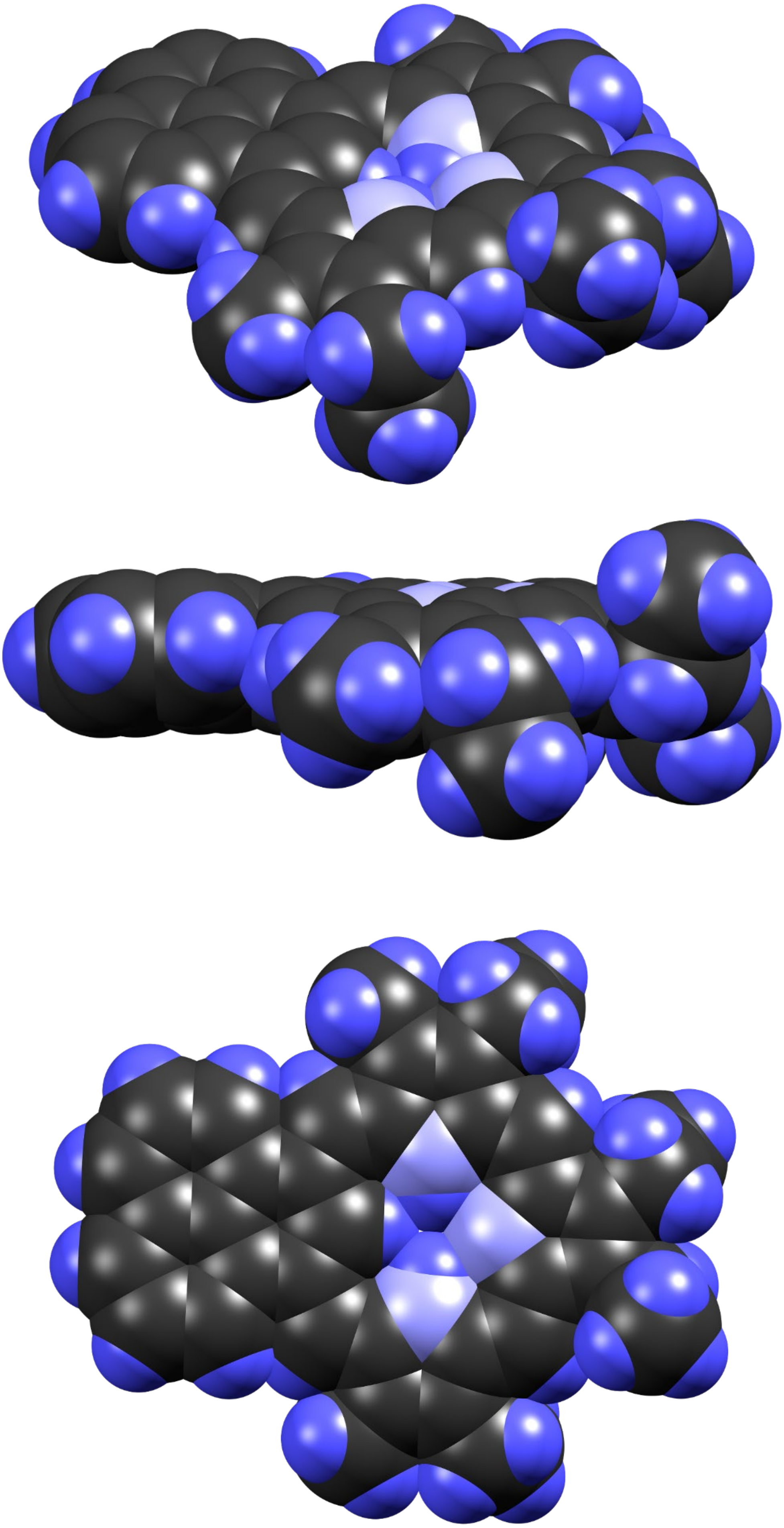

Figure S48. Color POV-Ray rendered space-filling drawing of phenaliporphyrin **9**.

Visual reference of bond lengths:

Table S1: Summary of framework bond distances (Å) and angles (°) for **9**.

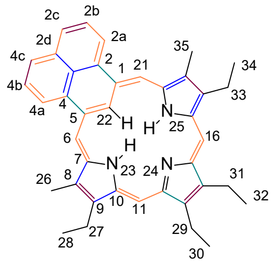

Phenaliporphyrin **9**

C1-C22	1.401(1)
C1-C2	1.480(1)
C2-C2a	1.390 (2)
C2a-C2b	1.405(2)
C2b-C2c	1.366(2)
C2c-C2d	1.415(2)
C2-C3	1.430(2)
C2d-C3	1.430(2)
C3-C4	1.432(2)
C4-C4a	1.388 (2)
C4a-C4b	1.406(2)
C4b-C4c	1.364(2)
C4c-C2d	1.416(2)
C4-C5	1.481(1)
C5-C22	1.401(2)
C5-C6	1.406(2)
C6-C7	1.391(2)
C7-N23	1.376(1)
C7-C8	1.444(1)
C8-C9	1.375(2)

C9-C10	1.431(1)
C10-N23	1.373(1)
C10-C11	1.388(2)
C12-N24	1.371(1)
C11-C12	1.397(1)
C12-C13	1.461(1)
C13-C14	1.360(2)
C14-C15	1.462(1)
C15-N24	1.371(1)
C15-C16	1.399(1)
C16-C17	1.391(2)
C17-N25	1.375(1)
C17-C18	1.430(1)
C18-C19	1.376(2)
C19-C20	1.439(1)
C20-N25	1.382(1)
C20-C21	1.393(2)
C1-C21	1.408(2)
C1-C22-C5	124.6(1)

Orange – more aromatic (1.38-1.41), green – single bond limit (≥1.46), blue – more single bond like (1.42-1.45), purple more double bond like (≤1.37).

Table S2: Fractional Atomic Coordinates (×10[4]) and Equivalent Isotropic Displacement Parameters (Å[2]×10[3]) for **9**. U_{eq} is defined as 1/3 of the trace of the orthogonalised U_{ij}.

Atom	x	y	z	U_{eq}
C26	2725.6(13)	4590.3(6)	2261.8(8)	24.7(2)
C29	8187.7(11)	2652.2(5)	6584.6(8)	19.8(2)
C30	7382.4(13)	2042.4(5)	6230.8(9)	23.9(3)
C31	8088.6(12)	2886.4(5)	8735.9(8)	22.4(2)
C32	9361.3(13)	3286.4(6)	9325.5(9)	30.0(3)
C35	1873.9(13)	5104.8(6)	9374.3(8)	25.8(2)
C26b	2725.6(13)	4590.3(6)	2261.8(8)	24.7(2)
C41b	1660(30)	4145(13)	1640(20)	24.9(7)
C29b	8187.7(11)	2652.2(5)	6584.6(8)	19.8(2)
C31b	8088.6(12)	2886.4(5)	8735.9(8)	22.4(2)
C35b	1873.9(13)	5104.8(6)	9374.3(8)	25.8(2)
C42b	750(30)	4687(13)	9490(20)	25.9(7)
N23	4018.0(9)	4357.3(4)	4904.8(6)	17.14(18)
N24	5524.2(9)	3875.6(4)	6799.2(6)	17.63(18)
N25	3839.5(10)	4646.4(4)	7691.0(6)	17.85(18)
C22	1948.4(11)	5283.1(5)	5655.7(8)	17.7(2)
C1	1400.1(11)	5577.3(5)	6319.4(8)	17.4(2)
C2	306.1(11)	6072.8(5)	5957.2(8)	18.3(2)
C2a	-232.5(12)	6425.1(5)	6562.8(8)	21.6(2)
C2b	-1356.6(12)	6857.2(5)	6199.5(9)	24.6(2)
C2c	-1946.8(12)	6946.6(5)	5228.3(9)	25.1(2)
C2d	-1389.7(12)	6625.8(5)	4579.6(8)	22.3(2)
C3	-238.4(11)	6188.7(5)	4942.7(8)	18.6(2)
C4	340.0(11)	5876.7(5)	4282.1(8)	18.4(2)
C4a	-226.4(12)	6019.6(5)	3308.6(8)	21.9(2)
C4b	-1382.0(13)	6439.2(5)	2956.3(9)	25.2(2)
C4c	-1963.7(12)	6732.0(5)	3573.4(9)	25.7(2)
C5	1499.7(11)	5409.8(5)	4664.8(8)	17.3(2)
C6	2056.1(11)	5102.0(5)	4011.7(8)	18.3(2)
C7	3121.8(11)	4654.5(5)	4102.9(7)	17.3(2)
C8	3480.2(11)	4410.4(5)	3288.7(8)	18.9(2)
C9	4577.2(11)	3983.5(5)	3622.2(7)	17.9(2)
C10	4908.6(11)	3946.7(5)	4646.7(7)	17.2(2)
C11	5922.1(11)	3568.4(5)	5288.7(8)	18.0(2)
C12	6212.2(11)	3533.3(5)	6285.9(8)	17.4(2)
C13	7279.2(11)	3110.9(5)	6911.0(8)	18.0(2)
C14	7211.4(11)	3197.5(5)	7816.0(8)	18.5(2)
C15	6105.0(11)	3672.8(5)	7731.6(7)	17.7(2)
C16	5650.5(11)	3864.3(5)	8501.9(7)	18.5(2)
C17	4569.5(11)	4292.0(5)	8478.1(7)	17.9(2)
C18	3960.6(11)	4426.2(5)	9224.5(7)	18.3(2)
C19	2855.1(12)	4846.1(5)	8859.4(8)	19.2(2)
C20	2766.2(11)	4990.7(5)	7881.8(8)	17.9(2)
C21	1762.4(11)	5402.0(5)	7295.1(8)	18.5(2)
C27	5270.8(12)	3586.0(5)	3050.0(8)	20.2(2)
C28	4505.1(13)	2958.6(5)	2785.1(9)	25.3(2)
C33	4405.9(12)	4114.0(5)	10190.8(7)	20.2(2)
C34	3559.5(14)	3512.9(6)	10168.4(9)	31.5(3)

Table S3: Anisotropic Displacement Parameters (×10[4]) for phenaliporphyrin **9**. The anisotropic displacement factor exponent takes the form: $-2\pi^2[h^2a^{*2} \times U_{11} + ... + 2hka^* \times b^* \times U_{12}]$

Atom	U_{11}	U_{22}	U_{33}	U_{23}	U_{13}	U_{12}
C26	27.4(6)	27.5(6)	19.2(5)	3.2(4)	7.6(4)	1.2(4)
C29	19.5(5)	18.0(5)	22.5(5)	2.2(4)	7.7(4)	-0.3(4)
C30	28.2(6)	17.8(5)	27.0(6)	-0.3(4)	10.7(5)	-3.1(4)
C31	22.5(5)	22.8(5)	21.2(5)	6.1(4)	6.0(4)	1.7(4)
C32	22.7(6)	37.4(7)	25.2(6)	3.0(5)	1.1(5)	-1.9(5)
C35	29.2(6)	27.5(6)	23.7(5)	5.9(5)	12.5(5)	0.0(4)
C26b	27.4(6)	27.5(6)	19.2(5)	3.2(4)	7.6(4)	1.2(4)
C41b	27.6(12)	27.8(11)	19.4(11)	3.2(7)	7.7(7)	1.2(7)
C29b	19.5(5)	18.0(5)	22.5(5)	2.2(4)	7.7(4)	-0.3(4)
C31b	22.5(5)	22.8(5)	21.2(5)	6.1(4)	6.0(4)	1.7(4)
C35b	29.2(6)	27.5(6)	23.7(5)	5.9(5)	12.5(5)	0.0(4)
C42b	29.3(12)	27.6(12)	23.7(11)	5.9(7)	12.6(7)	-0.1(7)
N23	18.4(4)	16.5(4)	16.8(4)	0.4(3)	6.0(3)	-0.6(3)
N24	18.6(4)	16.7(4)	18.1(4)	0.9(3)	6.4(3)	-0.3(3)
N25	19.4(4)	18.2(4)	17.0(4)	2.2(3)	7.3(3)	0.2(3)
C22	15.9(5)	15.3(5)	21.5(5)	-0.2(4)	5.5(4)	-0.1(4)
C1	15.9(5)	14.0(5)	22.0(5)	-2.3(4)	5.9(4)	-1.5(4)
C2	16.3(5)	13.4(5)	25.5(5)	-2.4(4)	7.3(4)	-0.8(4)
C2a	21.9(5)	16.4(5)	28.1(6)	-0.7(4)	10.3(4)	-0.6(4)
C2b	23.8(5)	16.6(5)	37.0(6)	0.6(4)	14.9(5)	-1.9(4)
C2c	19.6(5)	15.8(5)	40.0(7)	2.7(4)	9.8(5)	1.5(5)
C2d	17.9(5)	15.2(5)	32.4(6)	-1.1(4)	6.3(4)	2.0(4)
C3	16.1(5)	12.7(4)	26.4(5)	-2.5(4)	5.9(4)	0.5(4)
C4	16.7(5)	13.5(5)	23.6(5)	-2.8(4)	4.3(4)	0.6(4)
C4a	21.9(5)	17.7(5)	23.8(5)	-1.3(4)	4.1(4)	0.7(4)
C4b	23.8(6)	19.9(5)	26.5(6)	-0.6(4)	0.3(4)	3.4(4)
C4c	20.3(5)	18.0(5)	34.2(6)	2.8(4)	2.3(5)	4.4(4)
C5	15.8(5)	13.8(4)	22.0(5)	-2.5(4)	5.4(4)	0.0(4)
C6	18.5(5)	17.4(5)	18.1(5)	-1.5(4)	4.4(4)	0.9(4)
C7	18.5(5)	16.1(5)	16.8(5)	-2.8(4)	5.2(4)	-0.3(4)
C8	20.5(5)	18.0(5)	18.7(5)	-2.7(4)	7.0(4)	-0.8(4)
C9	18.6(5)	17.2(5)	18.9(5)	-3.6(4)	7.4(4)	-1.8(4)
C10	17.2(5)	15.9(5)	19.6(5)	-2.8(4)	7.5(4)	-2.4(4)
C11	17.8(5)	16.3(5)	21.1(5)	-0.6(4)	8.0(4)	-1.7(4)
C12	16.4(5)	15.3(5)	20.8(5)	-0.7(4)	6.5(4)	-0.8(4)
C13	16.3(5)	15.7(5)	21.7(5)	-0.9(4)	5.5(4)	-0.7(4)
C14	17.3(5)	16.3(5)	21.2(5)	0.3(4)	5.4(4)	-0.1(4)
C15	17.1(5)	15.6(5)	19.9(5)	-0.3(4)	5.2(4)	-0.2(4)
C16	19.3(5)	18.0(5)	17.6(5)	0.1(4)	4.9(4)	1.0(4)
C17	19.2(5)	16.9(5)	17.2(5)	-1.7(4)	5.2(4)	-0.7(4)
C18	20.4(5)	16.5(5)	18.2(5)	-2.5(4)	6.5(4)	-2.6(4)
C19	21.3(5)	17.5(5)	19.6(5)	-1.5(4)	7.7(4)	-2.6(4)
C20	18.6(5)	15.8(5)	20.0(5)	-1.0(4)	7.1(4)	-2.9(4)
C21	19.2(5)	15.9(5)	21.7(5)	0.2(4)	8.3(4)	-2.6(4)
C27	20.8(5)	21.2(5)	20.2(5)	-0.6(4)	9.0(4)	-2.1(4)
C28	26.3(6)	22.5(5)	29.4(6)	-1.4(4)	12.1(5)	-5.7(4)
C33	22.6(5)	20.8(5)	17.3(5)	-0.7(4)	6.7(4)	-0.3(4)
C34	34.7(7)	30.8(6)	26.0(6)	-9.3(5)	5.6(5)	5.3(5)

Table S4: Bond Lengths in Å for phenaliporphyrin **9**.

Atom	Atom	Length/Å
C26	C8	1.4974(15)
C29	C30	1.5304(15)
C29	C13	1.5020(14)
C31	C32	1.5293(16)
C31	C14	1.5015(14)
C35	C19	1.5014(15)
C26b	C41b	1.493(17)
C26b	C8	1.4974(15)
C29b	C13	1.5020(14)
C31b	C14	1.5015(14)
C35b	C42b	1.467(17)
C35b	C19	1.5014(15)
N23	C7	1.3757(13)
N23	C10	1.3733(13)
N24	C12	1.3706(13)
N24	C15	1.3705(13)
N25	C17	1.3745(13)
N25	C20	1.3815(13)
C22	C1	1.4005(14)
C22	C5	1.4011(15)
C1	C2	1.4803(14)
C1	C21	1.4083(15)
C2	C2a	1.3902(15)
C2	C3	1.4297(15)
C2a	C2b	1.4049(15)
C2b	C2c	1.3656(17)
C2c	C2d	1.4150(16)
C2d	C3	1.4296(15)
C2d	C4c	1.4164(16)
C3	C4	1.4324(15)
C4	C4a	1.3876(15)
C4	C5	1.4813(14)
C4a	C4b	1.4061(16)
C4b	C4c	1.3644(17)
C5	C6	1.4062(15)
C6	C7	1.3908(15)
C7	C8	1.4435(14)
C8	C9	1.3748(15)
C9	C10	1.4314(14)
C9	C27	1.5005(14)
C10	C11	1.3879(15)
C11	C12	1.3969(14)
C12	C13	1.4609(14)
C13	C14	1.3600(15)
C14	C15	1.4619(14)
C15	C16	1.3989(14)
C16	C17	1.3907(15)
C17	C18	1.4299(14)
C18	C19	1.3761(15)
C18	C33	1.4985(14)
C19	C20	1.4393(14)
C20	C21	1.3926(15)
C27	C28	1.5300(15)
C33	C34	1.5277(15)

Table S5: Bond Angles in ° for phenaliporphyrin **9**.

Atom	Atom	Atom	Angle/°
C13	C29	C30	112.24(9)
C14	C31	C32	112.47(10)
C8	C26b	C41b	118.2(13)
C19	C35b	C42b	116.7(13)
C10	N23	C7	110.41(9)
C21	C1	C2	118.27(9)
C2a	C2	C1	122.65(10)
C3	C2	C1	118.87(9)
C3	C2	C2a	118.47(10)
C2b	C2a	C2	121.80(11)
C2c	C2b	C2a	120.33(11)
C2d	C2c	C2b	120.36(10)
C3	C2d	C2c	119.69(10)
C4c	C2d	C2c	120.68(10)
C4c	C2d	C3	119.63(10)
C2d	C3	C2	119.15(10)
C4	C3	C2	121.59(9)
C4	C3	C2d	119.26(10)
C4a	C4	C3	118.42(10)
C5	C4	C3	118.68(9)
C5	C4	C4a	122.90(10)
C4b	C4a	C4	121.84(11)
C4c	C4b	C4a	120.48(11)
C4b	C4c	C2d	120.28(10)
C4	C5	C22	118.08(9)
C6	C5	C22	123.48(10)
C6	C5	C4	118.40(9)
C7	C6	C5	134.36(10)
C6	C7	N23	130.86(10)
C8	C7	N23	106.25(9)
C8	C7	C6	122.89(10)
C26b	C8	C26	0.0
C7	C8	C26	124.31(10)
C7	C8	C26b	124.31(10)
C9	C8	C26	127.24(10)
C9	C8	C26b	127.24(10)
C9	C8	C7	108.42(9)
C10	C9	C8	107.34(9)
C27	C9	C8	128.35(10)
C27	C9	C10	124.20(9)
C9	C10	N23	107.57(9)
C11	C10	N23	124.49(9)
C11	C10	C9	127.94(10)
C12	C11	C10	126.96(10)
C15	N24	C12	104.84(8)
C20	N25	C17	110.49(9)
C5	C22	C1	124.62(10)
C2	C1	C22	117.89(9)
C21	C1	C22	123.71(10)
C11	C12	N24	124.93(9)
C13	C12	N24	111.46(9)
C13	C12	C11	123.60(9)
C29b	C13	C29	0.0
C12	C13	C29	125.67(9)
C12	C13	C29b	125.67(9)
C14	C13	C29	128.10(10)
C14	C13	C29b	128.10(10)
C14	C13	C12	106.14(9)
C31b	C14	C31	0.0
C13	C14	C31	128.39(10)
C13	C14	C31b	128.39(10)
C15	C14	C31	125.47(9)
C15	C14	C31b	125.47(9)
C15	C14	C13	106.13(9)
C14	C15	N24	111.41(9)
C16	C15	N24	124.91(9)
C16	C15	C14	123.58(9)
C17	C16	C15	127.41(10)
C16	C17	N25	124.91(10)
C18	C17	N25	107.35(9)
C18	C17	C16	127.67(10)
C19	C18	C17	107.52(9)
C33	C18	C17	124.39(9)
C33	C18	C19	127.92(10)
C35b	C19	C35	0.0
C18	C19	C35	126.43(10)
C18	C19	C35b	126.43(10)
C20	C19	C35	125.01(10)
C20	C19	C35b	125.01(10)
C20	C19	C18	108.54(9)
C19	C20	N25	106.08(9)
C21	C20	N25	130.09(10)
C21	C20	C19	123.83(9)
C20	C21	C1	133.93(10)
C28	C27	C9	111.88(9)
C34	C33	C18	111.53(9)

Table S6: Torsion Angles in ° for phenaliporphyrin **9.**

Atom	Atom	Atom	Atom	Angle/°
C26	C8	C7	N23	177.74(11)
C26	C8	C7	C6	-2.19(13)
C26	C8	C9	C10	-177.42(12)
C26	C8	C9	C27	-1.20(14)
C29	C13	C12	N24	177.60(11)
C29	C13	C12	C11	-1.16(12)
C29	C13	C14	C31	4.11(14)
C29	C13	C14	C31b	4.11(14)
C29	C13	C14	C15	-176.77(11)
C30	C29	C13	C12	-83.42(10)
C30	C29	C13	C14	92.64(10)
C31	C14	C13	C29b	4.11(14)
C31	C14	C13	C12	-179.22(12)
C31	C14	C15	N24	178.50(11)
C31	C14	C15	C16	-4.96(13)
C32	C31	C14	C13	98.37(11)
C32	C31	C14	C15	-80.59(11)
C35	C19	C18	C17	177.84(12)
C35	C19	C18	C33	2.49(14)
C35	C19	C20	N25	-178.77(11)
C35	C19	C20	C21	1.23(13)
C26b	C8	C7	N23	177.74(11)
C26b	C8	C7	C6	-2.19(13)
C26b	C8	C9	C10	-177.42(12)
C26b	C8	C9	C27	-1.20(14)
C41b	C26b	C8	C7	-103.3(15)
C41b	C26b	C8	C9	74.7(15)
C29b	C13	C12	N24	177.60(11)
C29b	C13	C12	C11	-1.16(12)
C29b	C13	C14	C31b	4.11(14)
C29b	C13	C14	C15	-176.77(11)
C31b	C14	C13	C12	-179.22(12)
C31b	C14	C15	N24	178.50(11)
C31b	C14	C15	C16	-4.96(13)
C35b	C19	C18	C17	177.84(12)
C35b	C19	C18	C33	2.49(14)
C35b	C19	C20	N25	-178.77(11)
C35b	C19	C20	C21	1.23(13)
C42b	C35b	C19	C18	-74.5(15)
C42b	C35b	C19	C20	104.1(15)
N23	C7	C6	C5	-0.21(15)
N23	C7	C8	C9	-0.57(9)
N23	C10	C9	C8	-0.78(9)
N23	C10	C9	C27	-177.19(8)
N23	C10	C11	C12	0.34(13)
N24	C12	C11	C10	-0.02(13)
N24	C12	C13	C14	0.83(9)
N24	C15	C14	C13	-0.65(10)
N24	C15	C16	C17	-1.07(13)
N25	C17	C16	C15	-6.23(13)
N25	C17	C18	C19	1.50(9)
N25	C17	C18	C33	177.05(8)
N25	C20	C19	C18	0.02(9)
N25	C20	C21	C1	5.73(15)
C22	C1	C2	C2a	-175.27(9)
C22	C1	C2	C3	5.74(11)
C22	C1	C21	C20	8.10(14)
C22	C5	C4	C3	1.74(11)
C22	C5	C4	C4a	-177.35(9)
C22	C5	C6	C7	-2.97(14)
C1	C22	C5	C4	-1.50(12)
C1	C22	C5	C6	-179.21(10)

C1	C2	C2a	C2b	-174.59(10)
C1	C2	C3	C2d	174.26(9)
C1	C2	C3	C4	-5.61(11)
C1	C21	C20	C19	-174.27(12)
C2	C1	C22	C5	-2.24(11)
C2	C1	C21	C20	-176.05(9)
C2	C2a	C2b	C2c	-0.59(13)
C2	C3	C2d	C2c	1.51(11)
C2	C3	C2d	C4c	-178.32(10)
C2	C3	C4	C4a	-179.01(10)
C2	C3	C4	C5	1.86(11)
C2a	C2	C1	C21	8.64(12)
C2a	C2	C3	C2d	-4.77(11)
C2a	C2	C3	C4	175.35(9)
C2a	C2b	C2c	C2d	-2.85(13)
C2b	C2a	C2	C3	4.41(12)
C2b	C2c	C2d	C3	2.34(13)
C2b	C2c	C2d	C4c	-177.83(11)
C2c	C2d	C3	C4	-178.61(10)
C2c	C2d	C4c	C4b	177.40(11)
C2d	C3	C4	C4a	1.12(11)
C2d	C3	C4	C5	-178.02(9)
C2d	C4c	C4b	C4a	1.27(13)
C3	C2	C1	C21	-170.36(9)
C3	C2d	C4c	C4b	-2.78(12)
C3	C4	C4a	C4b	-2.70(12)
C3	C4	C5	C6	179.57(9)
C4	C3	C2d	C4c	1.56(11)
C4	C4a	C4b	C4c	1.54(13)
C4	C5	C6	C7	179.32(9)
C4a	C4	C5	C6	0.48(12)
C4b	C4a	C4	C5	176.40(10)
C5	C22	C1	C21	173.62(10)
C5	C6	C7	C8	179.69(12)
C6	C7	N23	C10	179.99(12)
C6	C7	C8	C9	179.50(11)
C7	N23	C10	C9	0.43(9)
C7	N23	C10	C11	-178.97(8)
C7	C8	C9	C10	0.82(9)
C7	C8	C9	C27	177.04(8)
C8	C7	N23	C10	0.07(9)
C8	C9	C10	C11	178.59(8)
C8	C9	C27	C28	-90.45(12)
C9	C10	C11	C12	-178.93(10)
C10	C9	C27	C28	85.18(11)
C10	C11	C12	C13	178.58(10)
C11	C10	C9	C27	2.18(13)
C11	C12	N24	C15	177.54(11)
C11	C12	C13	C14	-177.93(11)
C12	N24	C15	C14	1.13(9)
C12	N24	C15	C16	-175.35(8)
C12	C13	C14	C15	-0.10(9)
C13	C12	N24	C15	-1.19(9)
C13	C14	C15	C16	175.88(8)
C14	C15	C16	C17	-177.13(10)
C15	C16	C17	C18	170.47(11)
C16	C17	N25	C20	175.74(11)
C16	C17	C18	C19	-175.67(12)
C16	C17	C18	C33	-0.12(13)
C17	N25	C20	C19	0.95(9)
C17	N25	C20	C21	-179.05(8)
C17	C18	C19	C20	-0.92(9)
C17	C18	C33	C34	-86.98(12)
C18	C17	N25	C20	-1.52(10)
C18	C19	C20	C21	-179.98(8)
C19	C18	C33	C34	87.65(12)
C20	C19	C18	C33	-176.27(8)

Table S7: Hydrogen Fractional Atomic Coordinates (×10^4) and Equivalent Isotropic Displacement Parameters (Å^2×10^3) for phenaliporphyrin **9**. U_{eq} is defined as 1/3 of the trace of the orthogonalised U_{ij}.

**Atom**	**x**	**y**	**z**	U_{eq}
H26a	1689(2)	4502(4)	2098(2)	37.0(4)
H26b	2868(9)	5035.5(11)	2178.4(17)	37.0(4)
H26c	3127(7)	4351(3)	1837.0(9)	37.0(4)
H29a	9078.2(11)	2562.5(5)	7126.9(8)	23.8(3)
H29b	8479.8(11)	2839.1(5)	6056.8(8)	23.8(3)
H30a	6487(5)	2129.8(7)	5705(5)	35.8(4)
H30b	7997(4)	1765.3(18)	5998(6)	35.8(4)
H30c	7148(9)	1841(2)	6763.4(19)	35.8(4)
H31a	8462.0(12)	2485.4(5)	8580.3(8)	26.9(3)
H31b	7455.2(12)	2795.6(5)	9129.8(8)	26.9(3)
H32a	10010(6)	3366(4)	8947(3)	45.0(4)
H32b	8997.4(15)	3683(2)	9487(6)	45.0(4)
H32c	9892(7)	3067(2)	9920(4)	45.0(4)
H35a	917(3)	4912(3)	9118(5)	38.7(4)
H35b	2280(6)	5016(4)	10065.5(13)	38.7(4)
H35c	1784(8)	5555.9(9)	9276(6)	38.7(4)
H26d	3475.7(13)	4670.7(6)	1953.4(8)	29.6(3)
H26e	2217.6(13)	4988.8(6)	2264.4(8)	29.6(3)
H41a	740(90)	4180(90)	1770(130)	37.3(10)
H41b	1500(200)	4250(80)	960(20)	37.3(10)
H41c	2030(130)	3720(18)	1770(140)	37.3(10)
H29c	7580(60)	2300(60)	6260(160)	29.7(3)
H29d	8600(200)	2860(30)	6140(140)	29.7(3)
H29e	8960(160)	2500(90)	7145(19)	29.7(3)
H31c	7793.0(12)	3045.5(5)	9272.0(8)	33.6(3)
H31d	7927.8(12)	2435.9(5)	8678.4(8)	33.6(3)
H31e	9117.6(12)	2974.7(5)	8857.7(8)	33.6(3)
H35d	1392.1(13)	5479.2(6)	9021.0(8)	31.0(3)
H35e	2480.5(13)	5241.9(6)	10023.7(8)	31.0(3)
H42a	190(190)	4520(100)	8860(40)	38.9(10)
H42b	1210(30)	4350(70)	9920(160)	38.9(10)
H42c	110(180)	4920(30)	9760(180)	38.9(10)
H23	4076(14)	4400(6)	5552(10)	20.6(2)
H25	4020(14)	4607(6)	7123(10)	21.4(2)
H22	2765(14)	4957(6)	5911(9)	21.2(2)
H2a	136(15)	6387(6)	7267(10)	25.9(3)
H2b	-1707.7(12)	7088.3(5)	6631.5(9)	29.5(3)
H2c	-2735.7(12)	7225.7(5)	4987.0(9)	30.1(3)
H4a	114(14)	5840(6)	2812(10)	26.3(3)
H4b	-1760.5(13)	6519.3(5)	2283.7(9)	30.3(3)
H4c	-2757.0(12)	7008.3(5)	3327.3(9)	30.8(3)
H6	1663(13)	5208(6)	3348(10)	22.0(2)
H11	6478.0(11)	3305.3(5)	5021.5(8)	21.6(2)
H16	6135.6(11)	3681.3(5)	9110.7(7)	22.3(2)
H21	1188(14)	5579(6)	7662(10)	22.2(2)
H27a	5245.1(12)	3808.5(5)	2452.4(8)	24.2(3)
H27b	6297.6(12)	3514.4(5)	3431.0(8)	24.2(3)
H28a	3491(3)	3027.5(5)	2401(6)	38.0(4)
H28b	4983(6)	2714.1(18)	2409(6)	38.0(4)
H28c	4551(9)	2732.2(19)	3375.4(9)	38.0(4)
H33a	5453.5(12)	4018.6(5)	10390.0(7)	24.2(3)
H33b	4239.2(12)	4400.9(5)	10673.9(7)	24.2(3)
H34a	3699(9)	3233.3(19)	9677(5)	47.2(4)
H34b	3909(7)	3310(2)	10801(2)	47.2(4)
H34c	2528(2)	3609.6(8)	10014(7)	47.2(4)

Table S8: Atomic Occupancies for all atoms that are not fully occupied

Atom	Occupancy	Atom	Occupancy	Atom	Occupancy	Atom	Occupancy
C26	0.9652(19)	C31	0.9652(19)	C26b	0.0348(19)	C31b	0.0348(19)
H26a	0.9652(19)	H31a	0.9652(19)	H26d	0.0348(19)	H31c	0.0348(19)
H26b	0.9652(19)	H31b	0.9652(19)	H26e	0.0348(19)	H31d	0.0348(19)
H26c	0.9652(19)	C32	0.9652(19)	C41b	0.0348(19)	H31e	0.0348(19)
C29	0.9652(19)	H32a	0.9652(19)	H41a	0.0348(19)	C35b	0.0348(19)
H29a	0.9652(19)	H32b	0.9652(19)	H41b	0.0348(19)	H35d	0.0348(19)
H29b	0.9652(19)	H32c	0.9652(19)	H41c	0.0348(19)	H35e	0.0348(19)
C30	0.9652(19)	C35	0.9652(19)	C29b	0.0348(19)	C42b	0.0348(19)
H30a	0.9652(19)	H35a	0.9652(19)	H29c	0.0348(19)	H42a	0.0348(19)
H30b	0.9652(19)	H35b	0.9652(19)	H29d	0.0348(19)	H42b	0.0348(19)
H30c	0.9652(19)	H35c	0.9652(19)	H29e	0.0348(19)	H42c	0.0348(19)

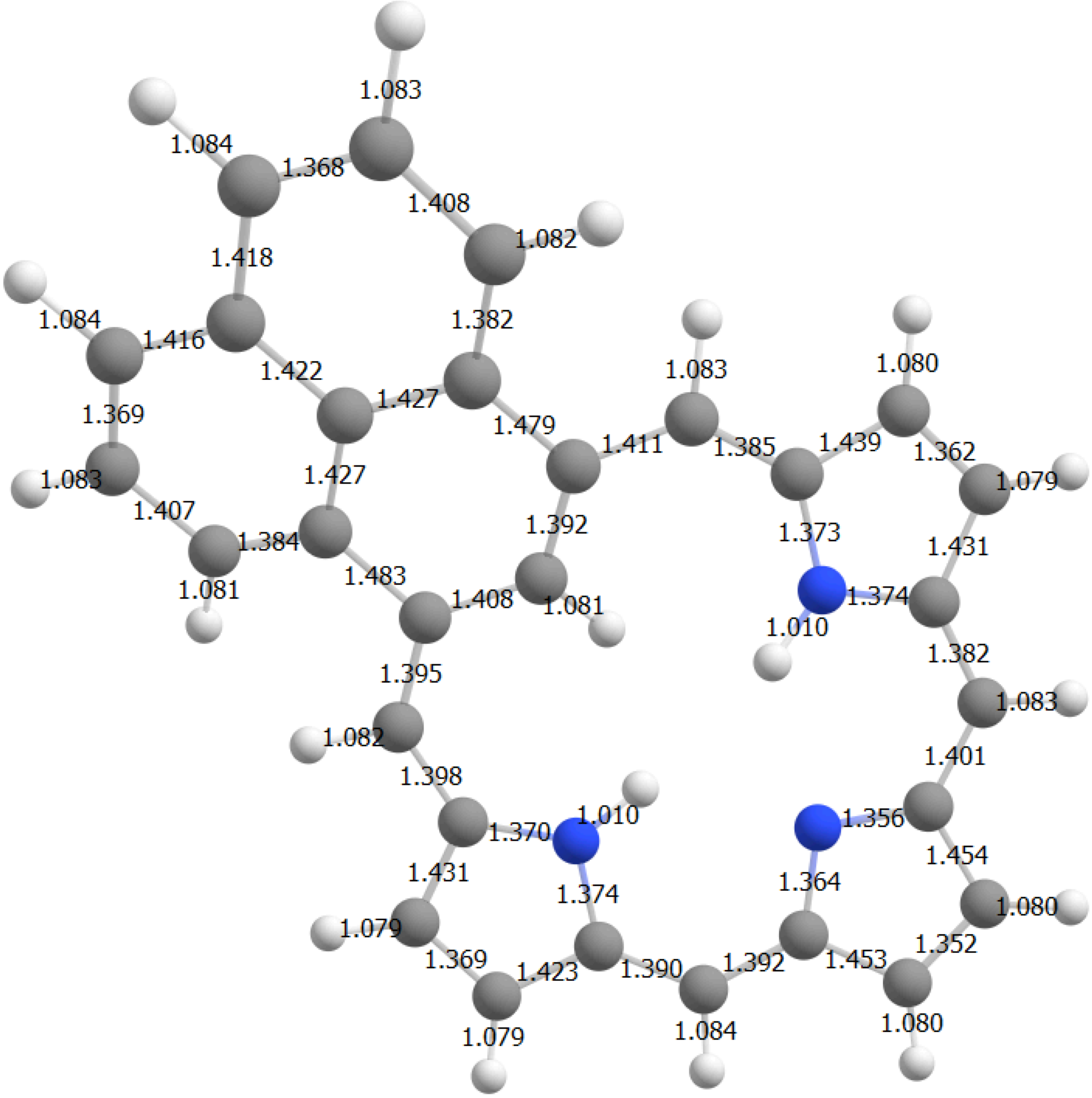

Figure S49. Calculated bond lengths for phenaliporphyrin tautomer **PhP-23,25-H**.

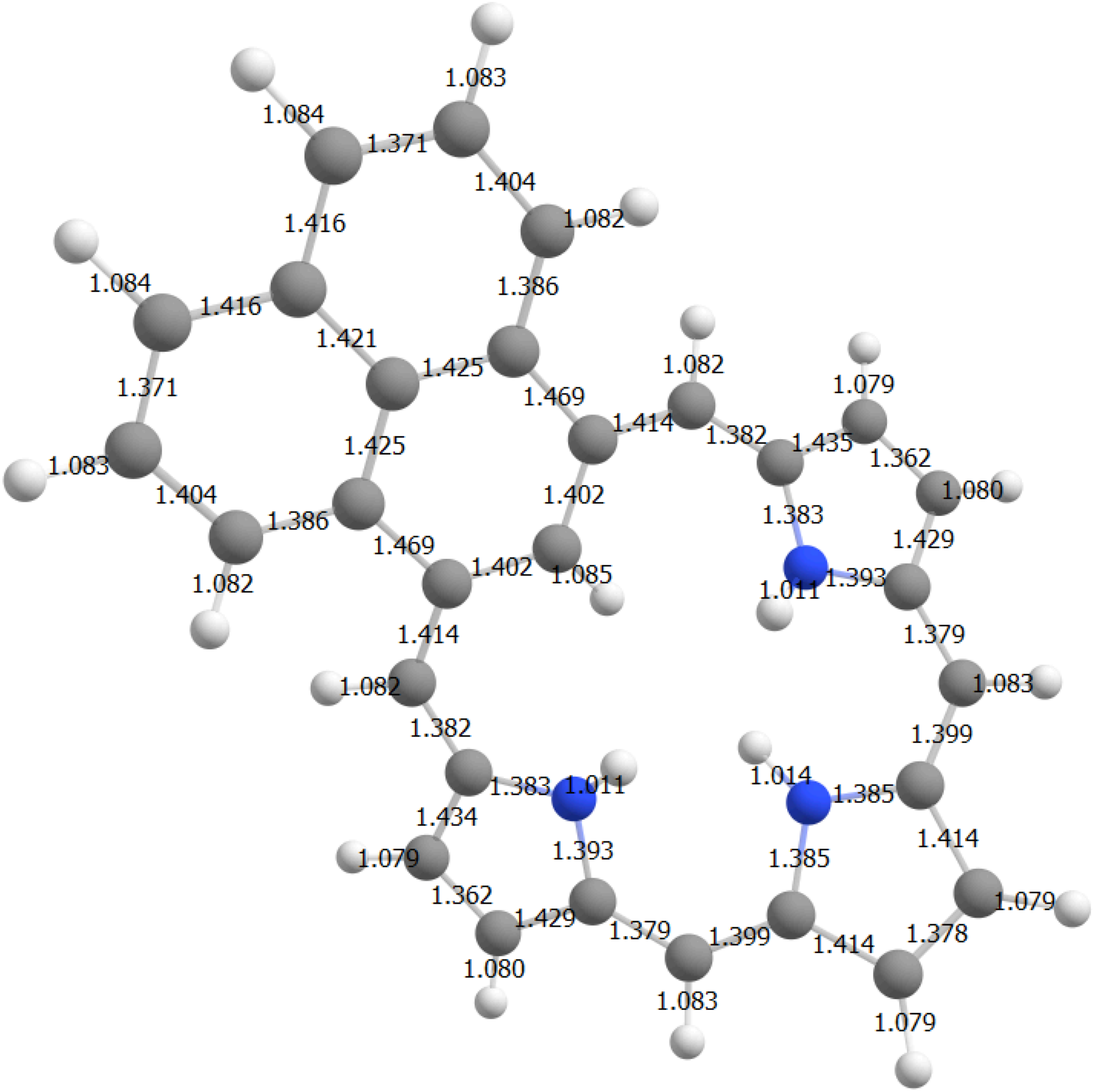

Figure S50. Calculated bond lengths for phenaliporphyrin monocation **PhPaH^{+}**.

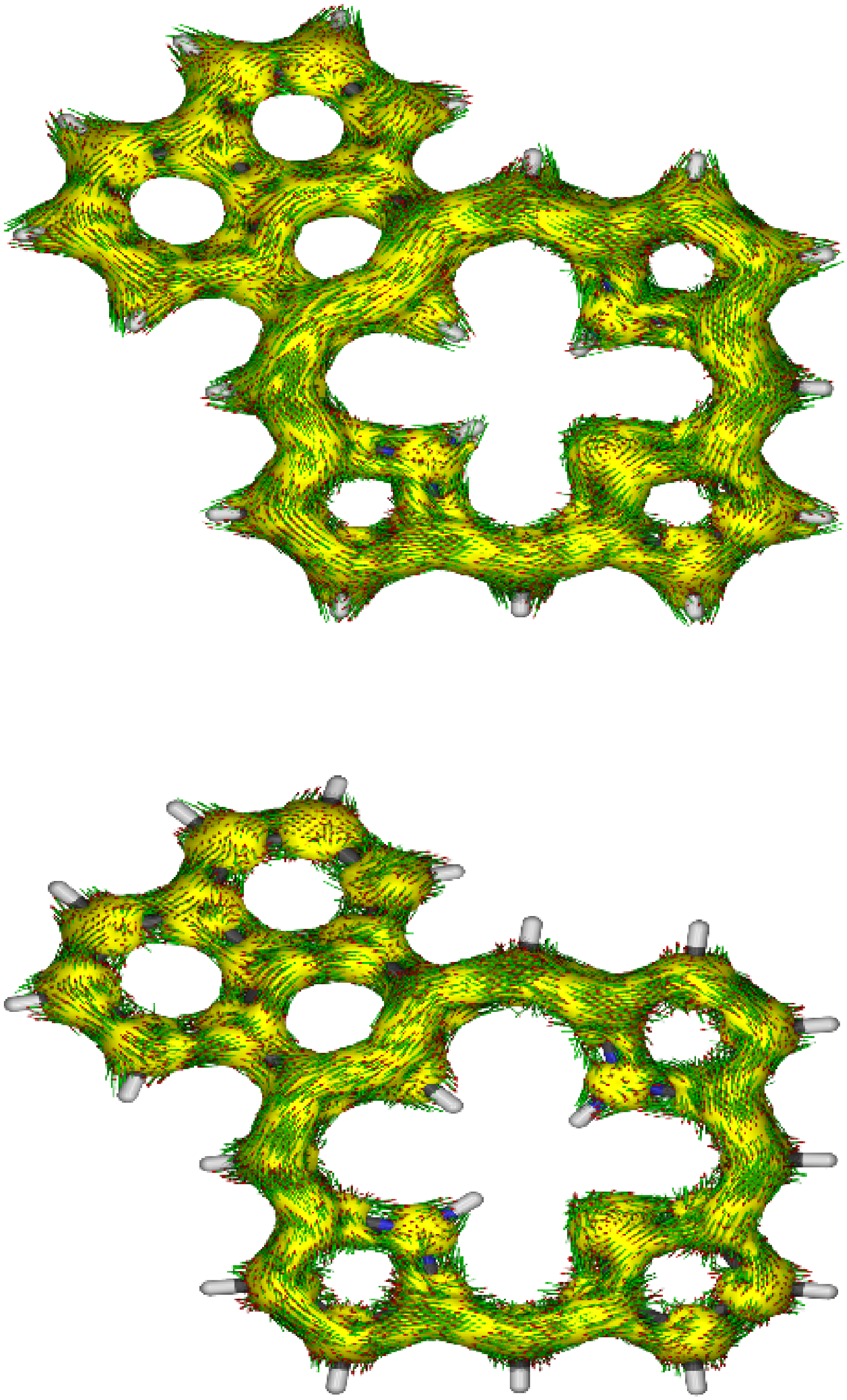

Figure S51. AICD plots for phenaliporphyrin tautomer **PhP-23,25-H** (isovalues 0.05 and 0.07, respectively).

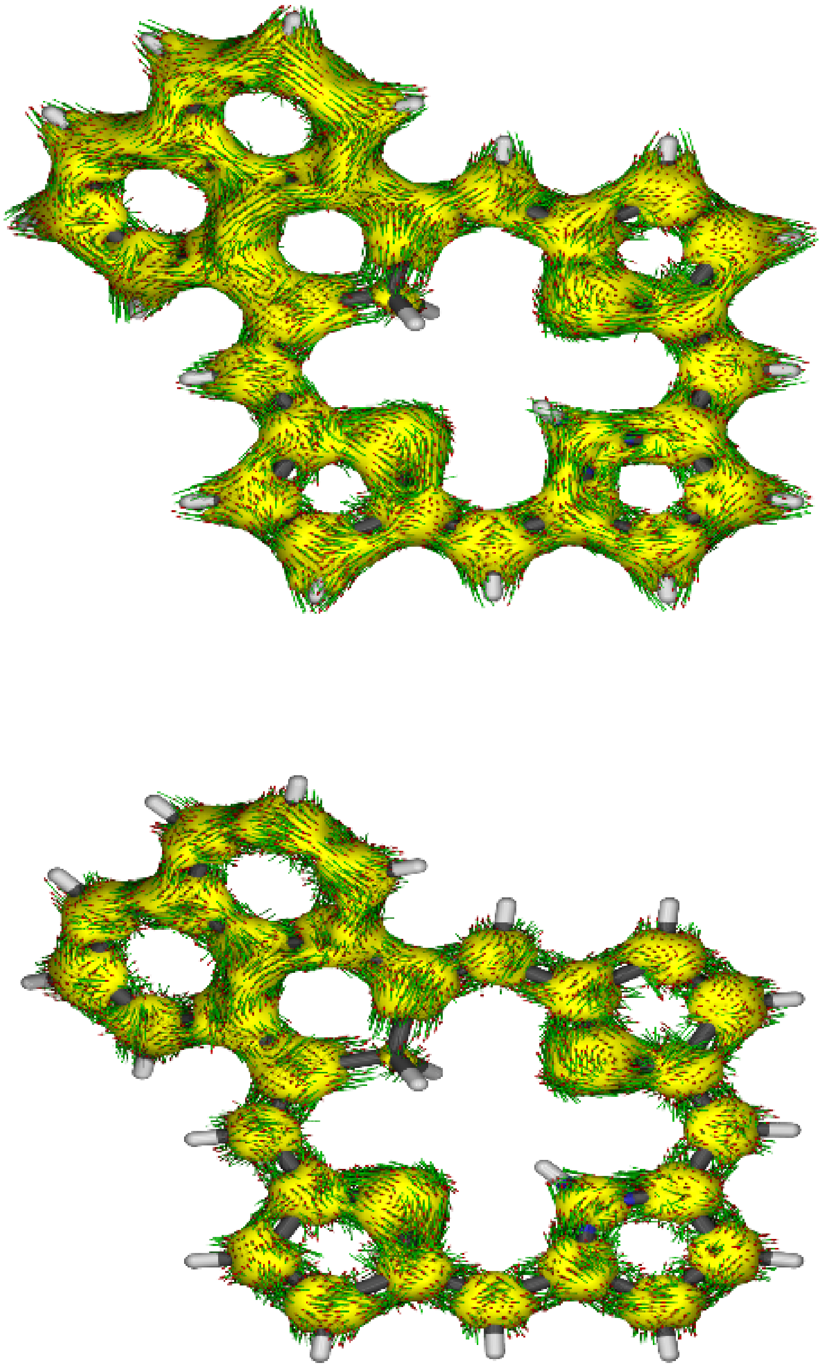

Figure S52. AICD plots for phenaliporphyrin tautomer **PhP-22,24-H** (isovalues 0.05 and 0.07, respectively).

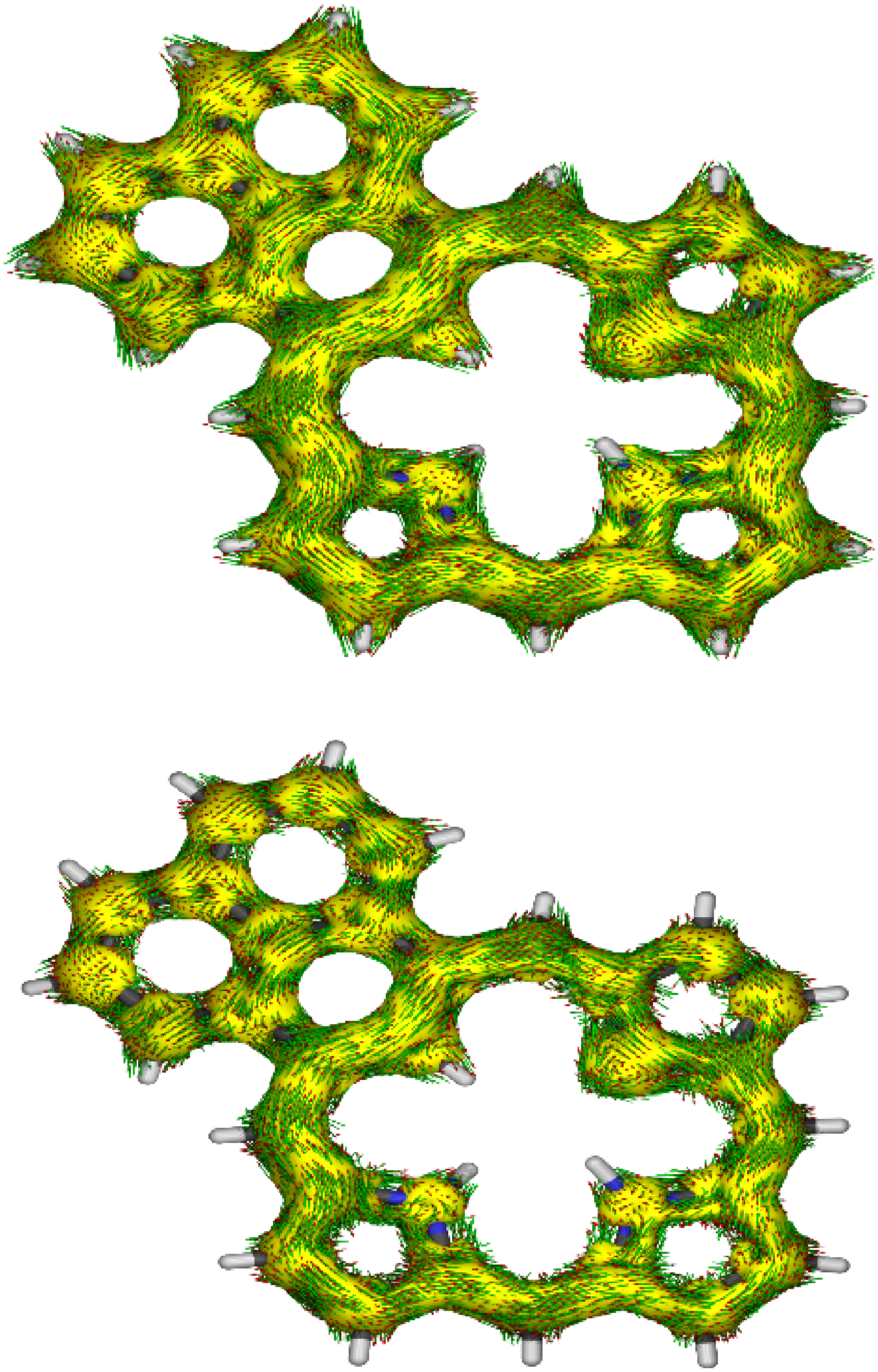

Figure S53. AICD plots for phenaliporphyrin tautomer **PhP-23,24-H** (isovalues 0.05 and 0.07, respectively).

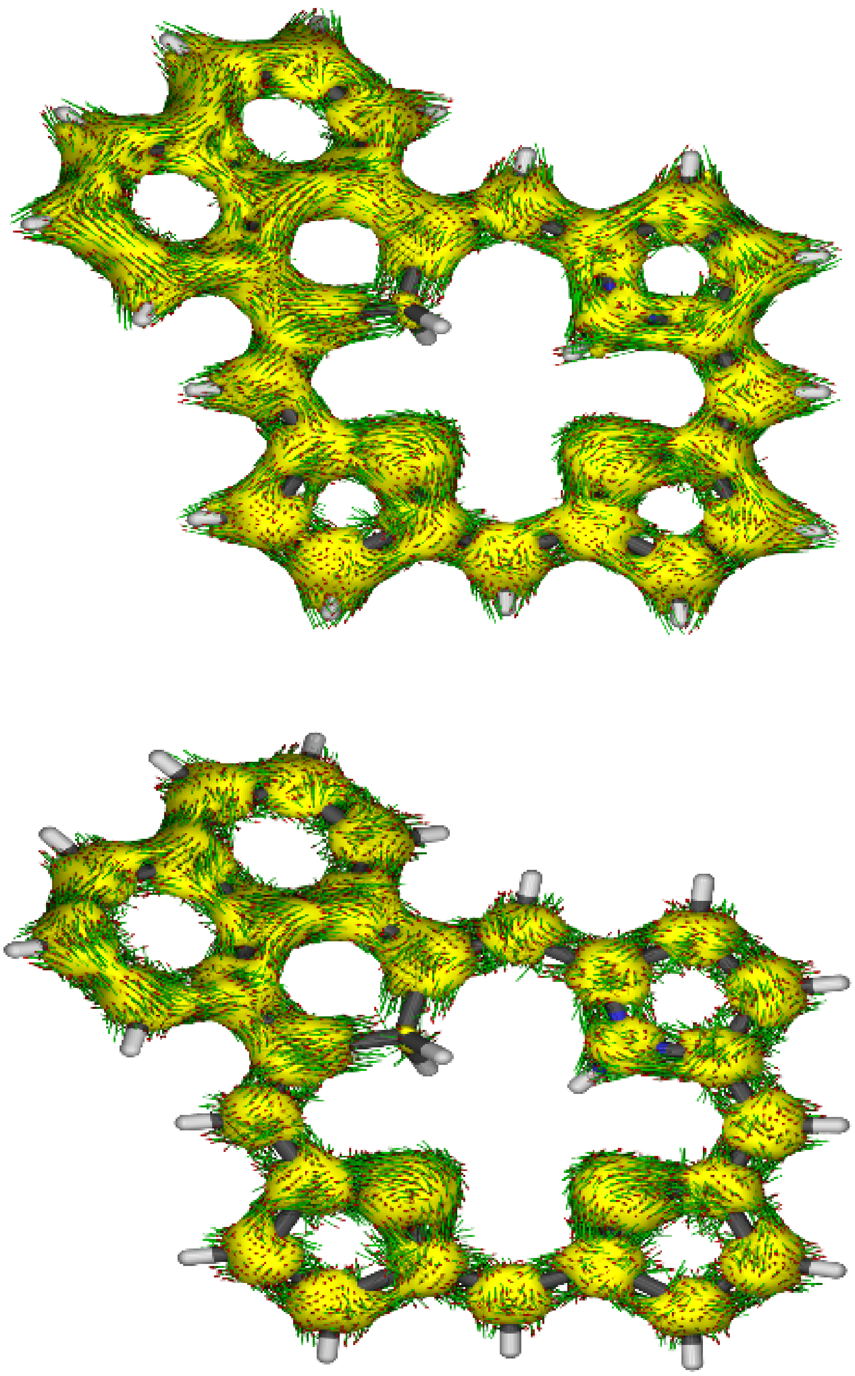

Figure S54. AICD plots for phenaliporphyrin tautomer **PhP-22,23-H** (isovalues 0.05 and 0.07, respectively).

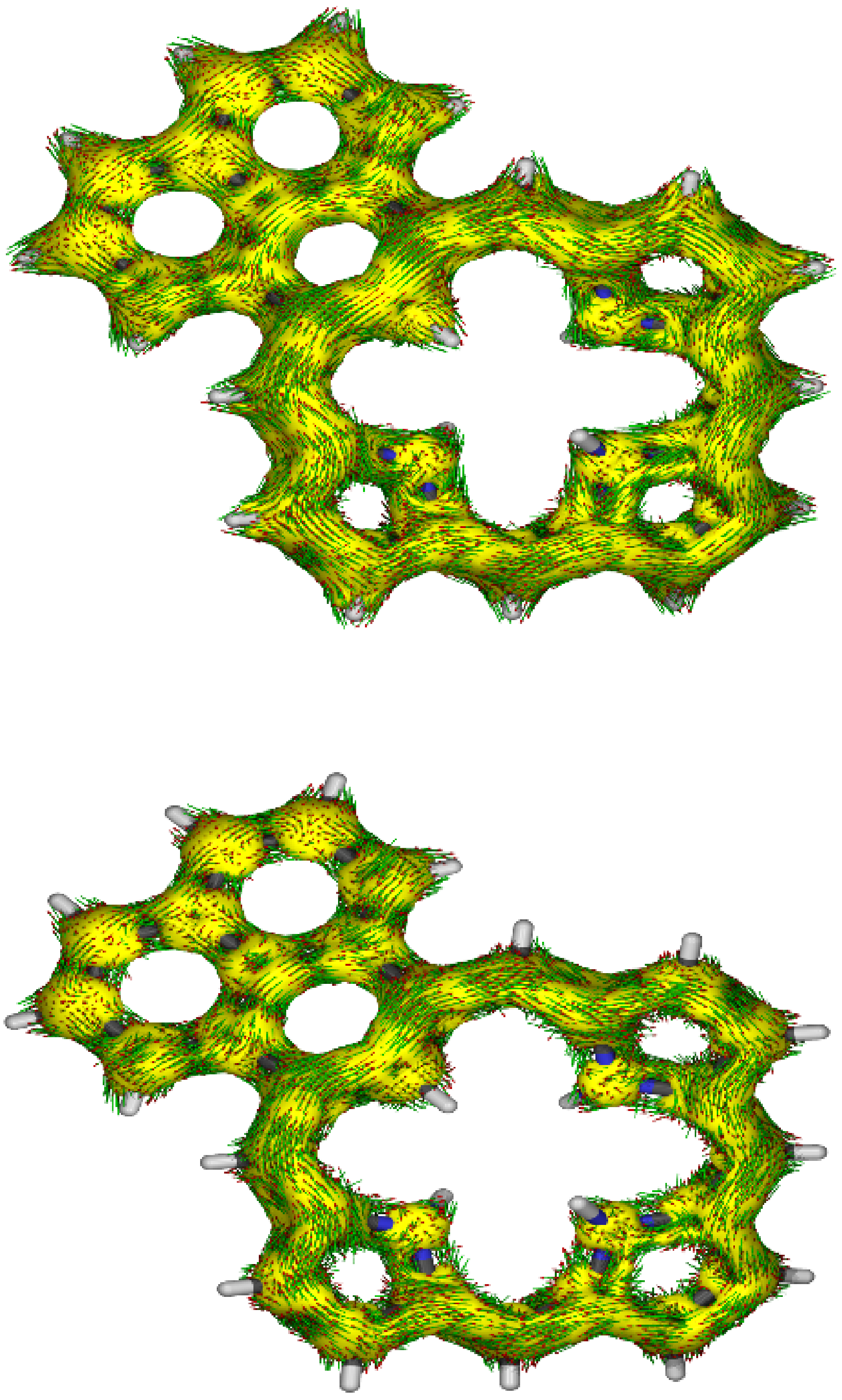

Figure S55. AICD plots for phenaliporphyrin cation **PhPaH$^+$** (isovalues 0.05 and 0.07, respectively).

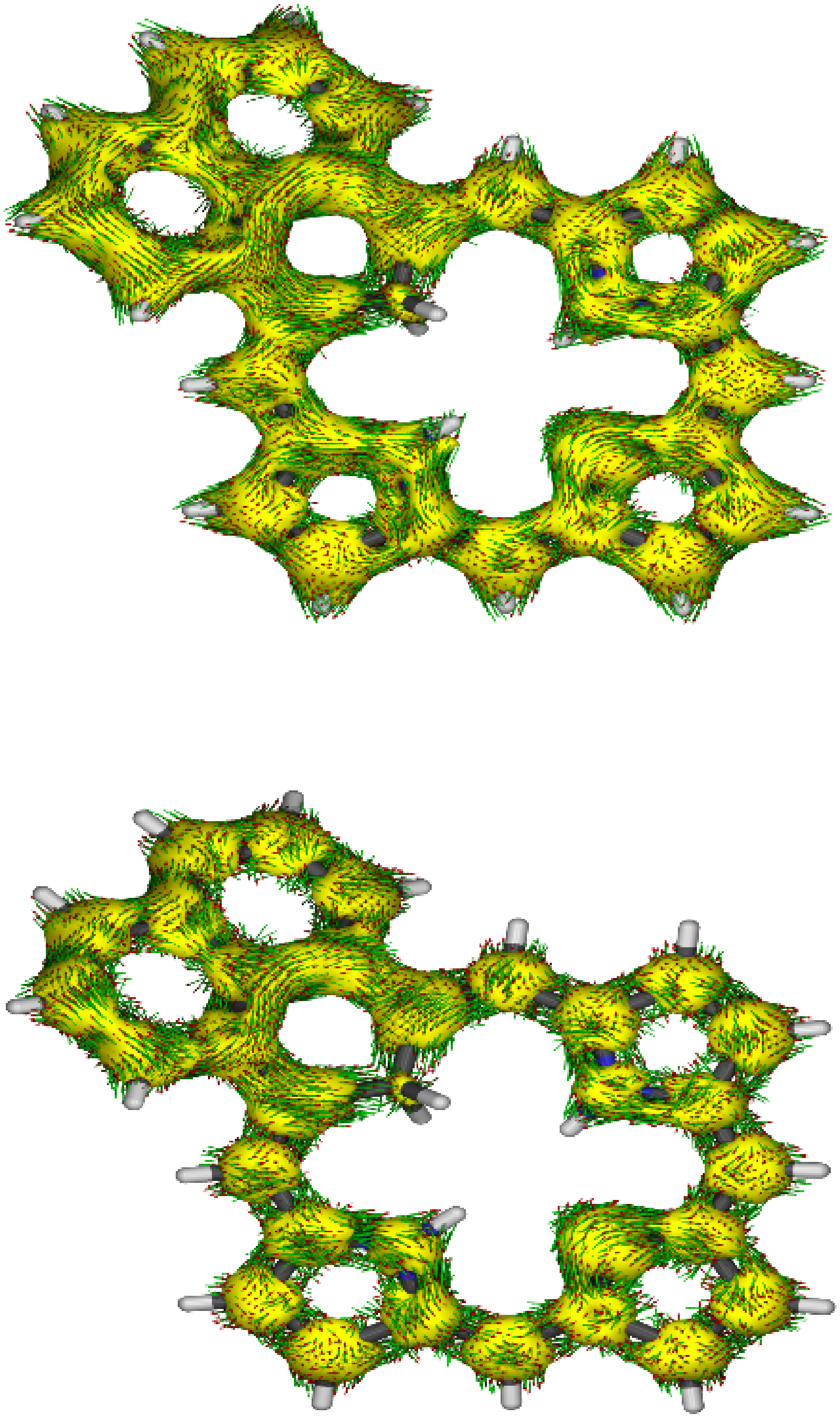

Figure S56. AICD plots for phenaliporphyrin cation **PhPbH**$^{+}$ (isovalues 0.05 and 0.07, respectively).

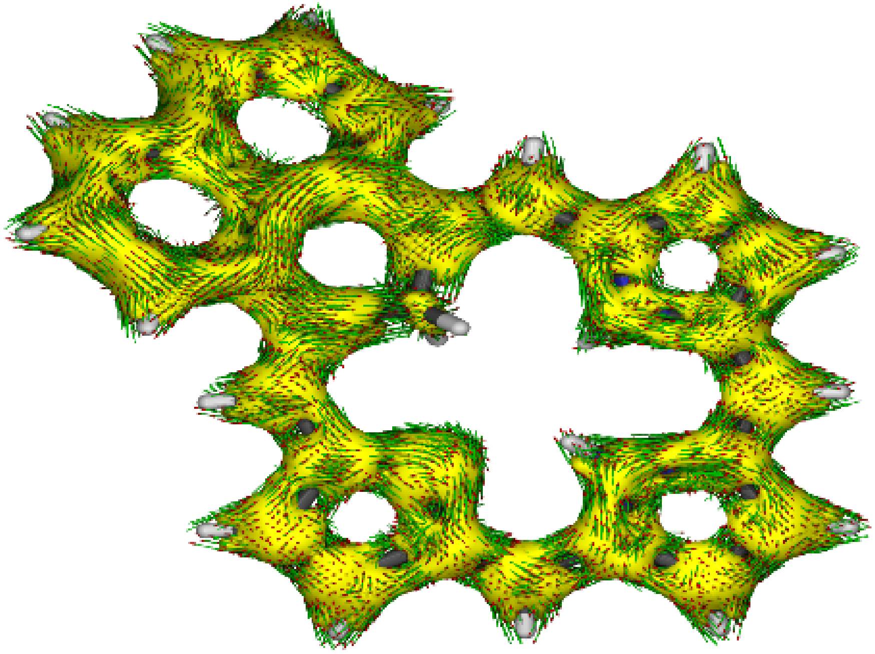

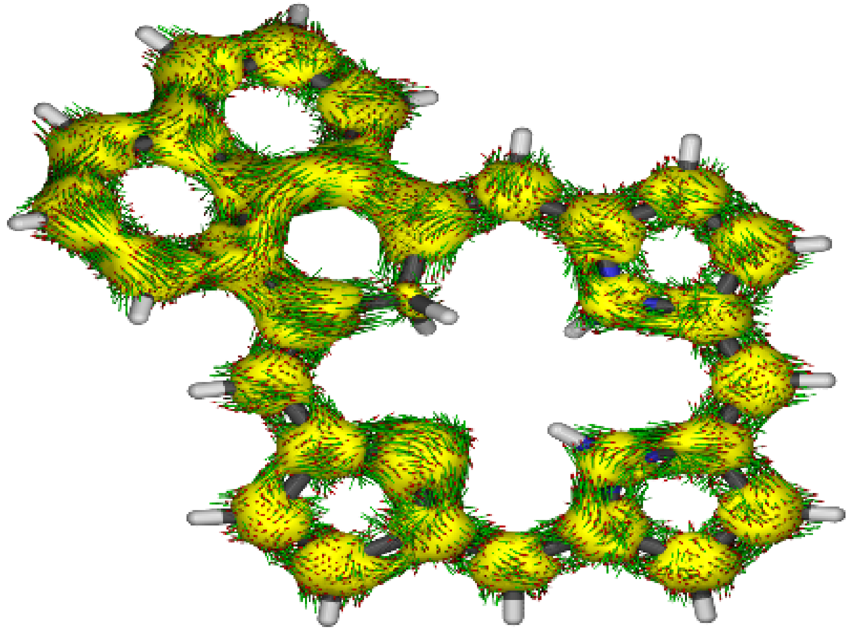

Figure S57. AICD plots for phenaliporphyrin cation **PhPcH**$^+$ (isovalues 0.05 and 0.07, respectively).

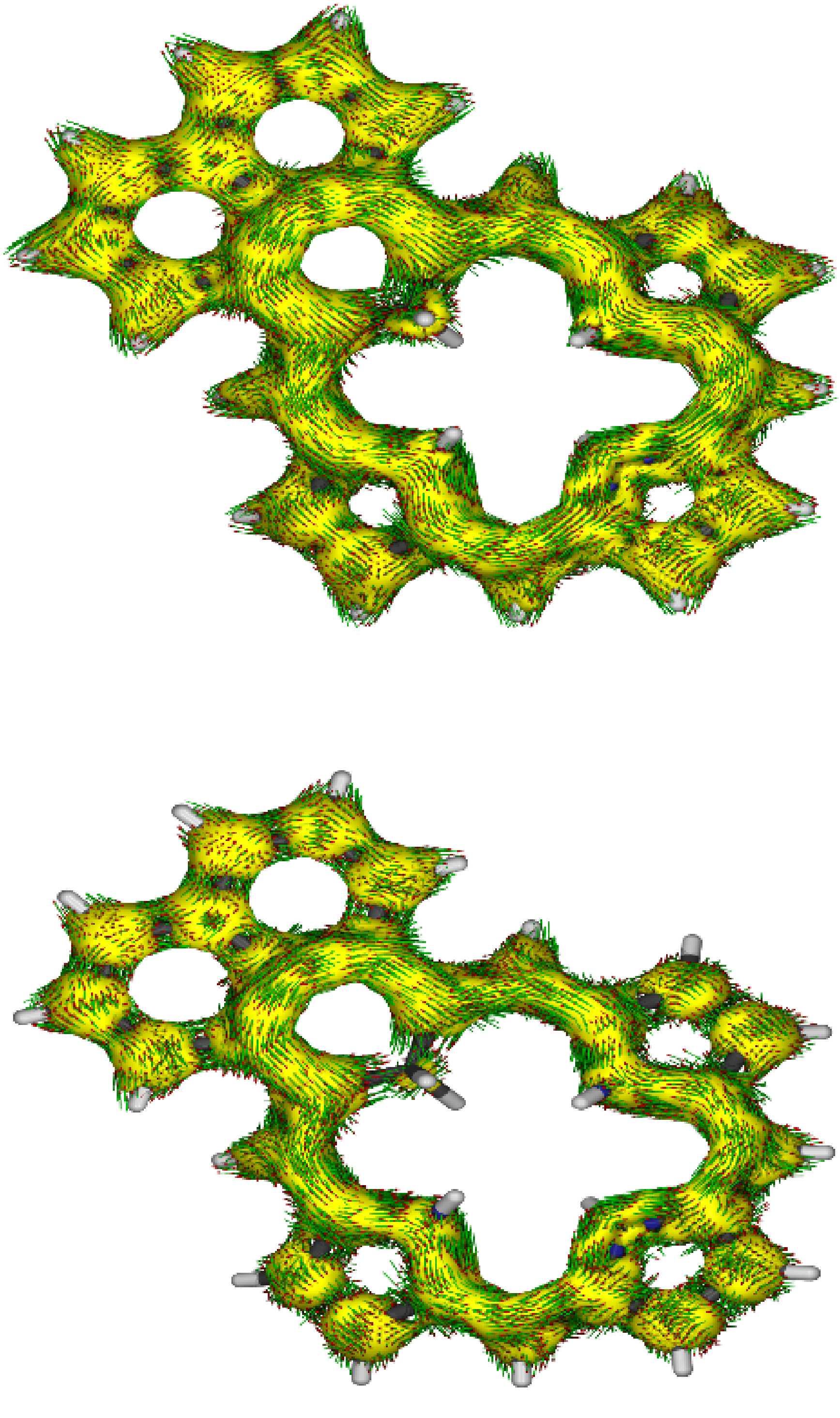

Figure S58. AICD plots for phenaliporphyrin dication **PhPaH$_2$$^{2+}$** (isovalues 0.05 and 0.07, respectively).

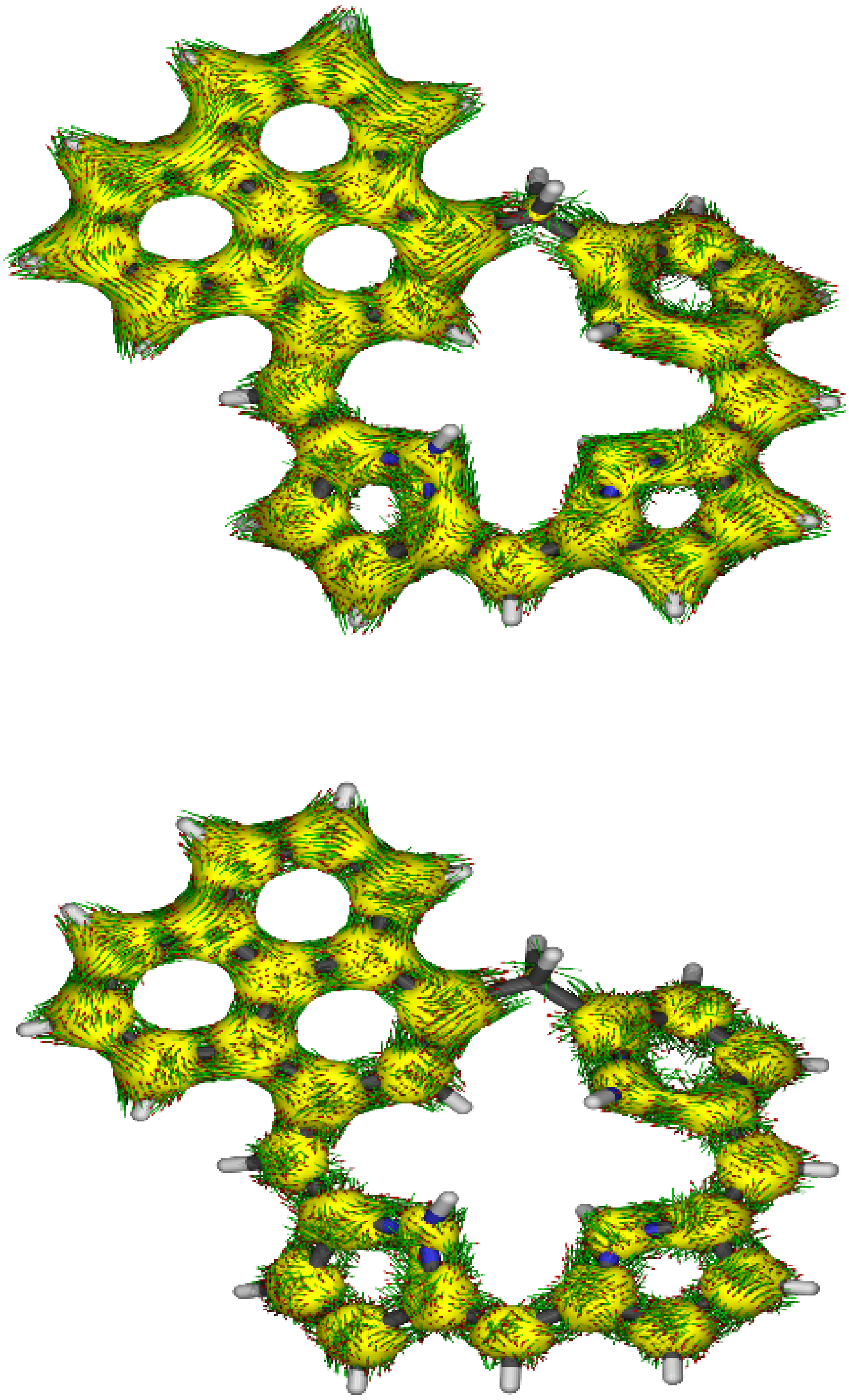

Figure S59. AICD plots for phenaliporphyrin dication **PhPbH$_2^{2+}$** (isovalues 0.05 and 0.07, respectively).

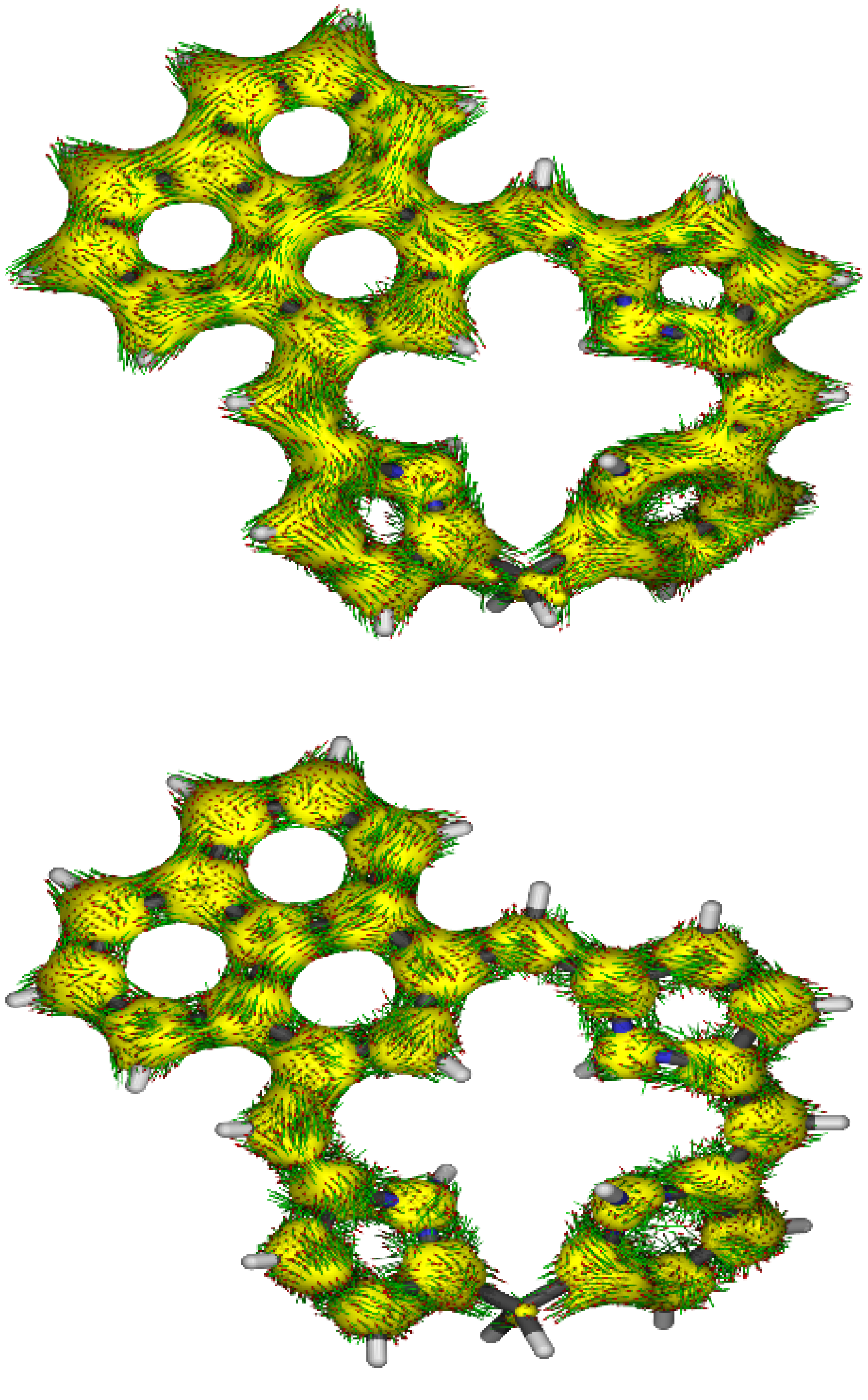

Figure S60. AICD plots for phenaliporphyrin dication **$PhPcH_2^{2+}$** (isovalues 0.05 and 0.07, respectively).

Table S9. Calculated Gibb's free energies (kcal/mol) and electronic energies (kcal/mol) for phenaliporphyrin tautomers and related protonated species. Free energies were calculated using M06-2X/6-311++G(dip). Electronic energies calculated using M06-2X/cc-PVTZ.

TOCP (Relative Energy Analysis)				
Molecule	E (Hartrees)	G (Hartrees)	Delta E (kcal/mol)	Delta G (kcal/mol)
	M06-2X cc-PVTZ	M06-2X 6-311++G(d,p)	M06-2X cc-PVTZ	M06-2X 6-311++G(d,p)
**PhP-23,25-H**	-1280.695	-1280.2144	0.00	0.00
**PhP-23,24-H**	-1280.6891	--1280.2083	3.67	3.87
**PhP-22,24-H**	-1280.6692	-1280.191	16.16	14.68
**PhP-22,23-H**	-1280.6573	-1280.1791	23.67	22.20
**$PhPaH^+$**	-1281.0943	-1280.5974	0.00	0.00
**$PhPbH^+$**	-1281.0714	-1280.5572	14.36	12.62
**$PhPcH^+$**	-1281.0676	-1280.5732	16.74	15.14
**$PhPaH_2^{2+}$**	-1281.3427	-1280.8351	10.58	10.67
**$PhPbH_2^{2+}$**	-1281.3596	-1280.8521	0.00	0.00
**$PhPcH_2^{2+}$**	-1281.3457	-1280.8394	8.69	7.99

Table S10. Cartesian coordinates for phenaliporphyrin tautomers, and related monocations and dications.

PhP-23,25-H

C	-5.807385000	-0.701288000	-0.403876000
C	-5.823820000	0.644296000	-0.269657000
C	-4.438280000	1.052721000	-0.108007000
N	-3.604149000	-0.015062000	-0.147682000
C	-4.413872000	-1.100825000	-0.312551000
C	-0.887544000	-4.212034000	0.040294000
C	-2.243040000	-4.242139000	-0.146131000
C	-2.703431000	-2.896468000	-0.176590000
N	-1.602474000	-2.092840000	-0.005200000
C	-0.466634000	-2.847301000	0.126259000
C	-4.003494000	-2.430442000	-0.334249000
C	1.516309000	-1.233503000	0.273990000
C	1.469579000	1.227835000	0.092504000
C	0.873593000	-2.471743000	0.260803000
C	-2.325884000	4.188017000	0.501542000
C	-0.964693000	4.176380000	0.552207000
C	-0.505508000	2.832488000	0.322876000
N	-1.636742000	2.072599000	0.156728000
C	-2.765998000	2.850818000	0.242761000
C	-4.058614000	2.386418000	0.092715000
C	0.830981000	2.475957000	0.255553000
C	0.838270000	0.000979000	0.272998000
C	2.912543000	1.226853000	-0.229840000
C	3.655660000	0.022808000	-0.043183000
C	2.998560000	-1.202310000	0.280235000
C	3.570871000	2.349903000	-0.694199000
C	4.965201000	2.355708000	-0.892829000
C	5.706208000	1.238360000	-0.619809000
C	5.069766000	0.046111000	-0.191532000
C	5.818063000	-1.123663000	0.083185000
C	5.180696000	-2.267625000	0.482911000
C	3.777311000	-2.308299000	0.570837000
H	-6.639930000	-1.376505000	-0.538056000
H	-6.672506000	1.312630000	-0.272978000
H	-0.211587000	-5.050754000	0.108796000
H	-2.878785000	-5.106982000	-0.256783000
H	-1.718052000	-1.089169000	-0.000923000
H	-4.770443000	-3.185547000	-0.461670000
H	1.511132000	-3.345217000	0.276154000
H	-2.988098000	5.030173000	0.631427000
H	-0.302030000	5.009421000	0.731833000
H	-1.717306000	1.103352000	-0.117136000
H	-4.848273000	3.126200000	0.147389000
H	1.483373000	3.334156000	0.355787000
H	-0.210625000	-0.001274000	0.533779000
H	3.016041000	3.249783000	-0.924697000
H	5.446085000	3.257149000	-1.253334000
H	6.783607000	1.240335000	-0.742313000
H	6.896895000	-1.093138000	-0.020882000
H	5.751259000	-3.157760000	0.719508000
H	3.320316000	-3.237799000	0.881879000

PhP-23,24-H

C	-5.811728000	-0.681398000	-0.391861000
C	-5.836442000	0.688081000	-0.479203000
C	-4.508312000	1.158539000	-0.289381000
N	-3.713721000	0.055013000	-0.109227000
C	-4.467591000	-1.088723000	-0.149155000
C	-0.932237000	-4.150874000	0.652692000
C	-2.293235000	-4.142306000	0.574107000
C	-2.713644000	-2.827865000	0.202999000
N	-1.562521000	-2.065857000	0.083268000
C	-0.445412000	-2.844350000	0.324775000
C	-4.015515000	-2.396642000	0.037487000
C	1.506389000	-1.232197000	0.065198000
C	1.509103000	1.225473000	0.221477000
C	0.887343000	-2.488519000	0.257284000
C	-2.261566000	4.214606000	-0.044389000
C	-0.920350000	4.177680000	0.100467000
C	-0.543821000	2.767225000	0.151466000
N	-1.634477000	1.974169000	0.052365000

C	-2.694288000	2.829378000	-0.078196000
C	-4.024919000	2.462167000	-0.252782000
C	0.821732000	2.438053000	0.237153000
C	0.846447000	-0.015800000	0.204768000
C	2.991060000	1.228045000	0.227716000
C	3.675100000	0.007732000	-0.057226000
C	2.956472000	-1.214458000	-0.220412000
C	3.746902000	2.358505000	0.483328000
C	5.151238000	2.342985000	0.405196000
C	5.814319000	1.198285000	0.051802000
C	5.090848000	0.006137000	-0.190658000
C	5.755127000	-1.187054000	-0.571832000
C	5.039052000	-2.326881000	-0.816778000
C	3.641984000	-2.339423000	-0.638890000
H	-6.640593000	-1.365297000	-0.491800000
H	-6.689944000	1.323152000	-0.660000000
H	-2.743669000	0.191633000	0.162902000
H	-0.292235000	-4.975165000	0.928758000
H	-2.973266000	-4.955559000	0.776827000
H	-1.512366000	-1.216204000	-0.460890000
H	-4.780397000	-3.161856000	0.083319000
H	1.547648000	-3.332780000	0.411605000
H	-2.911833000	5.072995000	-0.129149000
H	-0.226962000	5.004240000	0.160001000
H	-4.752620000	3.256146000	-0.372271000
H	1.435804000	3.329126000	0.254009000
H	-0.217109000	0.019045000	0.393508000
H	3.269999000	3.289752000	0.756020000
H	5.702325000	3.252364000	0.613169000
H	6.894555000	1.184964000	-0.041185000
H	6.833880000	-1.172239000	-0.681132000
H	5.541353000	-3.230545000	-1.140726000
H	3.106131000	-3.255447000	-0.850712000

PhP-22,24-H

C	-5.738479000	-0.681682000	0.132649000
C	-5.738478000	0.681670000	-0.132648000
C	-4.397369000	1.095675000	-0.206631000
N	-3.611573000	0.000004000	0.000059000
C	-4.397369000	-1.095679000	0.206677000
C	-0.876651000	-4.235935000	0.404837000
C	-2.214136000	-4.234109000	0.576124000
C	-2.635493000	-2.850367000	0.404882000
N	-1.535706000	-2.036144000	0.148431000
C	-0.494989000	-2.843535000	0.139881000
C	-3.926843000	-2.419638000	0.426784000
C	1.422002000	-1.249994000	-0.236946000
C	1.421997000	1.249992000	0.236905000
C	0.880000000	-2.483929000	-0.123100000
C	-2.214146000	4.234109000	-0.576133000
C	-0.876650000	4.235927000	-0.404931000
C	-0.494992000	2.843539000	-0.139904000
N	-1.535703000	2.036142000	-0.148470000
C	-2.635499000	2.850367000	-0.404875000
C	-3.926848000	2.419635000	-0.426745000
C	0.880001000	2.483929000	0.123055000
C	0.621792000	-0.000003000	-0.000009000
C	2.863805000	1.127617000	0.522277000
C	3.565832000	-0.000002000	0.000007000
C	2.863818000	-1.127619000	-0.522281000
C	3.576646000	2.109773000	1.181872000
C	4.982300000	2.064436000	1.259296000
C	5.677787000	1.054302000	0.648360000
C	4.987396000	0.000002000	0.000021000
C	5.677804000	-1.054293000	-0.648304000
C	4.982330000	-2.064423000	-1.259266000
C	3.576677000	-2.109765000	-1.181873000
H	-6.590905000	-1.331555000	0.256001000
H	-6.590904000	1.331540000	-0.256019000
H	-2.591944000	-0.000004000	0.000035000
H	-0.193807000	-5.072204000	0.446262000
H	-2.870674000	-5.065405000	0.788014000

H	-4.699295000	-3.160171000	0.606661000
H	1.543838000	-3.337322000	-0.206405000
H	-2.870689000	5.065405000	-0.788004000
H	-0.193801000	5.072190000	-0.446413000
H	-4.699305000	3.160164000	-0.606618000
H	1.543845000	3.337318000	0.206355000
H	-0.062914000	0.158618000	-0.841240000
H	-0.062880000	-0.158636000	0.841249000
H	3.050235000	2.935722000	1.644023000
H	5.511140000	2.850148000	1.785492000
H	6.761997000	1.038534000	0.662758000
H	6.762013000	-1.038525000	-0.662677000
H	5.511184000	-2.850129000	-1.785458000
H	3.050281000	-2.935709000	-1.644049000

PhP-22,23-H

C	-5.868739000	-0.544949000	-0.152358000
C	-5.766689000	0.784087000	0.097159000
C	-4.340042000	1.068441000	0.200398000
N	-3.609104000	-0.020895000	0.039564000
C	-4.507568000	-1.041273000	-0.182832000
C	-0.964919000	-4.137814000	-0.403508000
C	-2.342445000	-4.134984000	-0.550575000
C	-2.780454000	-2.810447000	-0.385115000
N	-1.665487000	-2.031592000	-0.154522000
C	-0.552636000	-2.813146000	-0.154125000
C	-4.106888000	-2.339396000	-0.392723000
C	1.401454000	-1.230175000	0.212369000
C	1.485981000	1.279084000	-0.235966000
C	0.817900000	-2.443697000	0.093842000
C	-2.152565000	4.229827000	0.581012000
C	-0.817400000	4.270250000	0.417742000
C	-0.404521000	2.884271000	0.138051000
N	-1.419274000	2.052720000	0.129546000
C	-2.543084000	2.829614000	0.393077000
C	-3.830256000	2.394685000	0.423910000
C	0.972670000	2.524960000	-0.116382000
C	0.644726000	0.049248000	-0.017303000
C	2.924949000	1.111983000	-0.509508000
C	3.585800000	-0.047145000	-0.002052000
C	2.845148000	-1.159856000	0.500346000
C	3.673409000	2.082170000	-1.147081000
C	5.077020000	1.992283000	-1.217676000
C	5.734907000	0.948540000	-0.621878000
C	5.006787000	-0.093752000	0.004040000
C	5.660588000	-1.180970000	0.635896000
C	4.930271000	-2.177132000	1.228315000
C	3.524076000	-2.173762000	1.148332000
H	-6.760619000	-1.136392000	-0.299107000
H	-6.560704000	1.510287000	0.194715000
H	-0.295459000	-4.981763000	-0.461250000
H	-2.989020000	-4.976181000	-0.748251000
H	-1.763387000	-1.028709000	0.015961000
H	-4.871654000	-3.088947000	-0.569455000
H	1.457740000	-3.315670000	0.168424000
H	-2.831661000	5.041000000	0.800513000
H	-0.156473000	5.123350000	0.472489000
H	-4.585805000	3.152824000	0.610664000
H	1.651911000	3.367279000	-0.185871000
H	-0.033234000	0.240881000	0.823262000
H	-0.025152000	-0.058928000	-0.879814000
H	3.177219000	2.933045000	-1.597182000
H	5.634419000	2.769677000	-1.726395000
H	6.818040000	0.896995000	-0.631061000
H	6.744624000	-1.200574000	0.653605000
H	5.430919000	-2.988293000	1.743337000
H	2.970466000	-2.987175000	1.600860000

$PhPaH^+$

C	-5.512547000	-0.689245000	1.226288000
C	-5.512375000	0.689247000	1.226831000
C	-4.310812000	1.130272000	0.626609000

N	-3.569868000	0.000106000	0.323084000
C	-4.311101000	-1.130093000	0.625723000
C	-1.014448000	-4.063930000	-1.058113000
C	-2.339344000	-4.092183000	-0.742707000
C	-2.692530000	-2.829797000	-0.172809000
N	-1.540926000	-2.047141000	-0.192283000
C	-0.477905000	-2.791405000	-0.670050000
C	-3.927584000	-2.435149000	0.297713000
C	1.490180000	-1.231425000	-0.336850000
C	1.490492000	1.231468000	-0.337329000
C	0.861199000	-2.449166000	-0.684926000
C	-2.338994000	4.092656000	-0.740911000
C	-1.014237000	4.064255000	-1.056919000
C	-0.477750000	2.791509000	-0.669585000
N	-1.540707000	2.047226000	-0.191658000
C	-2.692125000	2.830133000	-0.171374000
C	-3.927087000	2.435477000	0.299374000
C	0.861330000	2.449241000	-0.685044000
C	0.827300000	0.000053000	-0.430416000
C	2.920037000	1.241244000	0.002625000
C	3.606169000	-0.000160000	0.135963000
C	2.919514000	-1.241393000	0.003931000
C	3.612888000	2.417538000	0.243219000
C	4.985801000	2.416547000	0.538748000
C	5.666882000	1.230787000	0.632133000
C	4.989765000	-0.000331000	0.457895000
C	5.666175000	-1.231590000	0.633972000
C	4.984439000	-2.417105000	0.542299000
C	3.611600000	-2.417745000	0.246392000
H	-6.276406000	-1.344572000	1.615937000
H	-6.276107000	1.344463000	1.616911000
H	-2.910061000	0.000340000	-0.446366000
H	-0.431128000	-4.846273000	-1.519566000
H	-3.039241000	-4.897691000	-0.905996000
H	-1.397108000	-1.307898000	0.482563000
H	-4.684252000	-3.204135000	0.392098000
H	1.504200000	-3.268210000	-0.980503000
H	-3.038872000	4.898291000	-0.903648000
H	-0.430935000	4.846635000	-1.518334000
H	-1.396777000	1.307830000	0.483001000
H	-4.683540000	3.204577000	0.394542000
H	1.504162000	3.268386000	-0.980658000
H	-0.182575000	0.000169000	-0.825878000
H	3.096750000	3.368497000	0.232079000
H	5.495399000	3.356827000	0.706861000
H	6.724923000	1.216929000	0.868262000
H	6.724201000	-1.217972000	0.870184000
H	5.493459000	-3.357417000	0.711971000
H	3.094819000	-3.368380000	0.236463000

PhPbH$^+$

C	5.767786000	-0.662031000	0.152367000
C	5.767747000	0.661858000	-0.154400000
C	4.380985000	1.050237000	-0.229683000
N	3.550926000	0.000072000	-0.000245000
C	4.381034000	-1.050154000	0.228866000
C	0.856495000	-4.194525000	0.449229000
C	2.211258000	-4.183906000	0.655037000
C	2.672933000	-2.857151000	0.428559000
N	1.559464000	-2.098987000	0.100791000
C	0.453679000	-2.872759000	0.097493000
C	3.960602000	-2.371016000	0.471382000
C	-1.439060000	-1.247571000	-0.283973000
C	-1.439162000	1.247449000	0.284435000
C	-0.895299000	-2.488010000	-0.192010000
C	2.211109000	4.184189000	-0.653876000
C	0.856396000	4.194707000	-0.447702000
C	0.453665000	2.872753000	-0.096522000
N	1.559471000	2.099001000	-0.100447000
C	2.672849000	2.857325000	-0.428134000
C	3.960511000	2.371226000	-0.471452000
C	-0.895301000	2.487914000	0.192928000

C	-0.641252000	-0.000016000	0.000100000
C	-2.871584000	1.107863000	0.570567000
C	-3.567031000	-0.000054000	-0.000143000
C	-2.871391000	-1.108013000	-0.570533000
C	-3.584047000	2.070786000	1.259799000
C	-4.987927000	2.018815000	1.337667000
C	-5.677687000	1.028434000	0.687811000
C	-4.986780000	-0.000048000	-0.000418000
C	-5.677431000	-1.028539000	-0.688922000
C	-4.987439000	-2.018965000	-1.338446000
C	-3.583572000	-2.070940000	-1.260029000
H	6.612730000	-1.318063000	0.299561000
H	6.612662000	1.317776000	-0.302256000
H	0.179805000	-5.029938000	0.537736000
H	2.842850000	-5.010173000	0.944314000
H	1.668687000	-1.138592000	-0.200829000
H	4.735838000	-3.096570000	0.695314000
H	-1.560454000	-3.337476000	-0.287102000
H	2.842612000	5.010606000	-0.942921000
H	0.179675000	5.030172000	-0.535511000
H	1.668896000	1.138446000	0.200592000
H	4.735671000	3.096919000	-0.695180000
H	-1.560431000	3.337367000	0.288313000
H	0.010607000	-0.192706000	0.864366000
H	0.010215000	0.192838000	-0.864464000
H	-3.060260000	2.881758000	1.751348000
H	-5.519426000	2.781935000	1.892094000
H	-6.761581000	1.010600000	0.700880000
H	-6.761318000	-1.010650000	-0.702477000
H	-5.518723000	-2.782093000	-1.893065000
H	-3.059608000	-2.881913000	-1.751381000

PhPcH$^+$

C	5.742908000	-0.729576000	-0.110266000
C	5.771923000	0.618992000	-0.402278000
C	4.446242000	1.094755000	-0.313998000
N	3.639079000	0.050394000	-0.005967000
C	4.397998000	-1.095250000	0.136105000
C	0.806618000	-4.157883000	0.697791000
C	2.173009000	-4.126469000	0.829039000
C	2.625694000	-2.838658000	0.439059000
N	1.488911000	-2.113254000	0.089630000
C	0.384886000	-2.890856000	0.216242000
C	3.927288000	-2.380349000	0.407742000
C	-1.471247000	-1.265149000	-0.253087000
C	-1.429516000	1.233099000	0.281808000
C	-0.960252000	-2.514651000	-0.092626000
C	2.234219000	4.221224000	-0.443815000
C	0.912551000	4.203374000	-0.217650000
C	0.543786000	2.791545000	-0.000801000
N	1.590055000	1.989178000	-0.078584000
C	2.677740000	2.830847000	-0.346697000
C	3.971242000	2.432920000	-0.465643000
C	-0.833285000	2.450328000	0.250213000
C	-0.655067000	-0.021736000	-0.019068000
C	-2.875601000	1.130622000	0.521811000
C	-3.580114000	0.027183000	-0.046579000
C	-2.894938000	-1.111049000	-0.568064000
C	-3.589885000	2.116781000	1.175915000
C	-4.996040000	2.095458000	1.214122000
C	-5.688884000	1.107672000	0.563820000
C	-4.999465000	0.052364000	-0.083032000
C	-5.692748000	-0.981254000	-0.761500000
C	-5.008203000	-2.006148000	-1.361546000
C	-3.608530000	-2.083490000	-1.242850000
H	6.574647000	-1.417536000	-0.092828000
H	6.630601000	1.222858000	-0.649885000
H	2.668375000	0.232555000	0.244425000
H	0.140027000	-4.975737000	0.921751000
H	2.818383000	-4.913162000	1.190147000
H	1.502476000	-1.259874000	-0.449986000
H	4.691101000	-3.125102000	0.605001000

H	-1.641328000	-3.355665000	-0.135845000
H	2.877492000	5.063881000	-0.649485000
H	0.220105000	5.032046000	-0.198659000
H	4.721819000	3.185502000	-0.676353000
H	-1.469646000	3.318209000	0.373222000
H	-0.033212000	0.182563000	-0.902359000
H	0.047804000	-0.183193000	0.806850000
H	-3.066473000	2.925631000	1.670538000
H	-5.526114000	2.879830000	1.739578000
H	-6.772874000	1.110996000	0.547850000
H	-6.775314000	-0.941159000	-0.804873000
H	-5.541143000	-2.775053000	-1.906500000
H	-3.087021000	-2.919707000	-1.693148000

$PhPaH_2^{2+}$

C	5.547047000	0.697438000	1.173544000
C	5.547103000	-0.697785000	1.173202000
C	4.376680000	-1.123424000	0.539419000
N	3.640944000	0.000000000	0.206699000
C	4.376604000	1.123281000	0.539943000
C	1.009031000	4.006318000	-1.085577000
C	2.345545000	4.062945000	-0.834310000
C	2.736531000	2.833411000	-0.208289000
N	1.567042000	2.056354000	-0.106328000
C	0.505835000	2.763001000	-0.575100000
C	3.983595000	2.441643000	0.179975000
C	-1.503684000	1.281355000	-0.123404000
C	-1.503532000	-1.281321000	-0.122941000
C	-0.872958000	2.434841000	-0.510423000
C	2.345550000	-4.062540000	-0.835810000
C	1.008936000	-4.005907000	-1.086734000
C	0.505821000	-2.762872000	-0.575632000
N	1.567073000	-2.056333000	-0.106834000
C	2.736578000	-2.833277000	-0.209371000
C	3.983692000	-2.441613000	0.178947000
C	-0.873006000	-2.434744000	-0.510300000
C	-0.740457000	0.000090000	0.118718000
C	-2.946353000	-1.255784000	0.066939000
C	-3.627587000	-0.000075000	0.153988000
C	-2.946545000	1.255701000	0.065830000
C	-3.674679000	-2.431260000	0.217334000
C	-5.063778000	-2.423405000	0.389651000
C	-5.735422000	-1.228195000	0.435395000
C	-5.036697000	-0.000143000	0.335290000
C	-5.735693000	1.227856000	0.434005000
C	-5.064318000	2.423181000	0.386936000
C	-3.675216000	2.431173000	0.214830000
H	6.300074000	1.348744000	1.591131000
H	6.300178000	-1.349233000	1.590484000
H	3.014155000	0.000166000	-0.590231000
H	0.396818000	4.751994000	-1.569692000
H	3.032325000	4.856002000	-1.092941000
H	1.481439000	1.329600000	0.589774000
H	4.759723000	3.199476000	0.186106000
H	-1.498205000	3.264239000	-0.813108000
H	3.032314000	-4.855446000	-1.094946000
H	0.396666000	-4.751439000	-1.570996000
H	1.481559000	-1.329867000	0.589585000
H	4.759829000	-3.199442000	0.184738000
H	-1.498422000	-3.264105000	-0.812767000
H	0.162192000	0.000063000	-0.495877000
H	-0.444278000	0.000331000	1.184221000
H	-3.168616000	-3.387340000	0.241227000
H	-5.596103000	-3.359214000	0.500075000
H	-6.811101000	-1.205725000	0.569773000
H	-6.811374000	1.205303000	0.568364000
H	-5.596877000	3.358988000	0.496236000
H	-3.169464000	3.387435000	0.237770000

$PhPbH_2^{2+}$

C	-5.416226000	-0.780792000	1.446310000
C	-5.513345000	0.587228000	1.346458000

C	-4.353147000	1.065844000	0.681300000
N	-3.549970000	-0.049407000	0.425328000
C	-4.211527000	-1.175600000	0.810442000
C	-0.984519000	-4.006262000	-1.165910000
C	-2.254678000	-4.084744000	-0.722258000
C	-2.597587000	-2.827133000	-0.088239000
N	-1.473155000	-2.000390000	-0.201046000
C	-0.435494000	-2.715303000	-0.785273000
C	-3.796976000	-2.492170000	0.462665000
C	1.536484000	-1.161555000	-0.492998000
C	1.518841000	1.280011000	-0.256659000
C	0.882783000	-2.370422000	-0.896934000
C	-2.552142000	3.783924000	-1.194194000
C	-1.185040000	3.772086000	-1.465553000
C	-0.604072000	2.777707000	-0.680134000
N	-1.595769000	2.125840000	-0.016842000
C	-2.817618000	2.750489000	-0.287758000
C	-4.053592000	2.333758000	0.230298000
C	0.855717000	2.632636000	-0.394974000
C	0.871848000	0.093213000	-0.528628000
C	2.909915000	1.266080000	0.118850000
C	3.591492000	0.022789000	0.180800000
C	2.923266000	-1.195772000	-0.110572000
C	3.626896000	2.440192000	0.406730000
C	4.976739000	2.414268000	0.746554000
C	5.640704000	1.205226000	0.817873000
C	4.964350000	-0.003502000	0.543003000
C	5.641112000	-1.239816000	0.629994000
C	4.977288000	-2.427820000	0.386672000
C	3.634182000	-2.403408000	0.029389000
H	-6.124299000	-1.459194000	1.896911000
H	-6.309622000	1.215642000	1.718166000
H	-2.884312000	-0.056838000	-0.339207000
H	-0.428143000	-4.755541000	-1.709553000
H	-2.947473000	-4.905280000	-0.838577000
H	-1.275614000	-1.308500000	0.511021000
H	-4.530633000	-3.279589000	0.580660000
H	1.504982000	-3.162212000	-1.291901000
H	-3.299376000	4.431829000	-1.627964000
H	-0.649013000	4.431750000	-2.130245000
H	-1.440543000	1.554795000	0.803987000
H	-4.855359000	3.065329000	0.236376000
H	1.035835000	3.189056000	0.533990000
H	1.383988000	3.190869000	-1.173315000
H	-0.143848000	0.123438000	-0.907473000
H	3.136315000	3.404590000	0.372068000
H	5.497071000	3.338935000	0.959746000
H	6.690147000	1.171137000	1.090215000
H	6.691187000	-1.245834000	0.902661000
H	5.494879000	-3.373684000	0.479259000
H	3.132169000	-3.349902000	-0.122447000

$PhPcH_2^{2+}$

C	5.049550000	0.780539000	1.743543000
C	4.987454000	-0.616607000	1.866884000
C	4.116928000	-1.083701000	0.898223000
N	3.574867000	-0.005558000	0.248560000
C	4.172269000	1.158913000	0.733279000
C	1.029041000	4.044239000	-1.297778000
C	2.356169000	4.065120000	-0.986563000
C	2.687852000	2.827055000	-0.348616000
N	1.513335000	2.059227000	-0.343415000
C	0.478961000	2.803814000	-0.835307000
C	3.864283000	2.452967000	0.239656000
C	-1.498452000	1.293711000	-0.406316000
C	-1.460063000	-1.166873000	-0.442509000
C	-0.890116000	2.495298000	-0.765522000
C	2.404179000	-3.922062000	-1.104817000
C	1.089156000	-3.899208000	-1.465240000
C	0.467152000	-2.767963000	-0.831064000
N	1.482015000	-2.152128000	-0.075457000
C	2.622684000	-2.830652000	-0.213083000

C	3.897611000	-2.500972000	0.476709000
C	-0.826625000	-2.375652000	-0.941455000
C	-0.814808000	0.039914000	-0.572989000
C	-2.845578000	-1.230205000	-0.016546000
C	-3.536980000	-0.006988000	0.193426000
C	-2.894261000	1.251192000	0.016587000
C	-3.495424000	-2.439128000	0.213167000
C	-4.836959000	-2.478877000	0.611608000
C	-5.526313000	-1.304012000	0.800932000
C	-4.893702000	-0.051221000	0.609267000
C	-5.589559000	1.156596000	0.841915000
C	-4.951595000	2.369252000	0.703131000
C	-3.612394000	2.412495000	0.307846000
H	5.635785000	1.459552000	2.344158000
H	5.534375000	-1.229796000	2.566631000
H	3.170087000	-0.049703000	-0.678625000
H	0.455912000	4.815768000	-1.789321000
H	3.067453000	4.851980000	-1.191738000
H	1.367874000	1.346835000	0.359029000
H	4.621477000	3.219705000	0.364421000
H	-1.527515000	3.344195000	-0.975562000
H	3.164413000	-4.626094000	-1.407755000
H	0.574628000	-4.578918000	-2.129623000
H	1.333803000	-1.405294000	0.593190000
H	3.975772000	-3.148873000	1.359841000
H	4.711938000	-2.819971000	-0.185243000
H	-1.464619000	-3.062279000	-1.492036000
H	0.168478000	0.056254000	-1.031910000
H	-2.961987000	-3.377127000	0.105640000
H	-5.321280000	-3.432279000	0.777952000
H	-6.564215000	-1.322229000	1.114414000
H	-6.628355000	1.113911000	1.150086000
H	-5.477989000	3.291482000	0.912658000
H	-3.130333000	3.380205000	0.255773000
